# Identification guide to some Diaptomid species (Crustacea, Copepoda, Calanoida, Diaptomidae) of “de la Plata” River Basin (South America)

**DOI:** 10.3897/zookeys.497.8091

**Published:** 2015-04-20

**Authors:** Gilmar Perbiche-Neves, Geoffrey Allan Boxshall, Daniel Previattelli, Marcos Gomes Nogueira, Carlos Eduardo Falavigna da Rocha

**Affiliations:** 1 Universidade Estadual do Centro Oeste – UNICENTRO, Departamento de Biologia, campus CEDETEG, Rua Simeão Camargo Varela de Sá, 03, Vila Carli, CEP 85040-080, Guarapuava, PR, Brazil; 2 The Natural History Museum – NHM, Department of Life Sciences, Cromwell Road, London SW7 5BD, United Kingdom; 3 Universidade de São Paulo – USP, IB, Departamento de Zoologia, Rua do Matão, travessa 14, n. 321, CEP 05508-900, São Paulo, SP, Brasil; 4 Universidade Estadual Paulista – UNESP, IBB, Departamento de Zoologia. Distrito de Rubião Júnior s/n, CEP 18618-970, Botucatu, SP, Brasil

**Keywords:** Copepoda, plankton, taxonomy, rivers, reservoirs, *Notodiaptomus*, macro scale distributions

## Abstract

An identification guide is presented for species of calanoid copepod family Diaptomidae from “de la Plata” River Basin (Argentina, Brazil, Bolivia, Paraguay and Uruguay). It was based on material collected during the summer and winter of 2010 from 43 sites across the eastern part and the lower stretches of this basin, the second largest in South America and the fourth in the world. The guide contains identification keys and species diagnoses for males and females, richly supported by scanning electronic micrographs and/or line drawings of 19 species. It also includes some general remarks on the taxonomy and phylogenetic relationships of these species. The key was adjusted to be useful for these species only, with separate keys for each sex, and is the first for females of South America. One species classified herein as *incertae sedis* was not included in the analysis. At least ten other species have previously been recorded in the basin but were not present in our samples. This is the first attempt to compile comprehensive taxonomic information on this group of copepods in this region, and it is expected to become a useful tool for biologists and young taxonomists interested in the crustacean biota of the Neotropical region.

## Introduction

Copepods are typically small crustaceans that are widely distributed in virtually all aquatic habitats from hot springs to glacial meltwater pools, and from deep ocean trenches to high altitude lakes (Boxshall and Halsey 2004). In terms of abundance, copepods are the dominant group in the zooplankton community both in the oceans and in continental waters, where they share the role of mass and energy transfer between producers and higher level consumers with rotifers and other crustaceans, such as cladocerans (Margalef 1983).

In continental or inland waters, copepods are predominantly represented by three orders: Calanoida, Cyclopoida, and Harpacticoida. In general terms, members of the Calanoida tend to be planktonic forms whereas the Cyclopoida are represented by planktonic as well as littoral, epibenthic and benthic species. Harpacticoids are essentially benthic and interstitial. We use the term planktonic to include the potamoplankton, referring to organisms found in rivers.

All the calanoids collected during our study belong to the subfamily Diaptominae, the most diverse taxon within the family Diaptomidae (Boxshall and Defaye 2008). Recent phylogenetic analyses of the order Calanoida (Bradford-Grieve et al. 2010; Blanco-Bercial et al. 2011) continue to classify the Diaptomidae within a large superfamily (referred to either as the Centropagoidea or the Diaptomoidea) which is placed in a relatively basal position within the order, as in most schemes published since the widely adopted system proposed by Andronov (1974).

In South America, copepods of continental waters began to be studied from the 1890s, and several major works appeared in the 1920s and 1930s (e.g. Wright 1927, 1936, Brehm 1933, 1938). Since 1950 there has been greater continuity in studies (such as those by the Argentinean researchers Raul Ringuelet and Juan Paggi), and it is now easier to highlight the gaps in our knowledge of the regional copepod fauna. The literature on the inland water copepods of Ecuador, Peru and Bolivia is relatively limited, while in other countries, like Argentina, Brazil, Venezuela, and Chile, there are large geographical areas that remain unsurveyed and the information remains patchy for some large regions due to under-sampling. Biogeographical analyses of South and Central American calanoids can be found in Menu-Marque et al. (2000), Suárez-Morales (2003), and Suárez-Morales et al. (2005).

Although some of the studies were made many years ago, we found that the following papers, used jointly, are quite useful for identifying diaptomids in the region: Wright (1927, 1937, 1938), Brehm (1933), Paggi (1976, 2001), Matsumura-Tundisi (1986), and Santos-Silva et al. (1989, 1999, 2013). Based on these and other studies, traditional morphological characters with taxonomical relevance (i.e. the structure of the fifth leg of both sexes, the armature of the geniculate right antennule (A1R) of males, and the features of the female genital double-somite, among others) were used in this study since they enable accurate, reliable identifications. The terminology adopted for these structures was based on Huys and Boxshall (1991) and Santos-Silva et al. (1999). This study aims to provide identification keys and species diagnoses for males and females of the known species of the calanoid copepod family Diaptomidae from “de la Plata” River Basin, based on material collected during the summer and winter of 2010 from 43 sites across the eastern part and the lower stretches of this basin. As the original descriptions are generally published before 1950, and the illustrations often lacked details, the 19 species identified were redescribed, focusing mostly on the morphology of the male, and illustrated, including scanning electronic micrographs and/or line drawings. Some general remarks on the taxonomy and phylogenetic relationships of these species are added. This guide will help to complete the information on the rich diaptomid fauna present in this basin, in South America, and to update the taxonomy of the species.

## Materials and methods

**Figure 1.**
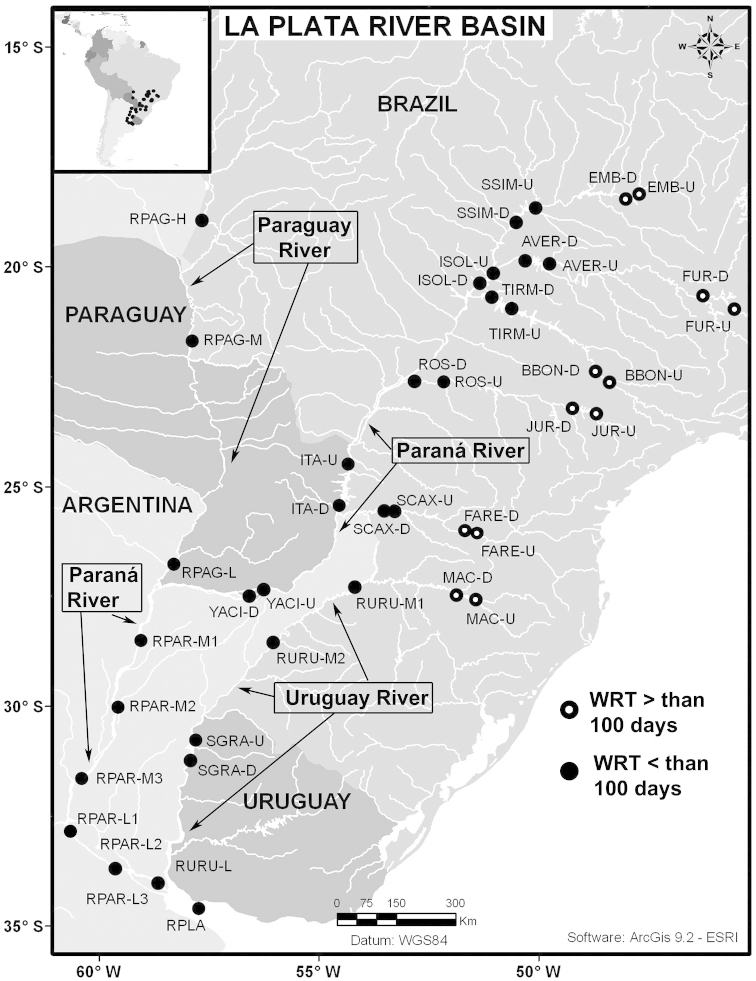
Map of sampling sites in de la Plata river basin. Reservoirs sampled included the first and the last in each regulated river as well as dam-free lotic stretches. Symbols differentiate between sites with water retention times (WRT) greater than (○) or less than (●) 100 days.

Net zooplankton samples were taken across “de la Plata” river basin in lotic stretches and in reservoirs (upstream to dam – intermediate zone –, and close to dam – lentic zone) (Figure 1 and Table 1) in two periods during 2010, summer (January and February) and winter (June and July).

**Table 1.** List of sampling sites. For each site a code is provided based on a 3 or 4 letter abbreviation of the name of the reservoir or river, and a 1 letter suffix. The suffixes used for reservoirs are: U = upstream and D = dam, and for rivers: H = high stretch, M = middle stretch, L = low stretch). Country abbreviations are: ARG = Argentina, BOL = Bolivia, BRA = Brazil, PAR = Paraguay, and URU = Uruguay. Geographical coordinates and altitude (m.a.s.l.) are also given.

N°	Codes	River/Reservoir	Coordinates	Alt. (m)
1	EMB-U	Emborcação Reservoir – BRA	18°22'40.47"S, 47°44'3.58"W	634
2	EMB-D	Emborcação Reservoir – BRA	18°29'33.09"S, 47°58'17.22"W	648
3	SSIM-U	São Simão Reservoir – BRA	18°40'22.54"S, 50° 4'17.76"W	402
4	SSIM-D	São Simão Reservoir – BRA	18°59'15.59"S, 50°30'18.93"W	406
5	FUR-U	Furnas Reservoir – BRA	20°58'35.58"S, 45°31'24.18"W	771
6	FUR-D	Furnas Reservoir – BRA	20°39'36.51"S, 46°18'12.16"W	769
7	AVER-U	Água Vermelha Reservoir – BRA	19°55'42.17"S, 49°45'5.31"W	388
8	AVER-D	Água Vermelha Reservoir – BRA	19°52'03.73"S, 50°19'28.77"W	388
9	BBON-U	Barra Bonita Reservoir – BRA	22°40'04.08"S, 48°21'05.01"W	463
10	BBON-D	Barra Bonita Reservoir – BRA	22°31'43.07"S, 48°31'26.05"W	454
11	TIRM-U	Três Irmãos Reservoir – BRA	20°57'21.57"S, 50°36'34.83"W	320
12	TIRM-D	Três Irmãos Reservoir – BRA	20°41'57.09"S, 51°05'58.43"W	326
13	JUR-U	Jurumirim Reservoir – BRA	23°19'25.07"S, 48°42'11.07"W	572
14	JUR-D	Jurumirim Reservoir – BRA	23°13'41.07"S, 49°13'28.03"W	566
15	ROS-U	Rosana Reservoir – BRA	22°36'28.27"S, 52°09'43.75"W	262
16	ROS-D	Rosana Reservoir – BRA	22°36'04.71"S, 52°49'48.15"W	261
17	FAR-U	Foz do Areia Reservoir – BRA	26°03'41.64"S, 51°24'02.25"W	754
18	FAR-D	Foz do Areia Reservoir – BRA	25°59'57.06"S, 51°38'52.27"W	749
19	SCAX-U	Salto Caxias Reservoir – BRA	25°30'32.11"S, 53°18'24.26"W	333
20	SCAX-D	Salto Caxias Reservoir – BRA	25°31'50.96"S, 53°28'45.76"W	319
21	ISOL-U	Ilha Solteira Reservoir – BRA	20°10'29.60"S, 51° 2'7.06"W	332
22	ISOL-D	Ilha Solteira Reservoir – BRA	20°22'10.87"S, 51°20'37.65"W	321
23	ITA-U	Itaipu Reservoir – BRA/PAR	24°29'10.77"S, 54°19'42.38"W	217
24	ITA-D	Itaipu Reservoir – BRA/PAR	25°25'09.67"S, 54°32'14.47"W	220
25	YACI-U	Yaciretá Reservoir – ARG/PAR	27°24'24.13"S, 56°15'19.86"W	71
26	YACI-D	Yaciretá Reservoir – ARG/PAR	27°30'9.12"S, 56°31'56.69"W	78
27	RPAR-M1	Paraná River – middle stretch – ARG	28°30'10.12"S, 59°03'03.24"W	43
28	RPAR-M2	Paraná River – middle stretch – ARG	30°01'07.73"S, 59°33'50.86"W	26
29	RPAR-M3	Paraná River – middle stretch – ARG	31°38'29.94"S, 60°23'21.53"W	21
30	RPAR-L1	Paraná River – low stretch – ARG	32°44'02.61"S, 60°43'23.95"W	5
31	RPAR-L2	Paraná River – low stretch – ARG	33°41'19.94"S, 59°37'30.79"W	5
32	RPAR-L3	Paraná River – low stretch – ARG	33°56'31.07"S, 58°27'46.80"W	5
33	RPLA	Rio de la Plata – URU/ARG	34°26'49.57"S, 57°36'27.99"W	1
34	RPAG-H	Paraguay River – high stretch – BOL/BRA	18°58'07.73"S, 57°38'55.75"W	94
35	RPAG-M	Paraguay River – middle stretch – PAR/BRA	21°41'09.18"S, 57°52'59.85"W	71
36	RPAG-L	Paraguay River – low stretch – ARG/PAR	26°51'15.42"S, 58°19'21.45"W	54
37	MAC-U	Machadinho Reservoir – BRA	27°32'26.71"S, 51°37'52.31"W	476
38	MAC-D	Machadinho Reservoir – BRA	27°29'27.77"S, 51°46'26.50"W	484
39	RURU-M1	Uruguay – middle stretch – ARG/BRA	27°17'15.23"S, 54°11'31.66"W	112
40	RURU-M2	Uruguay – middle stretch – ARG/BRA	28°32'38.40"S, 56° 1'24.69"W	54
41	SGRA-U	Salto Grande Reservoir – URU/ARG	30°46'27.52"S, 57°47'55.53"W	33
42	SGRA-D	Salto Grande Reservoir – URU/ARG	31°15'31.41"S, 57°55'33.66"W	34
43	RURU-L	Uruguay River – low stretch – URU/ARG	33°48'07.39"S, 58°26'07.48"W	8

The samples were collected by vertical hauls with a conical plankton net with a 68 µm mesh, equipped with an anti-reflux adaptation following Tranter and Heron (1965, 1967). The samples were fixed in 4% formalin solution for qualitative analysis and with 2.5% glutaraldehyde for scanning electron microscopy (SEM).

Back in the laboratory, the copepods were sorted and analysed under a stereo- microscope (Zeiss Stemi SV6 and Zeiss Discovery V20), or a binocular microscope (Zeiss Standard 20 and 25). Copepods were dissected with fine needles and semi-permanent slides were made in glycerine or in 70% lactophenol. Only adults were analysed.

Copepods were identified with the aid of a number of publications dealing with the taxonomy of Diaptomidae (Wright 1927, 1936, 1938, 1939, Brehm 1933, 1938, Kiefer 1933, 1936, Ringuelet and Martinez de Ferrato 1967, Brandorff 1973, Dussart 1984, Dussart and Frutos 1985, 1986, Matsumura-Tundisi 1986, Santos-Silva et al. 1989, Santos-Silva 2000; Cicchino et al. 2001, Paggi 2001, 2006, and Previattelli 2006), and the identity of problematic taxa was confirmed by direct consultations with specialists. This guide includes only those species that had been identified with certainty.

All original zooplankton samples are deposited in the Continental Water Microcrustacean Collection–CMAC, Department of Zoology, University of the State of São Paulo–UNESP, Botucatu campus. Vouchers of some species were deposited in the Museum of Zoology, University of São Paulo–MZUSP (São Paulo, Brazil), with registration numbers as follows: *Argyrodiaptomus azevedoi* (MZUSP 32928); *Argyrodiaptomus falcifer* (MZUSP 32929); *Argyrodiaptomus denticulatus* (MZUSP28393); *Notodiaptomus coniferoides* (MZUSP28389); *Notodiaptomus carteri* (MZUSP28390); *Notodiaptomus santafesinus* (MZUSP28391); *Odontodiaptomus thomseni* (MZUSP28392); *Notodiaptomus anisitsi* (MZUSP 32930); *Notodiaptomus cearensis* (MZUSP 32931); *Notodiaptomus conifer* (MZUSP 32932); *Notodiaptomus dentatus* (MZUSP 32933); *Notodiaptomus henseni* (MZUSP 32934); *Notodiaptomus iheringi* (MZUSP 32935); *Notodiaptomus incompositus* (MZUSP 32936), and *Notodiaptomus spiniger* (MZUSP 32937).

Vouchers of the species “Diaptomus” curvatus and “Diaptomus” frutosae were deposited in other collections including the National Institute of Amazonian Research–INPA (Manaus, Brazil), the Bernardino Rivadalvia Museum (Argentina), and the Natural History Museum (UK) (Perbiche-Neves et al. 2013).

**Figure 2.**
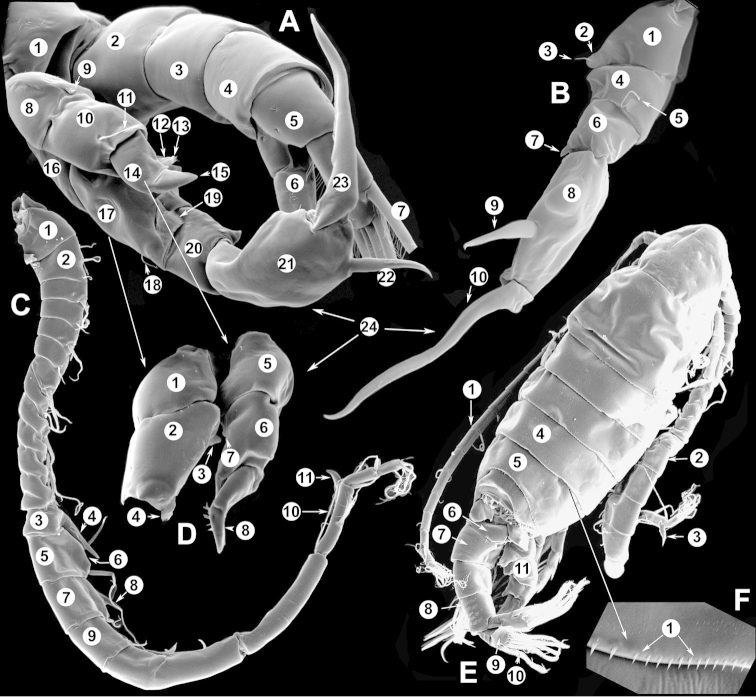
Key to the morphological structures of adult male diaptomids. **A** antero-lateral view of posterior end of prosome, urosome and caudal ramus from left side showing with left leg 5 (P5L) attached, illustrating characters A1 to A23 (Table 2) **B** right fifth leg (P5R), illustrating characters B1–10 (Table 2) **C** Right geniculate antennule (A1R) illustrating characters C1–11 (Table 2) **D** Basal part of fifth legs (P5), illustrating characters D1–8 (Table 2) **E** habitus in dorsal view, illustrating features of body tagmata and caudal rami (Table 2, characters E1–11) **F** Dorsal view of posterior margin of Ped4, showing row of spinules.

**Figure 3.**
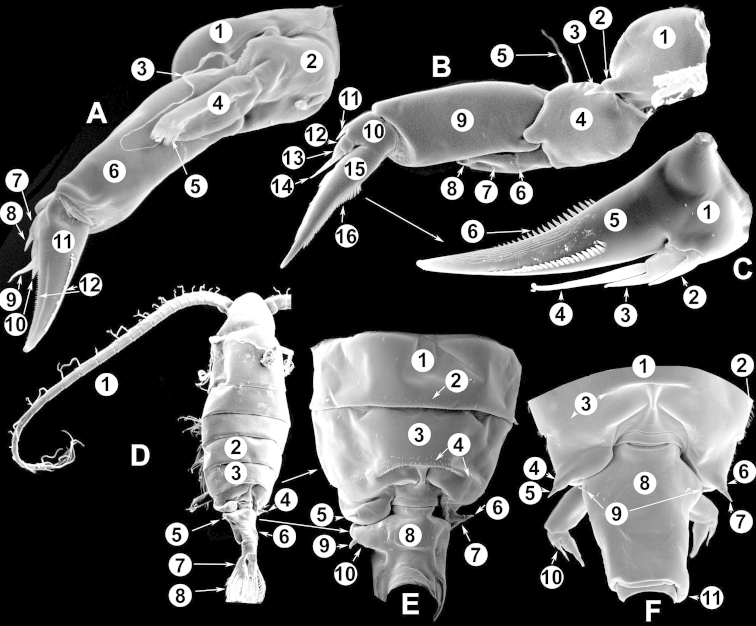
Key to the morphological structures of adult female diaptomids. **A** Caudal view of left fifth leg (P5L), illustrating characters A1–12 (Table 3) **B** Frontal view of left fifth leg (P5L) illustrating characters B1–16 (Table 3) **C** Second and third exopodite segment and terminal claw of right fifth leg (P5R), illustrating characters C1–6 (Table 3) **D** habitus in dorsal view, illustrating features of body tagmata and caudal rami (Table 3, characters D1–8) **E** Dorsal posterior part of prosome and genital double-somite, illustrating characters E1–10 (Table 3) **F** Posterior part of prosome and genital double-somite, illustrating characters F1–11 (Table 3).

The keys presented in this guide were constructed using only differential characters that emerged as being consistently useful for the identification of genera and species. Similarly, the diagnoses were designed to focus on the basic features traditionally used to characterize diaptomid genera and species, as provided in the specialized taxonomic literature. The morphological structures used in this study are illustrated for both males (Figure 2) and females (Figure 3). The terminology employed for each structure and the abbreviations used in the text are given in Tables 2 and 3, which complement Figures 2 and 3. Throughout this guide these listed abbreviations have been used to make the text less repetitive and more concise. We followed the morphological nomenclature proposed by Santos-Silva et al. (1999), Previattelli (2006) and Paggi (2006). The abbreviations for sampling sites (Figure 1, Table 1) are also used throughout.

**Table 2.** Key to male characters numbered in Figure 2. Numbers are given together with the name of the morphological structure and its abbreviation (Abbrev.).

N	Structure name	Abbrev.	N	Structure name	Abbrev.
A	Distal prosome, urosome and P5		C	Right geniculate antennule	A1R
1	Pediger 5	Ped5	1	First segment of A1R	
2	Urosome somite 1	Ur1	2	Second segment of A1R	
3	Urosome somite 2	Ur2	3	Thirteenth segment of A1R	
4	Urosome somite 3	Ur3	4	Modified seta on segment 13 of A1R	
5	Urosome somite 4	Ur4	5	Fourteenth segment of A1R	
6	Caudal rami	CR	6	Spinous process on segment 14 of A1R	
7	Caudal setae	CS	7	Fifteenth segment of A1R	
8	Coxa of left P5	CxP5L	8	Spinous process on segment 15 of A1R	
9	Sensilla of basipodite of left P5		9	Sixteenth segment of A1R	
10	Basipodite of left P5	BspP5L	10	Twentieth segment of A1R	
11	Setae of basipodite of left P5		11	Spinous process on segment 20 of A1R	
12	Endopodite of left P5	EnpP5L	D	Fifth leg anterior(P5)	
13	Apical setation of endopodite of left P5		1	Coxa of right P5	CxP5R
14	First segment of exopodite of left P5	Exp1P5L	2	Basipodite of right P5	BspP5R
15	Second segment of exopodite of left P5	Exp2PrL	3	Knob processes on internal margin of BspP5R	
16	Coxa of right P5	CxP5R	4	Endopodite of right P5	EnpP5R
17	Basipodite of right P5	BspP5R	5	Coxa of left P5	CxP5L
18	Lateral seta of basipodite of right P5		6	Basipodite of left P5	BspP5L
19	Endopodite of right P5	EnpP5R	7	Knob processes on internal margin of BspP5	
20	First segment of exopodite of right P5	Exp1P5R	8	Second segment of exopodite of left P5	Exp2PrL
21	Second segment of exopodite of right P5	Exp2P5R	E	Adult male	
22	Lateral spine of Exp2P5R		1	Left antennule	A1L
23	Terminal claw of Exp2P5R		2	Right geniculate antennule	A1R
24	Fifth leg	P5	3	Spinous process on segment 20 of A1R	
B	Right P5	P5R	4	Pediger 3	Ped3
1	Coxa of right P5	CxP5R	5	Pediger 4	Ped4
2	Distal projection of CxP5R		6	Genital segment or urosome segment 1	GS or Ur1
3	Sensilla on top of distal projection of CxP5R		7	Urosome somite 2	Ur2
4	Basipodite of right P5	BspP5R	8	Rows of spinules on urosome somite 3	
5	Lateral setae of BspP5R		9	Caudal rami	CR
6	First segment of exopodite of right P5	Exp1P5R	10	Caudal setae	CS
7	Distal projection of Exp1P5R		11	Right P5	P5R
8	Second segment of exopodite of right P5	Exp2P5R	F	Border between pediger 3 and 4	
9	Lateral spine of Exp2P5R		1	Row of spinules on distal margin of pediger 3	
10	Terminal claw of Exp2P5R				

**Table 3.** Key to female characters numbered in Figure 3. Numbers are given together with the name of the morphological structure and its abbreviation (Abbrev.).

N	Structure name	Abrev.	N	Structure name	Abrev.
A	P5 Left - caudal		D	Adult female	
1	Coxa of left P5	CxP5L	1	Left antennule	A1L
2	Basipodite of left P5	BspP5L	2	Pediger 3	Ped3
3	Lateral setae of BspP5L		3	Pediger 4	Ped4
4	Endopodite of left P5	EnpP5L	4	Lateral wing of prosome pediger 5, with apical sensilla	
5	Terminal spinules on endopodite of left P5		5	Expansions of genital double somite, with sensilla	
6	First segment of exopodite of left P5	Exp1P5L	6	Genital double somite	GS
7	Second segment of exopodite of left P5	Exp2P5L	7	Caudal rami	CR
8	Lateral seta of Exp2P5L		8	Caudal setae	CS
9	External seta of Exp3P5L		E	Distal part of prosome and genital segment	
10	Internal seta of Exp3P5L		1	Prosome pediger 3	Ped3
11	Terminal claw of left P5		2	Lines of spinules on distal dorsal margin of Ped3	
12	Rows of spinules on terminal claw of left P5		3	Prosome pediger 4	Ped4
B	P5 left – frontal		4	Rows of spinules on distal margin of Ped4	
1	Coxa of left P5	CxP5L	5	Left lateral wing of pediger 5, with apical sensila	
2	Distal projection of CxP5L		6	Sensila of right lateral wing of pediger 5	
3	Sensilla on apex of distal projection of CxP5L		7	Left lateral wing of pediger 5	
4	Basipodite of right P5	BspP5R	8	Genital double somite	GS
5	Lateral seta of BspP5L		9	Sensilla at apex of right lateral expansion of GS	
6	Endopodite of left P5	EnpP5L	10	Expansions of genital segment, with sensilla	
7	Concavity in endopodite of left P5		F	Distal part of prosome and genital double somite	
8	Terminal spinules on endopodite of left P5		1	Prosome 5, with rows of spinules on distal margin	
9	First segment of exopodite of left P5	Exp1P5L	2	Dorsal/lateral setules on Ped4 and Ped5	
10	Second segment of exopodite of left P5	Exp2P5L	3	Incomplete suture between Ped4 and Ped5	
11	Lateral seta of Exp2P5L		4	Left lateral wing of Ped5	
12	Third segment of exopodite of left P5	Exp3P5L	5	Sensilla at apex of left lateral wing of Ped5	
13	External seta of Exp3P5L		6	Right lateral wing of Ped5	
14	Internal seta of Exp3P5L		7	Sensilla at apex of right lateral wing of Ped5	
15	Terminal claw of left P5		8	Genital double somite	GS
16	Rows of spinules on terminal claw of left P5		9	Sensilla on lateral margin expansion of GS	
C	Second exopodite and terminal claw		10	P5	P5
1	Second segment of exopodite of right P5	Exp2P5R	11	Right distal margin expansion of GS	
2	Lateral seta of Exp2P5R				
3	External seta of Exp3P5R				
4	Internal seta of Exp3P5R				
5	Terminal claw of left P5				
6	Rows of spinules on terminal claw of left P5				

Morphological structures were illustrated using phase contrast microscopy (Zeiss Standard) with the aid of a drawing tube. The pencil drawings were inked in nankeen ink, then scanned and corrected for smudges and other imperfections in Adobe Photoshop 7.0 in order to obtain high-quality illustrations.

We remind users of the keys that many structures illustrated in Figures 2 and 3 can be well developed in some species but absent in others. For example, the dorsal rows of spinules on the distal margin of the pedigers are present in some species but absent in others. Similarly, the spinous processes that are typically present on segments 14, 15, 16 and 20 of the male A1R can be absent in some species.

The scanning electron microscopy (SEM) was carried out in the “Electron Microscopy Center (CME)” of the University of the State of São Paulo–UNESP – Botucatu, Brazil. Material was prepared by packing each sample of individuals in hollow cylindrical polyethylene compartments, within which the copepods were washed, fixed and dehydrated. Washing was performed using 0.1 M phosphate buffer at pH 7.3 (3 washes, each for 5 min). After washing specimens were immediately fixed by immersion in 0.5% osmium tetroxide (in water) for 20 min. Dehydration was performed progressively via a graded series of ethanol as follows: 7.5%, 15%, 30% and 50% (two changes at each concentration for 5 min), then 70% (3 changes each for 10 min), and 90% and 100% (2 changes at each, for 5 min). Subsequently, the material was critical point dried in a BALZERS UNION CTD-020 equipment, using liquid carbon dioxide as the exchange medium. After drying, specimens were dissected when necessary to reveal diagnostic structures, and attached to stubs using adhesive tape. Sputter coating with 15 nm layer of gold was carried out in a BALZERS UNION MED-10 coater. Observations were made on a Philips SEM-515 microscope and images were edited in Photoshop 7.0 (Adobe).

## Results

### Keys to the common Diaptomidae of de la Plata river basin

#### Males

**Table:** 

1	BspP5 with groups of small spinules on inner margin of both or one pediger	***Argyrodiaptomus*** (5)
–	BspP5 with smooth inner margin	**2**
2	CR with semi-circular protuberance at inner distal corner	***Odontodiaptomus thomseni* (Brehm, 1933)**
–	CR without semi-circular protuberance at inner distal corner	**3**
3	Right Exp2P5 subtriangular, 1.1–1.2 times wider than long (Fig. 88B)	**“Diaptomus” frutosae Perbiche-Neves & Boxshall, 2013**
–	Right Exp2P5 >1.2 longer than wide	**4**
4	Right Exp2P5 with lateral spine inserted proximally on margin of segment	**“Diaptomus” curvatus Perbiche-Neves, Boxshall & Paggi, 2013**
–	Right Exp2p5 with lateral spine inserted in middle of segment or sub-terminally	***Notodiaptomus*** (8)
5	Lateral spine of Exp2P5R inserted at about 1/3 distance between base of segment and terminal claw (Fig. 4B, G)	***Argyrodiaptomus azevedoi* (Wright, 1935)**
–	Lateral spine of Exp2P5R inserted at more than 1/3 distance between base of segment and terminal claw	**6**
6	Insertion of lateral spine of Exp2P5R separated from base of terminal claw by gap greater than width of base of lateral spine (Fig. 16E); continuous rows of spinules present on dorsal surface of Ur4	**7**
–	Lateral spine of right Exp2P5 inserted distally very close to base of terminal claw (Fig. 20A); no dorsal spinules on Ur4; groups of small spinules and setules present on left BspP5	***Argyrodiaptomus furcatus* (Sars, 1901)**
7	Posterior margins of Ped 4 and Ped5 lacking ornamentation of spinules; A1R with spinous process on segment 14; internal margin of right BspP5 lacking proximal expansion	***Argyrodiaptomus falcifer* (Daday, 1905)**
–	Posterior margins of Ped4 and Ped5 ornamented with dorsal spinules; segment 14 of A1R lacking spinous process; internal margin of right BspP5 with proximal expansion reaching and overlapping left BspP5	***Argyrodiaptomus denticulatus* (Pesta, 1927)**
8	Lateral spine of right Exp2P5 less than 1/5 (20%) length of terminal claw (Fig. 39A)	**9**
–	Lateral spine of right Exp2P5 more than 1/5 (20%) length of terminal claw	**13**
9	Lateral spine of right Exp2P5 inserted proximally, distant from base of terminal claw (e.g. Fig. 66A)	**10**
–	Lateral spine of right Exp2P5 inserted distally, close to base of terminal claw (Fig. 35A, 39A)	***Notodiaptomus coniferoides* (Wright, 1927)**
10	First segment of A1R lacking rows of spinules	**11**
–	First segment of A1R ornamented with rows of spinules	***Notodiaptomus iheringi* (Wright, 1935)**
11	Spinous process on anterior margin of segment 15 of A1R short, not reaching proximal border of segment 16 (Fig. 32C)	**12**
–	Spinous process on anterior margin of segment 15 of A1R long (reaching about midlength of segment 16) (Fig. 35A)	***Notodiaptomus conifer* (Sars, 1901)**
12	Segment 11 of left A1 lacking setae; small granulations absent from fissure in surface of right BspP5; internal margin of right BspP5 with proximal expansion reaching and overlapping left P5	***Notodiaptomus isabelae* (Wright, 1936)**
–	Segment 11 of left A1 armed with two setae (Fig. 32D); small granulations present in fissure on surface of right BspP5; internal margin of right BspP5 without proximal expansion extending as far as left P5 but not overlapping it	***Notodiaptomus cearensis* (Wright, 1936)**
13	Lateral spine of Exp2P5R short, between 1/5 and 1/3 the length of terminal claw (Fig. 47C–K)	**14**
–	Lateral spine of Exp2P5R relatively long, more than 1/3 the length of terminal claw (Fig. 73A, F–H)	**16**
14	Proximal inner margins of left and right BspP5 lacking sclerotized semi-circular processes	**15**
–	Proximal inner margin of BspP5 bearing one large sclerotized semi-circular process on right leg and two smaller processes on same segment of left leg (Figs 47C, 49E)	***Notodiaptomus henseni* (Dahl, 1894)**
15	Right Exp1P5 longer than wide; at least four small sclerotized processes present on posterior surface of right Exp2P5 (Fig. 44A); small lateral denticle present on outermost CS near base (Fig. 44H)	***Notodiaptomus dentatus* Paggi, 2001**
–	Right Exp1P5 wider than long; no sclerotized processes present on posterior surface of right Exp2P5, but single inner margin process present (Fig. 61D); outermost CS without basal denticle but with blunt tips and reticulate ornamentation (Fig. 61B–C)	***Notodiaptomus incompositus* (Brian, 1926)**
16	Lateral spine of right Exp2P5 located about at midlength of external margin (Fig. 25B); spine curved distally, reaching almost to end of segment; segments 15 and 16 of A1R with well-developed spinous processes; dorsal spinules present on posterior margins of Ped4 and Ped5	***Notodiaptomus anisitsi* (Daday, 1905)**
–	Lateral spine of right Exp2P5 not strongly curved, located on distal margin; segments 15 and 16 of A1R lacking well developed spinous processes; posterior margins of Ped4 and Ped5 lacking ornamentation of spinules	**17**
17	Lateral spine of right Exp2P5 inserted some distance from base of terminal claw (Fig. 29C) and with apex slightly outwardly curved; at least four small granulations present on surface of right Exp2P5; modified seta on anterior margin of segment 13 of A1R reaching articulation with segment 14	***Notodiaptomus carteri* (Lowndes, 1934)**
–	Lateral spine of right Exp2P5 inserted almost adjacent to base of terminal claw (Figs 69A–B, 74A); spine straight, not outwardly curved; modified seta on anterior margin of segment 13 of A1R short, not reaching articulation with following segment 14	**18**
18	Distal margin of segment 20 of A1R with simple distally-tapering process (Fig. 74J); segment 15 of A1R with spinous process on anterior margin; mammiform process present on medial margin of right BspP5	***Notodiaptomus spiniger* (Brian, 1925)**
–	Distal margin of segment 20 of A1R with short, apically bifid process (Fig. 69J–K); segment 15 of A1R lacking spinous process on anterior margin; medial margin of right BspP5 lacking distal mammiform process	***Notodiaptomus santafesinus* (Ringuelet & Martinez de Ferrato, 1967)**

#### Females

**Table:** 

1	Lateral wings with two lobes on each side (Fig. 7A), with large sensilla at apex of inner lobe	***Argyrodiaptomus*** (2)
–	Lateral wings with one lobe on each side, with two pairs of sensillae, one large pair at apex and smaller pair on internal part of dorsal surface (Fig. 30C)	**5**
2	Posterior margins of Ped4 and Ped5 ornamented with rows of small dorsal spinules	***Argyrodiaptomus denticulatus***
–	Posterior margins of Ped4 and Ped5 without rows of spinules	**3**
3	EnpP5 long, reaching distal end of inner margin of Exp1P5 (Fig. 7B)	***Argyrodiaptomus azevedoi***
–	EnpP5 short, not reaching distal end of inner margin of Exp1P5	**4**
4	Longest distal spine on EnpP5 about half as long as endopod (Fig. 17C); swellings on GS slightly asymmetrical, with sensillae on both sides directed slightly posteriorly, at an angle of about 80–85° to body axis	***Argyrodiaptomus falcifer***
–	Longest distal spine on EnpP5 less than half length of ramus; sensillae on swellings of GS asymmetrical, left sensilla larger and more robust than right, approximately 2.8 times longer than wide and directed more strongly backwards than right sensilla	***Argyrodiaptomus furcatus***
5	GS strongly asymmetrical, with well-developed posterior lobe on right side, extending beyond distal margin of GS; small sclerotized lobes present posteriorly on dorsal surface on left side of GS (Fig. 81A) and on lateral margin on left side of Ur3 (Fig. 82B); CxP5 with small sclerotized process on distal lobe	***Odontodiaptomus thomseni***
–	Posterior lobe on right side of GS weakly developed or absent; no small processes present dorsally on left side of GS or on left lateral margin of Ur3; CxP5 without small sclerotized process on distal lobe	**6**
6	Lateral wings larger and projecting further posteriorly on left side than on right (Fig. 86A); lacking dorsal rows of spinules on posterior margin of Ped5; asymmetrical GS, approximately 2.9 times longer than wide; swellings forming hemispherical lobes, larger on left than right; BspP5 with long outer seta, greatly exceeding length of outer margin of Exp1P5	**“Diaptomus” curvatus**
–	Lateral wings of Ped5 symmetrical or slightly asymmetrical; swellings on GS without hemispherical lobes on both sides	**7**
7	Transverse row of spinules marking position of posterior margin of Ped5; GS asymmetrical, with left swelling larger than right, and hemispherical in form; sensilla on left swelling directed slightly posteriorly, sensilla on right directed anteriorly (Fig. 90A)	**“Diaptomus” frutosae**
–	These characters not combined	***Notodiaptomus*** (8)
8	Posterior margins of Ped4 and/or Ped5 with rows of spinules on dorsal surface	**9**
–	Posterior margins of Ped4 and Ped5 lacking spinule rows dorsally	**15**
9	Rows of spinules present on dorsal surface of posterior margins of both Ped4 and Ped5	**10**
–	Rows of spinules present dorsally on posterior margin of Ped5 only	**11**
10	Single row of strong spinules present on posterior margin of Ped3, irregular rows of medium-sized spinules present on Ped4, and row of small spinules present on Ped5; lateral wings of Ped5 asymmetrical and elongate, left wing curved slightly anteriorly and right wing directed posteriorly (Fig. 27A); GS with hemispherical swellings on both sides, left sensilla directed perpendicular to body axis, right sensilla directed posteriorly; EnpP5 short, not reaching middle of inner margin of Exp1P5 (Fig. 27B)	***Notodiaptomus anisitsi***
–	Multiple rows of small spinules present on posterior margin of Ped4; lateral wings of Ped5 slightly asymmetrical, not elongate; GS with hemispherical swelling only on left side, left sensilla directed posteriorly; EnpP5 long, reaching beyond middle of inner margin of Exp1P5 (Fig. 76B)	***Notodiaptomus spiniger***
11	Irregular and multiple rows of hair-like spinules present on dorsal and lateral surfaces of Ped5	***Notodiaptomus henseni***
–	Simple row or rows of spinules present dorsally along posterior margin of Ped5, spinules short and robust, not hair-like	**12**
12	Row of small spinules present, marking plane of incomplete suture between Ped4 and Ped5; lateral wings of Ped5 asymmetrical, projecting laterally (Fig. 63A) triangular in shape, and larger on left than right	***Notodiaptomus incompositus***
–	Lateral wings of Pr5 not projecting laterally; asymmetrical in shape	**13**
13	Lateral wings of Ped5 asymmetrical, left sensilla directed posteriorly (Fig. 45A); GS asymmetrical, approximately 1.5 times longer than wide; right sensilla about 2.3–2.5 times longer than wide, slightly curved anteriorly; right lateral margin of GS sinuous, with indentation in posterior half and with small posterior lobe present postero-distally; lateral seta of BspP5 reaching about to end of outer margin of Enp1P5; internal seta on tip of Exp3P5 about 4 times longer than external seta	***Notodiaptomus dentatus***
–	Lateral wings symmetrical or slightly asymmetrical; sensilla on lateral wing on left side of Ped5 directed posterolaterally, not posteriorly; right margin of GS lacking postero-distal lobe; internal seta on tip of Exp3P5 less than 4 times as long as external seta	**14**
14	Complete single row and short double row of spinules present dorsally near posterior margin of Ped4 (Figs 58A, 59A); lateral wings of Ped5 slightly asymmetrical (Fig. 58A); GS symmetrical (Fig. 58A) sensilla on left swelling of GS about 3.2 times longer than wide; margin posterior to swelling on right side of GS smooth; outer seta of BspP5 long (Fig. 58B), reaching to end of outer margin of Exp1P5	***Notodiaptomus iheringi***
–	Lines of spinules present on Ped4 border; lateral wings of Ped5 clearly asymmetrical, with apical (outer) sensilla about 2.2 to 2.3 times longer than wide; inner sensilla on lateral wings well developed (Fig. 67A); GS asymmetrical, swelling on left side hemispherical, carrying sensilla twice as long as wide; swelling on right side triangular in shape, with similar sensilla; right margin with marked indentation just posterior to swelling, posterior margin smooth; sensilla on CxP5 about as wide as long; outer seta on BspP5 reaching middle of outer margin of Exp1P5	***Notodiaptomus isabelae***
15	Dorsal projection present on Ped4 (Fig. 42A–B)	**16**
–	Ped4 lacking such dorsal projection	**18**
16	Inner right sensilla on lateral wing of Ped5 short, setule-like; GS asymmetrical, right sensilla about 2.3 times longer than wide, inserted on hemispherical lobe; right margin of GS with rounded lobe at posterior end (Fig. 30C)	***Notodiaptomus carteri***
–	Inner right sensilla of Ped5 not setule-like; GS right sensilla present but inserted in different position, no hemispherical lobe present on right side of GS at insertion of sensilla	**17**
17	Lateral wings of Ped5 projecting posteriorly; inner sensilla on Ped5 short with broad base (Fig. 42A); GS about twice as long as wide; left sensilla on GS slightly curved and directed posteriorly, right sensilla directed perpendicular to body axis; sensilla on CxP5 longer than wide (Fig. 41B); external seta on Exp3P5 about one third length of internal seta	***Notodiaptomus coniferoides***
–	Lateral wings of Ped5 not projecting posteriorly; position of inner sensilla on Ped5 variable; GS about 1.6 times longer than wide; sensilla on left swelling of GS directed slightly posteriorly, right sensilla directed slightly anteriorly; sensilla on CxP5 about 1.5 times longer than wide; external seta on Exp3P5 about half length of internal seta	***Notodiaptomus santafesinus***
18	Sensillae on both swellings on GS directed perpendicular to body axis; right margin of GS with small rounded lobe at posterior extremity (Fig. 33A); sensilla on CxP5 longer than wide; external seta on Exp3P5 about one third length of inner seta	***Notodiaptomus cearensis***
–	Sensilla on right swelling on GS directed posteriorly; right margin of GS without posterior lobe (Fig. 37A); sensilla on CxP5 about 1.5 times longer than wide; external seta on Exp3P5 about 1/5 (20%) length of internal seta	***Notodiaptomus conifer***

### Species diagnoses

#### Genus *Argyrodiaptomus* Brehm, 1933

##### Argyrodiaptomus azevedoi

Taxon classificationAnimaliaCalanoidaDiaptomidae

(Wright, 1935)

**Figure 4.**
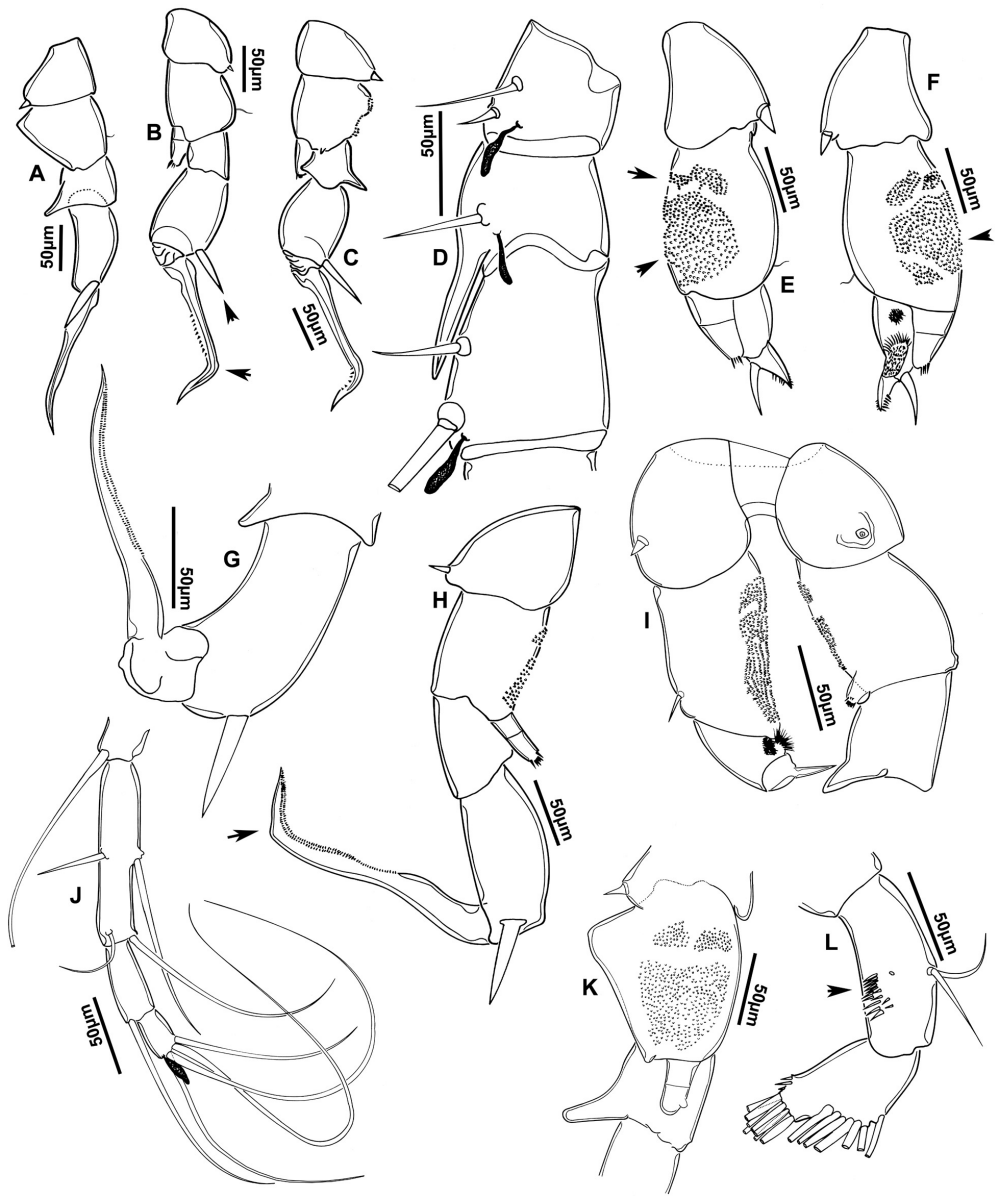
*Argyrodiaptomus azevedoi* male. **A, B, C** Different views of Right P5 **D** Segments 12, 13 and 14 of A1R **E, F** Different views of Left P5, showing details of spinular ornamentation **G** Right Exp2P5 **H** Right P5 **I** Basal segments of right and left P5 **J** Segments 20, 21 and 22 of A1R **K** Detail showing spinules on BspP5R **L** Endopod of A2, showing pore and spinular ornamentation.

**Figure 5.**
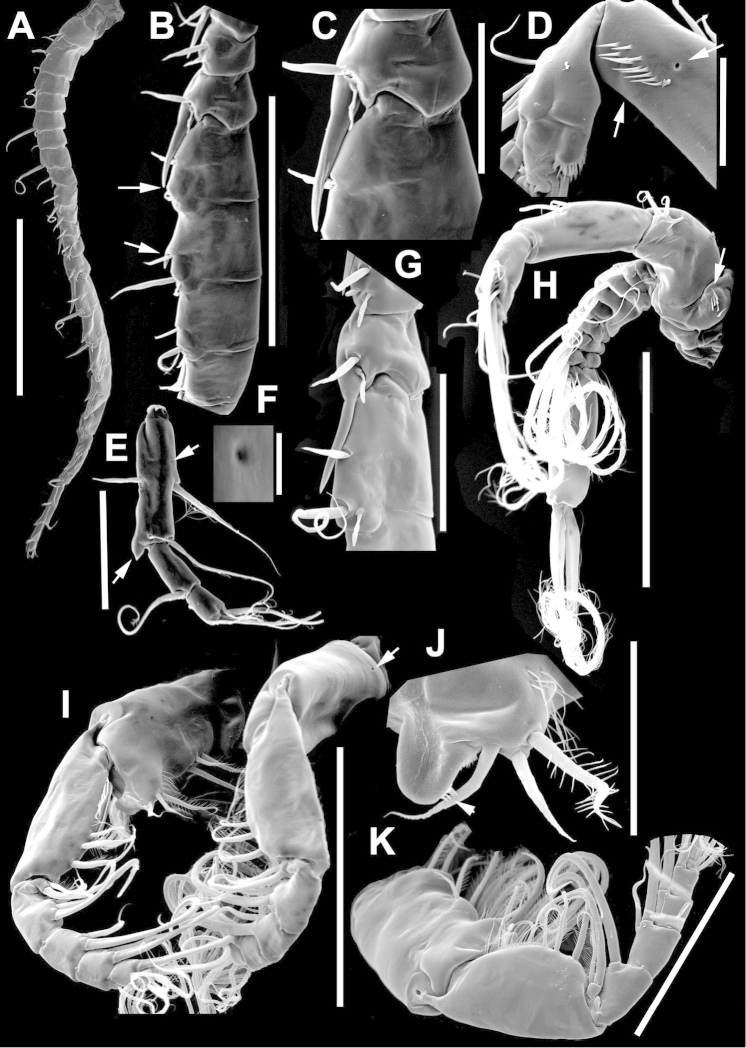
*Argyrodiaptomus azevedoi* male, SEM photographs. **A** Geniculate right antennule (500 µm) **B** Segments 12–17 of A1R (300 µm) **C** Segments 13–14 of A1R (100 µm) **D** Enp of A2, showing pore and spinular ornamentation (50 µm) **E** Segments 20–22 of A1R (100 µm) **F** Inset showing pore on segment 20 of A1R (5 µm) **G** Segments 12–16 of A1R (100 µm) **H** A2 (200 µm) **I** Maxillipeds (200 µm) **J** Distal endite of maxilliped (50 µm) **K** Maxilliped (200 µm).

**Figure 6.**
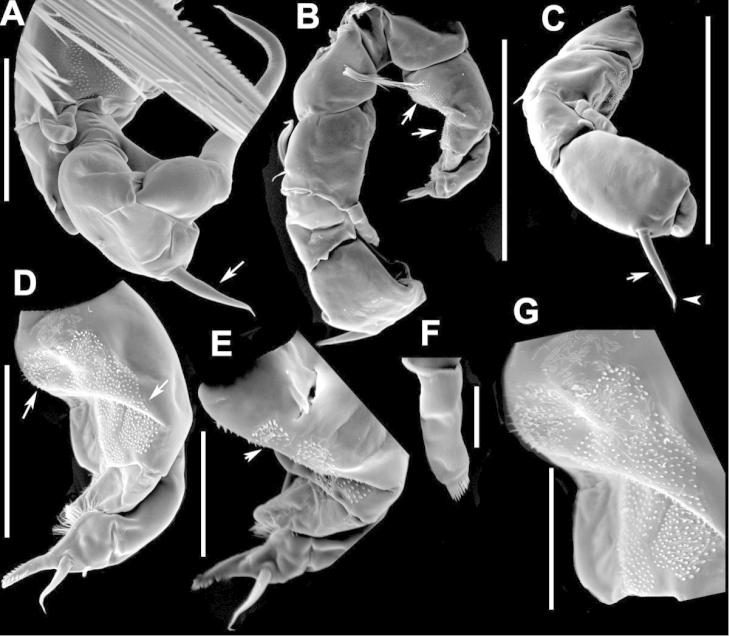
*Argyrodiaptomus azevedoi* male, SEM photographs. **A** P5R (100 µm) **B** P5L and P5R (200 µm) **C** P5R (200 µm) **D** P5L (100 µm) **E** P5L (50 µm) **F** EnpP5R (20 µm) **G** BspP5L, showing ornamentation of spinules (50 µm).

**Figure 7.**
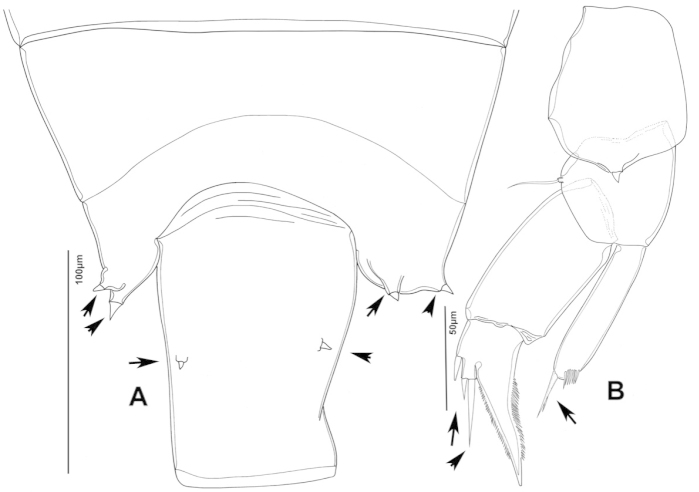
*Argyrodiaptomus azevedoi* female. **A** Dorsal view of Ped4 and Ped5 and GS **B** P5R.

**Figure 8.**
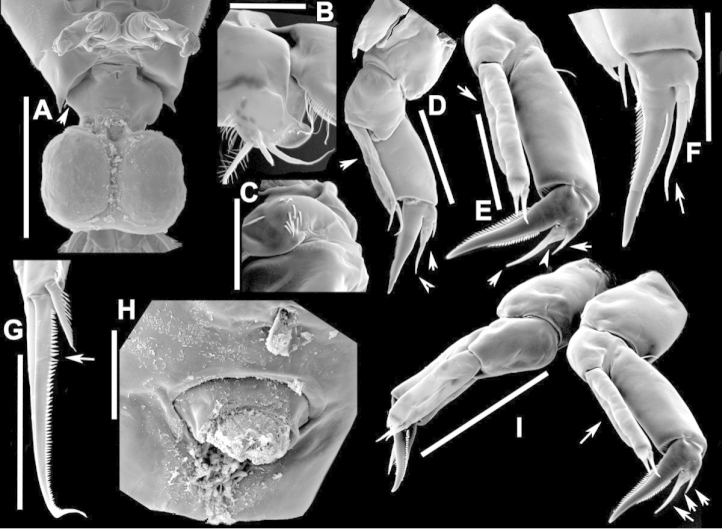
*Argyrodiaptomus azevedoi* female, SEM photographs. **A** Ventral view of fifth pediger bearing P5, and GS with eggs attached (200 µm) **B** Distal endite of maxilliped (50 µm) **C** Detail of spinules on basis of antenna (50 µm) **D** P5L (50 µm) **E** P5R (50 µm) **F** Distal part of ExpP5R (50 µm) **G** Apical spine of Enp3P4 (100 µm) **H** Genital area (fertilized female) on ventral surface of GS (50 µm) **I** P5 (100 µm).

**Figure 9.**
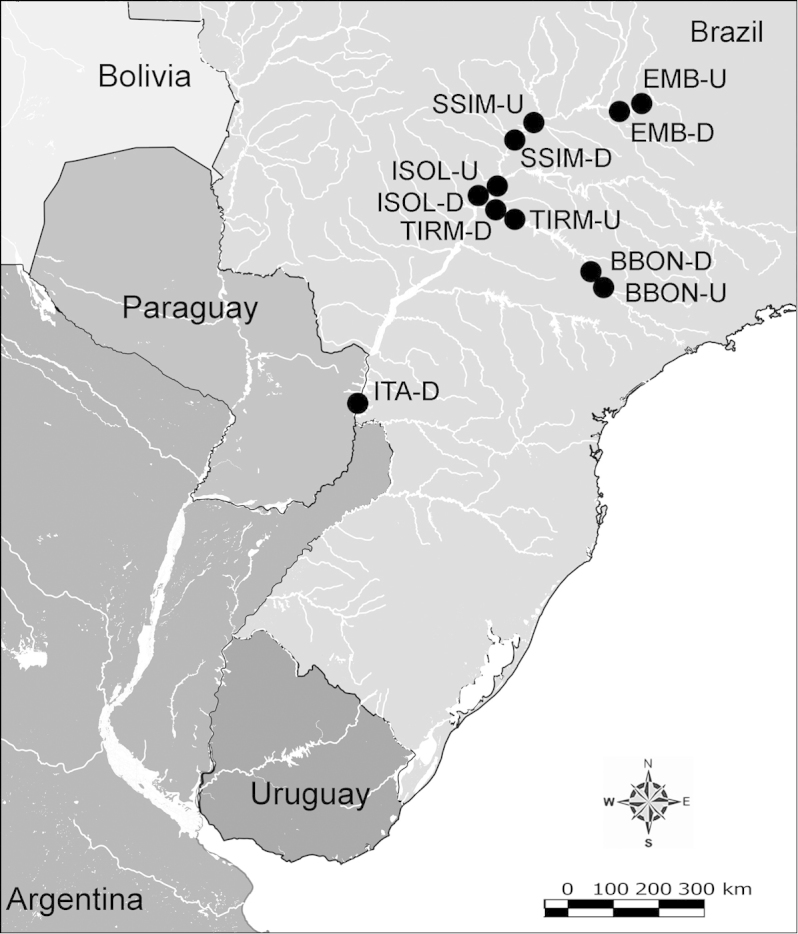
Geographical distribution of *Argyrodiaptomus azevedoi* in de la Plata river basin.

Figs 4 , 5 , 6 , 7 , 8 , 9

Diaptomus azevedoi Wright, 1935

###### Diagnosis.

**Adult male, body length (excluding caudal setae) 1704 µm.** Segment 11 of A1R with modified seta about twice length of modified seta on segment 10; segment 13 with long modified seta extending well beyond mid-point of segment 14 (Figs 4D, 5A–C, G); segment 20 with or without small distal process (cf. Figs 4J and 5E). First endopodal segment of A2 ornamented with pore and patch of spinules (Figs 4L, 5D). Inner margin of left and right BspP5 ornamented with several groups of small spinules (Figs 4E, F, I, K, 6D, E, G). Lateral spine moderately long, about 1/3 length of terminal claw, inserted at outer-distal angle (Figs 4H, 6C). Terminal claw weakly sinuously curved over most of its length, strongly recurved distally (Figs 4A–C, G, H, 6A). EnpP5 2-segmented (Fig. 6B, F).

**Adult female, body length (excluding caudal setae) 1851 µm.** Dorsal surface of Ped4 and Ped5 without spinule rows; complete suture present between Ped4 and Ped5; lateral wings slightly asymmetrical, with right wing larger than left; each wing with two sensillae close to postero-distal corner (Fig. 7A). GS asymmetrical, about 1.6 times longer than wide; anterior part weakly swollen, right side more swollen than left (Figs 7A, 8A, H). P5 symmetrical (Figs 7B, 8I), with small conical process at outer distal corner of Cx with strong triangular apical sensilla. BspP5 with short outer seta about 1/3 length of EnpP5. EnpP5 unisegmented, with longitudinal groove (Fig. 8D-E). Exp 3-segmented; lateral spine of Exp2 exceeding length of Exp3 (Fig. 8F); external seta on Exp3 about 1/3 length of internal seta (Fig. 7B); internal seta reaching 2/3 of terminal claw length (Fig. 8F).

###### Remarks.

In identifying species of Diaptomidae in general, caution must be exercised in using the shape of segment 20 of A1R for species identification because it is often variable within a population. This segment has a falciform process at the distal angle in several species (Paggi 1976) but, as Wright (1935) points out, in a sample of 25 males of *Argyrodiaptomus azevedoi*, 11 did not exhibit any process (Fig. 4J) while the remaining 14 carried a short process (Fig. 5E). There is an extensive ornamentation of spinule patches on the surface of the male BspP5 in this species.

Ultrafine-scale ornamentation characters might prove to be valuable in future comparative studies, including 1) the presence of a pore on segment 20 of male A1R (Fig. 5F), 2) the presence/absence of spinules of the basepodite of the A2B in both sexes (Figs 5H, 8C), 3), the presence of a pore on the outer surface of the syncoxa, and of spinules on the distal syncoxal endite of the maxilliped (Figs 5I–K, 8B), and 4) details of the spinulation on the terminal setae of ExpP4 (Fig. 8G). In the absence of comparable SEM-based data on other species, such data cannot be used for routine species discrimination at present.

This species is widely distributed, extending from northeastern Brazil to the Itaipu Reservoir at the end of the upper Paraná River in southern Brazil (Fig. 9). Typically, few individuals are found in any one sample, so this is not a common species. However, among *Argyrodiaptomus*, this species is rarely misidentified, given its large body size and distinctive P5.

##### Argyrodiaptomus denticulatus

Taxon classificationAnimaliaCalanoidaDiaptomidae

(Pesta, 1927)

**Figure 10.**
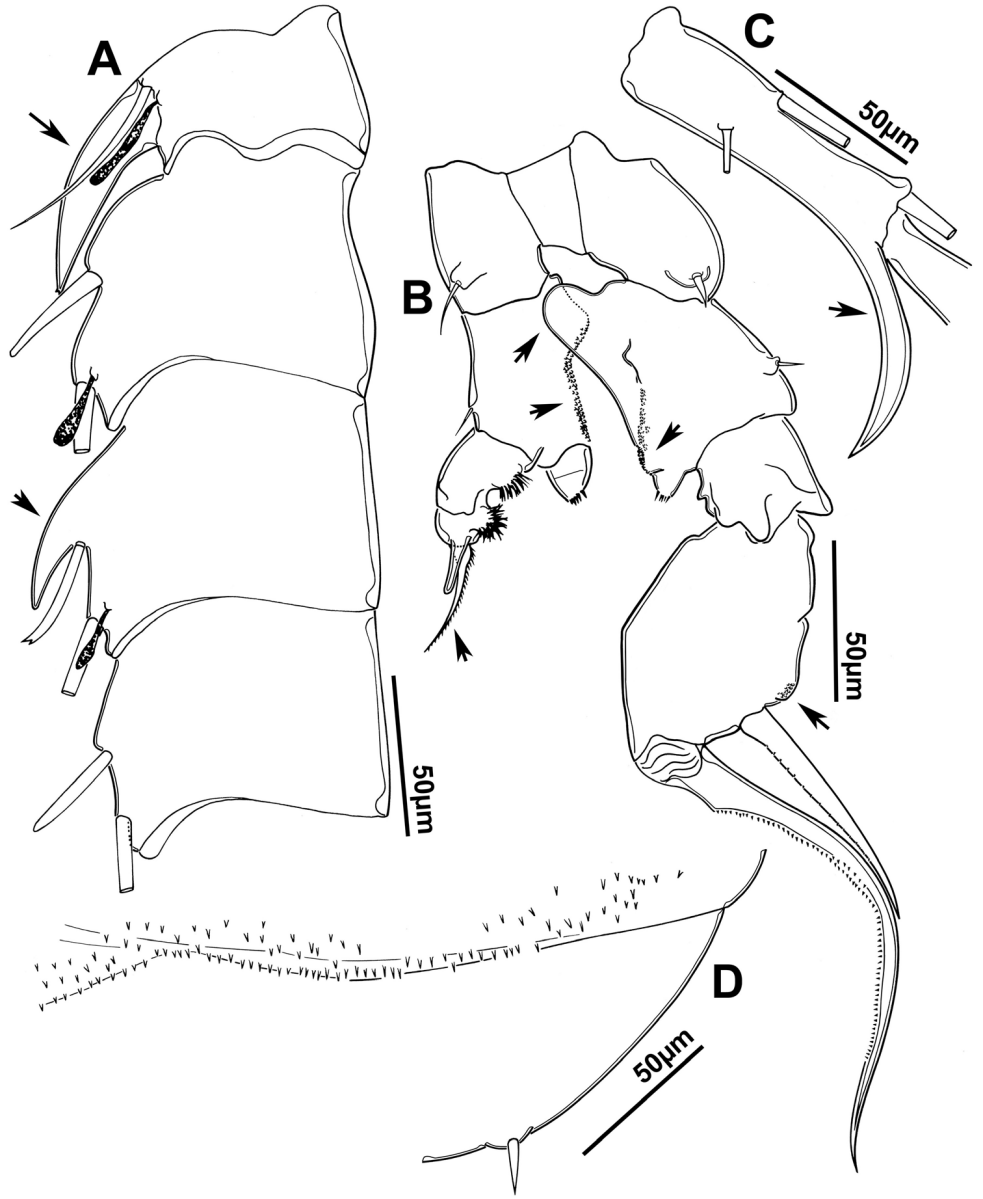
*Argyrodiaptomus denticulatus* male. **A** Segments 13–16 of A1R, showing spinous processes on segments 13 and 15 (arrowed) **B** Complete P5, showing proximal lobe on right Bsp overlapping margin of left Bsp **C** Segment 20 of A1R **D** Part of boundary between Ped4 and Ped5, showing ornamentation of dorsal spinules.

**Figure 11.**
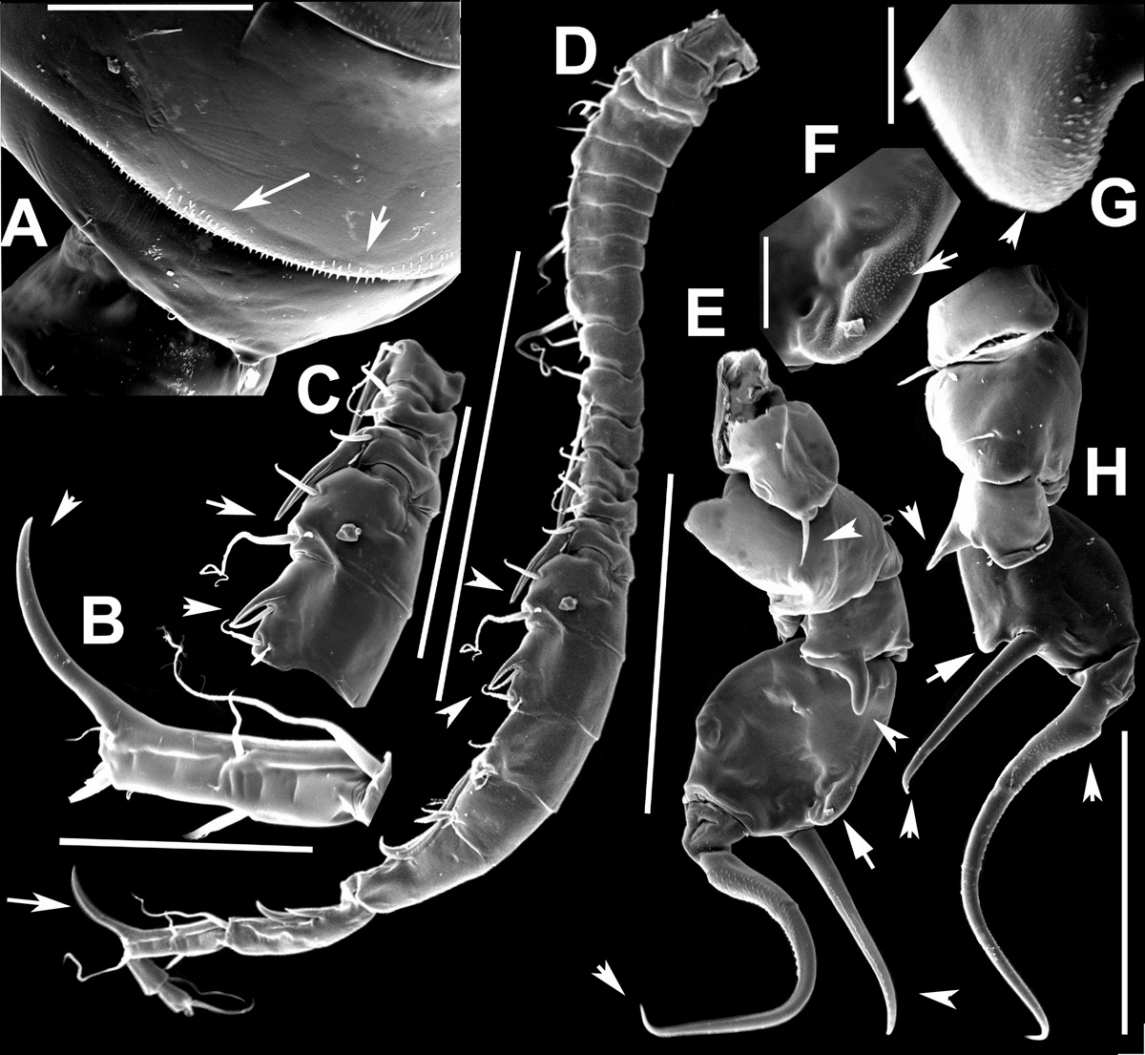
SEM photographs. *Argyrodiaptomus denticulatus* male. **A** Dorsolateral view of right side of Ped4 and Ped5 (100 µm) **B** Segment 20 of A1R (100 µm) **C** Segments 11–15 of A1R (200 µm) **D** A1R (500 µm) **E** P5R (200 µm) **F, G** Detail showing spinular ornamentation on process at base of lateral spine of Exp2P5R (**F** = 10 µm; **G** = 10 µm) **H** Right P5 (200 µm).

**Figure 12.**
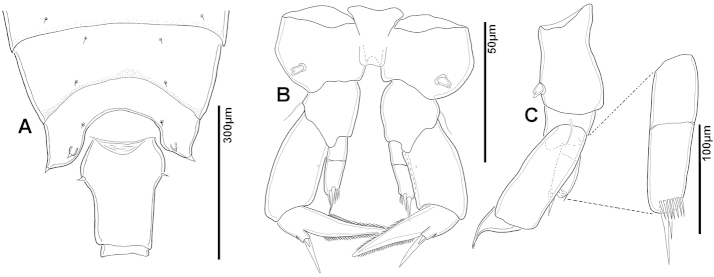
*Argyrodiaptomus denticulatus* female. **A** Dorsal view of posterior pedigers and GS **B** Complete P5 **C** Lateral view of left P5, with enlargement showing EnpP5.

**Figure 13.**
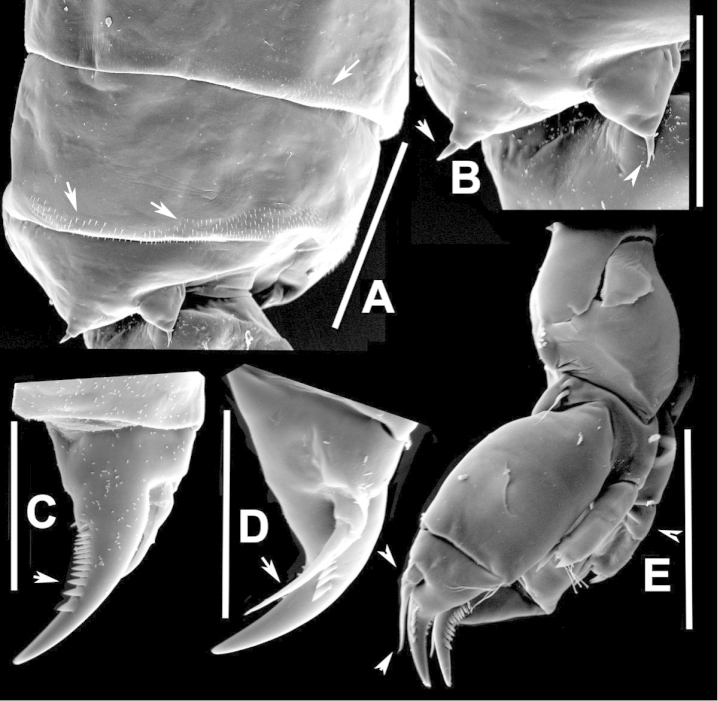
*Argyrodiaptomus denticulatus* female, SEM photographs. **A** Dorsolateral view of Ped4 and Ped5, showing ornamentation of spinules (200 µm) **B** Lateral wings on left side of Ped5, showing sensilla at apex of each projection (100 µm) **C** Left terminal claw (50 µm) **D** Right terminal claw (50 µm) **E** Lateral view of P5 (100 µm).

**Figure 14.**
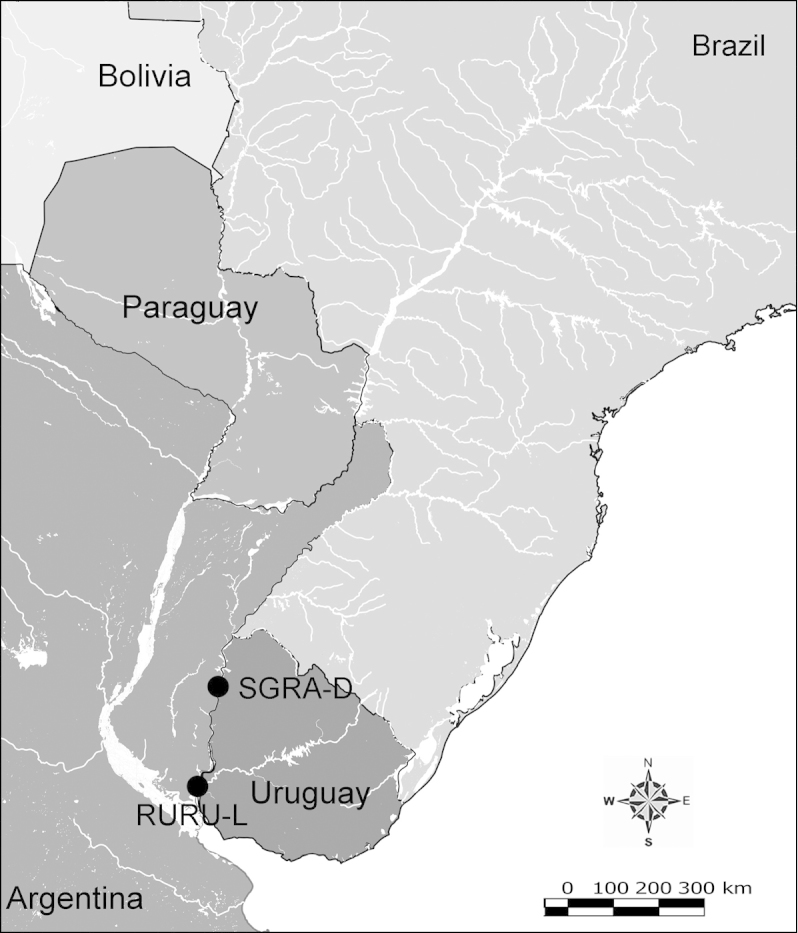
Geographical distribution of *Argyrodiaptomus denticulatus* in de la Plata river basin.

Figs 10 , 11 , 12 , 13 , 14

Diaptomus denticulatus Pesta, 1927

###### Diagnosis.

**Adult male, body length 1657 µm.** Dorsal surface of Ped5 ornamented with irregular rows of spinules (Figs 10D, 11A). Ur4 with rows of spinules on dorsal surface. Antennules (Fig. 11D) long, reaching beyond posterior margin of Ur2 but not exceeding CR. Modified seta of segment 13 of A1R with acute apex and reaching middle of segment 14 (Figs 10A, 11C); spinous process on anterior margin of segment 15 always present and well developed, slightly curved. Segment 20 typically with long, curved distal process (Figs 10C, 11B). Enp1A2 with row of spinules on dorsal margin and single pore. BspP5R with proximal expansion on internal margin, overlapping BspP5L (Fig. 10B); BspP5R ornamented with small spinules on surface of rounded distal process on inner margin; inner surface of BspP5L with spinules. Exp2P5 ornamented with minute spinules on process on outer margin proximal to insertion of lateral seta (Fig. 11E–H).

**Adult female, body length 1753 µm.** Spinules present dorsally along posterior margins of Ped3 and Ped4 (Fig. 13A). Complete suture present between Ped4 and Ped5. Lateral wings on Ped5 slightly asymmetrical, left wing longer than right; both wings projecting posteriorly, carrying two sensillae each, one at postero-distal corner (Fig. 13B). GS slightly asymmetrical, about twice as long as wide and slightly swollen anteriorly (Fig. 12A). P5 symmetrical (Fig. 12B), with small conical process at outer distal corner of Cx, bearing short triangular sensilla, bifid apically. BspP5 with short external seta, approximately one third length of Exp1P5. EnpP5 2-segmented, with two apical setae and distal row of spinules (Fig. 12B, C). ExpP5 3-segmented; lateral spine of Exp2P5 short, extending only to middle of Exp3P5; internal seta of Exp3P5 reaching beyond middle of terminal claw (Fig. 13E); external seta of Exp3P5 about 1/4 length of inner seta; terminal claw with two rows of 10–15 strong spinules (Fig. 13C–D).

###### Remarks.

The male illustrated was collected from the Salto Grande reservoir (SGRA-D), Uruguay River. The female illustrated here belongs to the collection of the Museo Argentino de Ciencias Naturales (Buenos Aires) (MACN-In 29733), and was examined because of the scarcity of females in the samples from de la Plata river basin. This species can be readily identified because of the distinctive structure of its P5 and the presence of dorsal rows of spinules on the male pedigers, which are lacking in other species of *Argyrodiaptomus*.

This species is common in the lower regions of the basins of the Paraná and Uruguay rivers (Figure 14), and has been reported in several studies, particularly by Argentinian researchers. Santos-Silva (2008) recorded this species in southern Brazil, in the state of Rio Grande do Sul. Previattelli et al. (2013) included a record of this species from Bolivia, a distance of about 1,300 km north of the previous northernmost record. It is possible that the distribution of this species extends beyond this northern limit to at least the line of latitude 35°, but verification is needed.

##### Argyrodiaptomus falcifer

Taxon classificationAnimaliaCalanoidaDiaptomidae

(Daday, 1905)

**Figure 15.**
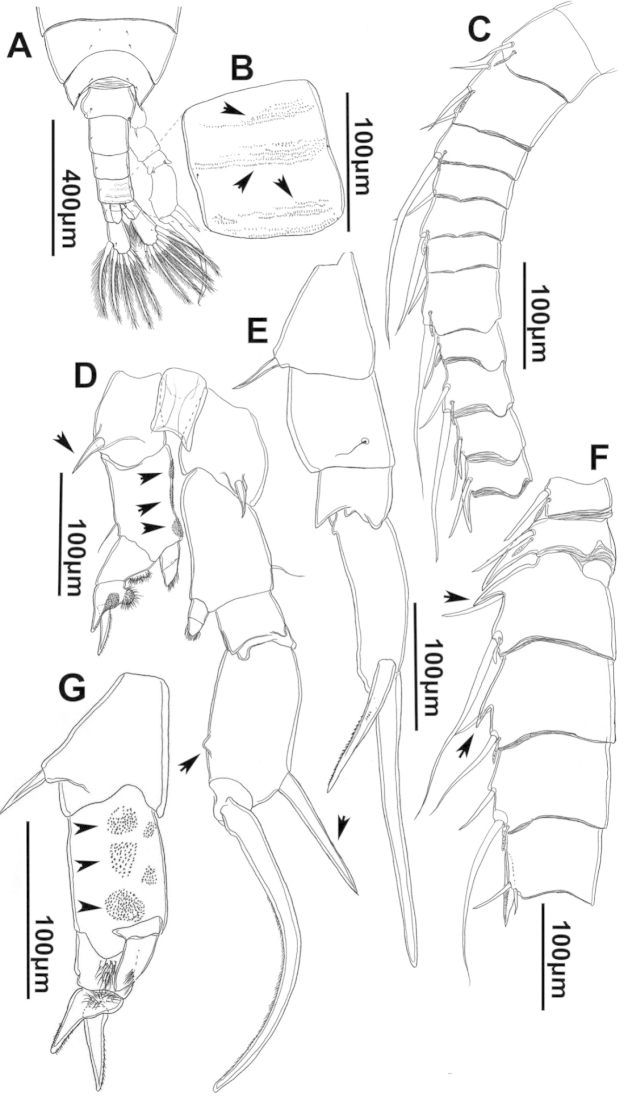
*Argyrodiaptomus falcifer* male. **A** Ped4, Ped5, and urosome somites **B** Details of ornamentation of spinules dorsally on Ur4 **C** Segments 1–11 of A1R **D** Complete P5 **E** P5R in caudal view **F** Segments 12–17 of A1R **G** P5L, showing spinular ornamentation on Bsp.

**Figure 16.**
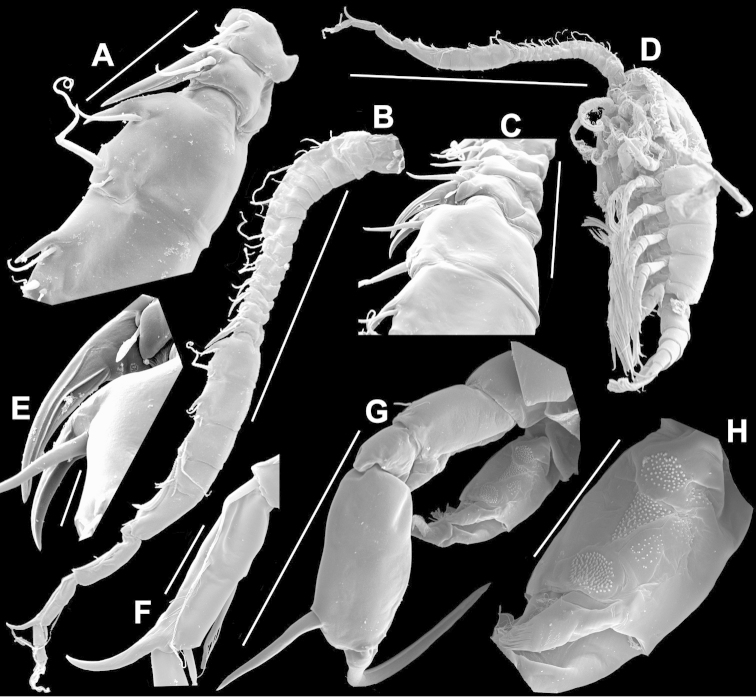
*Argyrodiaptomus falcifer* male, SEM photographs. **A** Segments 12–15 of A1R, showing detail of spinous process on segment 14 (arrowed) (100 µm) **B** Entire A1R (500 µm) **C** Segments 11–15 of A1R (100 µm) **D** Adult male, lateral view (1000 µm) **E** Spinous processes on segments 13 and 14 A1R (20 µm) **F** Segment 20 of A1R, showing falciform process (50 µm) **G** Complete P5, caudal view (200 µm) **H** Medial surface of BspP5L, showing spinular ornamentation (50 µm).

**Figure 17.**
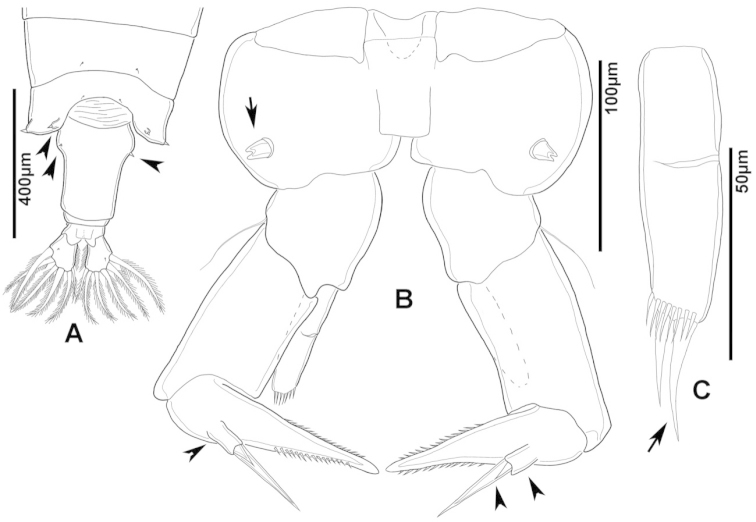
*Argyrodiaptomus falcifer* female. **A** Dorsal view of last two pedigers of prosome, GS and CR **B** P5 **C** EnpP5L.

**Figure 18.**
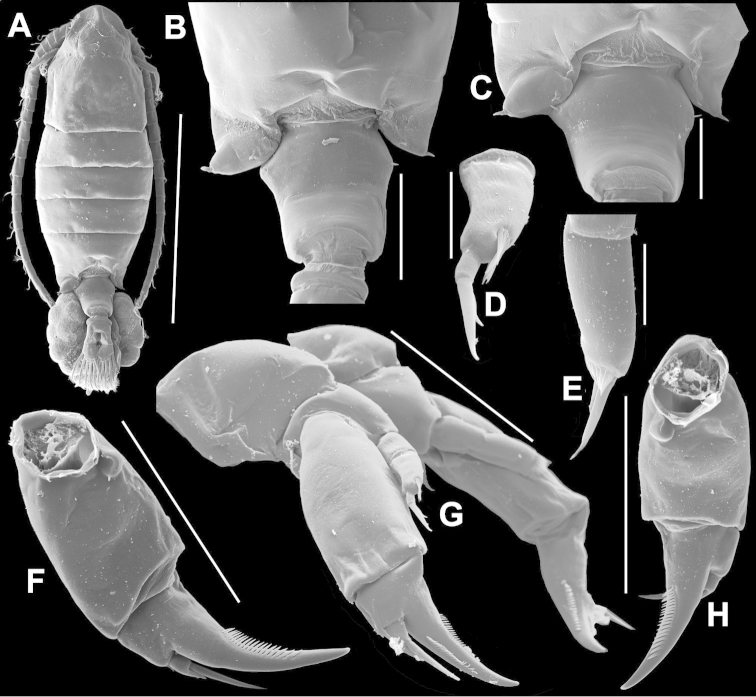
*Argyrodiaptomus falcifer* female, SEM photographs. **A** Dorsal view (1000 µm) **B** Ped4, Ped5, and GS (150 µm) **C** Ped4, Ped5, and GS **D** EnpP5R (20 µm) **E** EnpP5L (20 µm) **F** ExpP5L (100 µm) **G** P5 (100 µm) **H** ExpP5L (100 µm).

**Figure 19.**
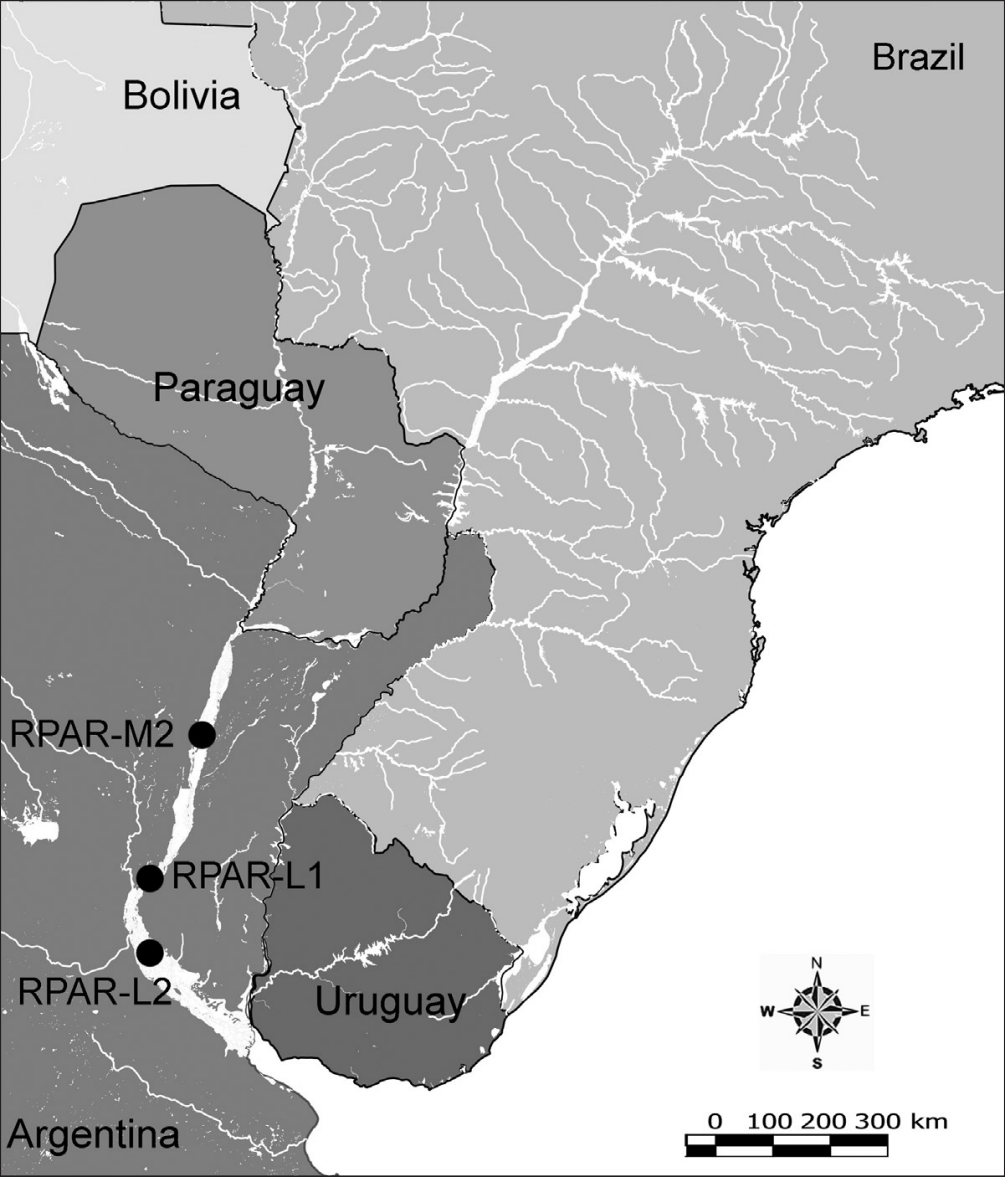
Geographical distribution of *Argyrodiaptomus falcifer* in de la Plata river basin.

Figs 15 , 16 , 17 , 18 , 19

Diaptomus falcifer Daday, 1905
Diaptomus argentinus Wright, 1938
Argyrodiaptomus argentinus (Wright, 1938)

###### Diagnosis.

**Adult male, body length 1495 µm.** Ur4 with patches of fine spinules on dorsal surface (Fig. 15A–B). Inner margin of CR with setules, outer margin smooth. Modified seta of segment 13 of A1R short, not reaching beyond tip of spinous process on segment 14; spinous process of segment 14 well developed (Fig. 16A, C, E); spinous process of segment 15 of A1R shorter than spinous process of segment 14 (Fig. 15F); segment 20 of A1R typically with falciform process; falciform process variable, when present can attain length up to half length of segment (Fig. 16B, D, F). Right BspP5 approximately 1.5 times longer than wide (Fig. 15D), ornamented with few spinules, lacking spinules in many specimens; left BspP5 approximately 2.5 times longer than wide, with 5 patches of spinules (Figs 15G, 16G, H). Lateral spine of right Exp2P5 about 40% of length of terminal claw (Fig. 15E).

**Adult female, body length 1648 µm.** Dorsal surface of pedigers lacking ornamentation of spinules (Fig. 18A–C). Complete suture present between Ped4 and Ped5; lateral wings slightly asymmetrical (Fig. 17A); both wings with two sensillae each, one at distal corner. GS weakly asymmetrical, approximately 1.5 times longer than wide (Fig. 18B). Anterior of GS slightly swollen. P5 symmetrical, with small conical process at outer distal corner of Cx, bearing short triangular sensilla with bifid apex (Fig. 17B). BspP5 with short outer seta, less than half length of outer margin of Exp1P5. EnpP5 2-segmented but with incompletely-expressed transverse articulation and longitudinal groove (Figs 17C, 18D, E). ExpP5 3-segmented; lateral spine of Exp2P5 reaching about to midlength of external margin of Exp3P5 (Fig. 18F); external seta of Exp3P5 about 3/4 length of internal seta; internal seta about half length of terminal claw (Fig. 18G–H).

###### Remarks.

The specimens illustrated here were caught in the middle stretch (RPAR-M2) of the Paraná River, and the sample contained only a few females and two males. Paggi (2006) provided a redescription and confirmed the validity of *Argyrodiaptomus falcifer*, recognizing at the same time that *Argyrodiaptomus argentinus* (Wright, 1938) should be treated as a junior subjective synonym of this species. Paggi also summarized its geographical distribution across Argentina and Paraguay. Previattelli (2006) also illustrated this species, but he considered this taxon under the binomen *Argyrodiaptomus argentinus*.

This species, together with others of this genus, can be very abundant in small scale habitats, such as water pools, and is generally scarce in lotic environments. It was found at only three stations in the present study (Fig. 19).

##### Argyrodiaptomus furcatus

Taxon classificationAnimaliaCalanoidaDiaptomidae

(Sars, 1901)

**Figure 20.**
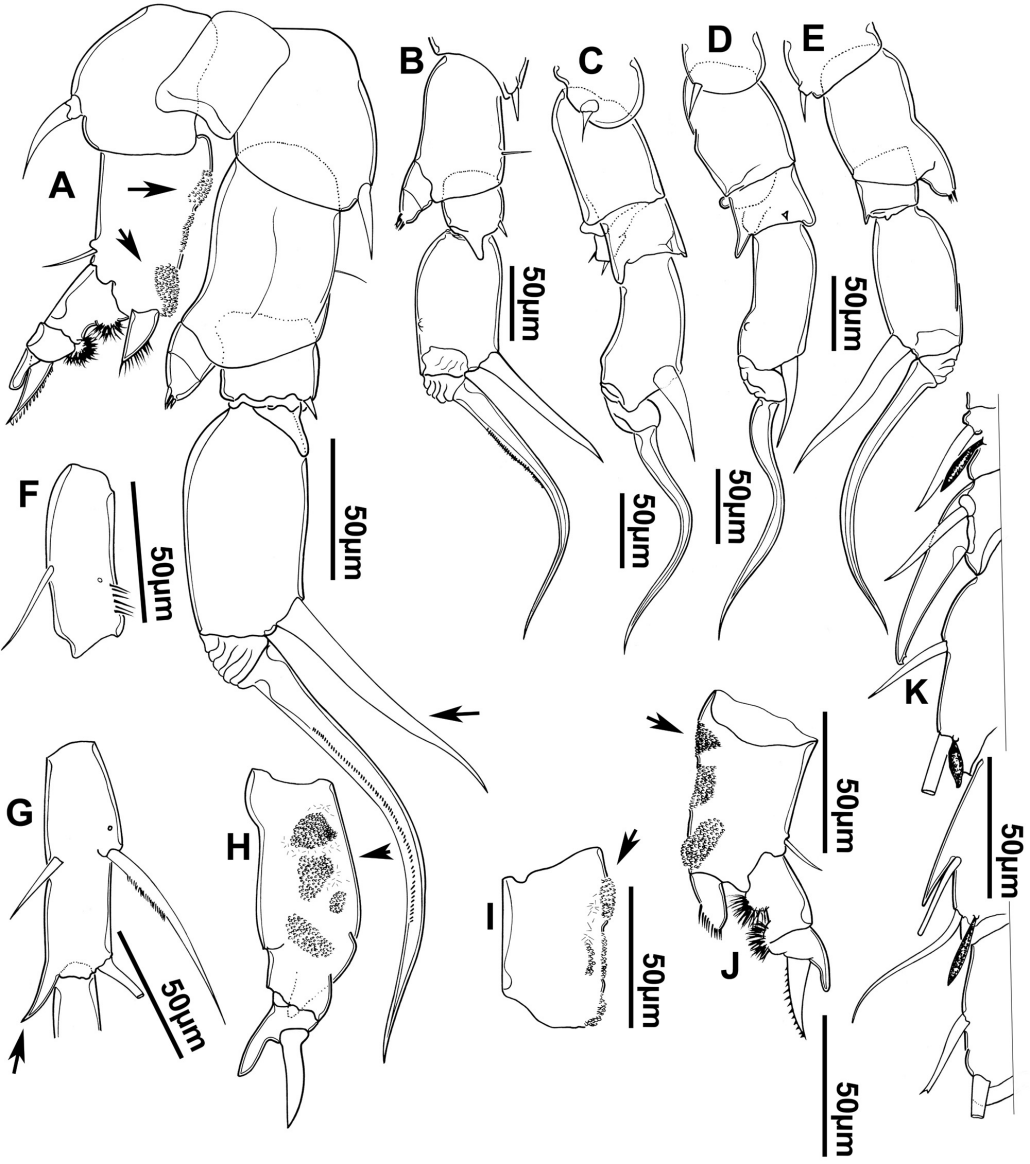
*Argyrodiaptomus furcatus* male. **A** P5; **B–E** Different views of P5R **F** Segment 1 of Enp of antenna 2 **G** Segment 20 of A1R; **H–J** Different views of P5L, showing details of ornamentation **K** Segments 12–16 of A1R.

**Figure 21.**
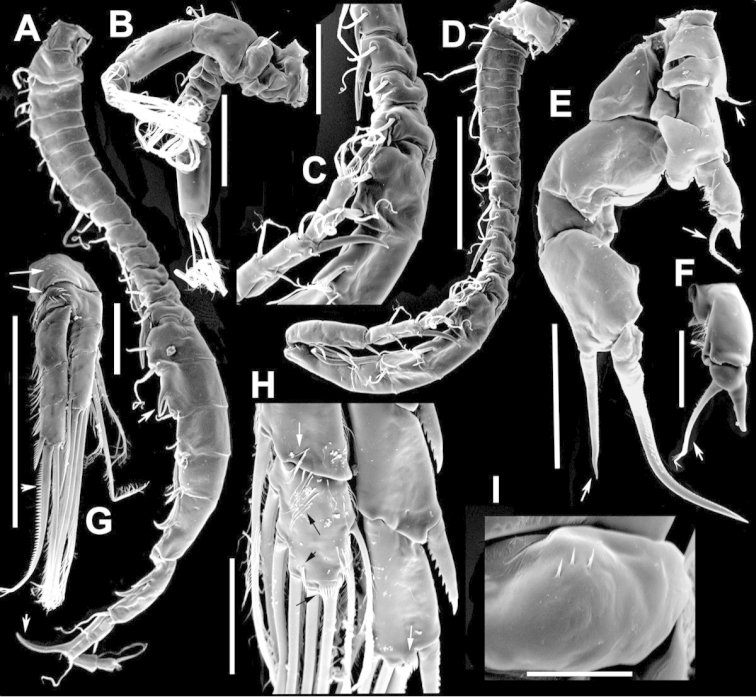
*Argyrodiaptomus furcatus* male, SEM photographs. **A** A1R (500 µm) **B** A2R (200 µm) **C** Segments 10–15 and 20–22 of A1R (200 µm) **D** A1R (500 µm) **E** P5 (300 µm) **F** Distal segments of Exp2P5L (50 µm) **G** Right P1 (200 µm), anterior view **H** Distal segments of right P4 rami (50 µm), posterior view **I** Basis of right A2 showing spinules (20 µm).

**Figure 22.**
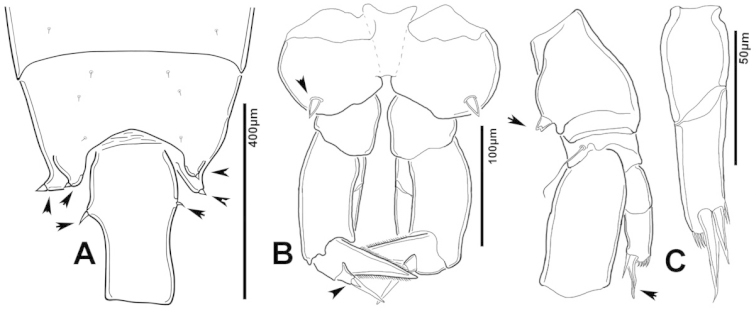
*Argyrodiaptomus furcatus* male. **A** Dorsal view of posterior pedigers and GS **B** P5 **C** Caudal view of left P5, with inset showing EnpP5.

**Figure 23.**
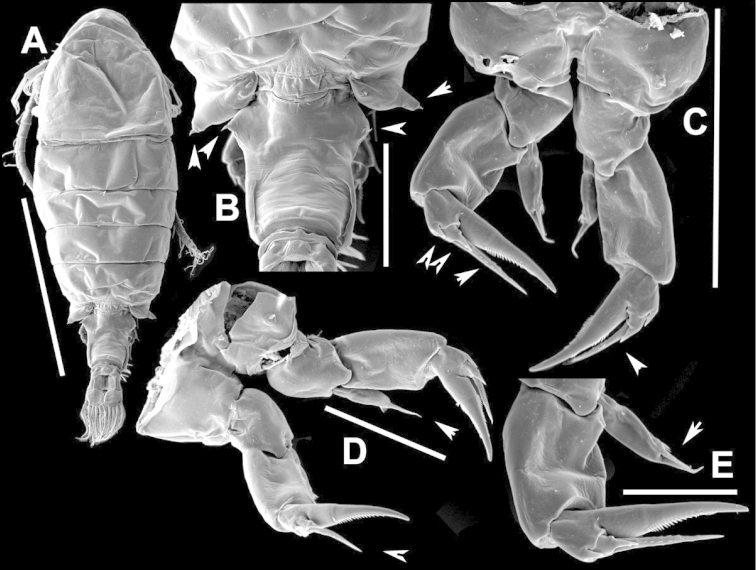
*Argyrodiaptomus furcatus* female, SEM photographs. **A** Dorsal view (1000 µm) **B** Ped4, Ped5, and GS (500 µm) **C** P5 (300 µm) **D** P5 (150 µm) **E** EnpP5R and ExpP5R (100 µm).

**Figure 24.**
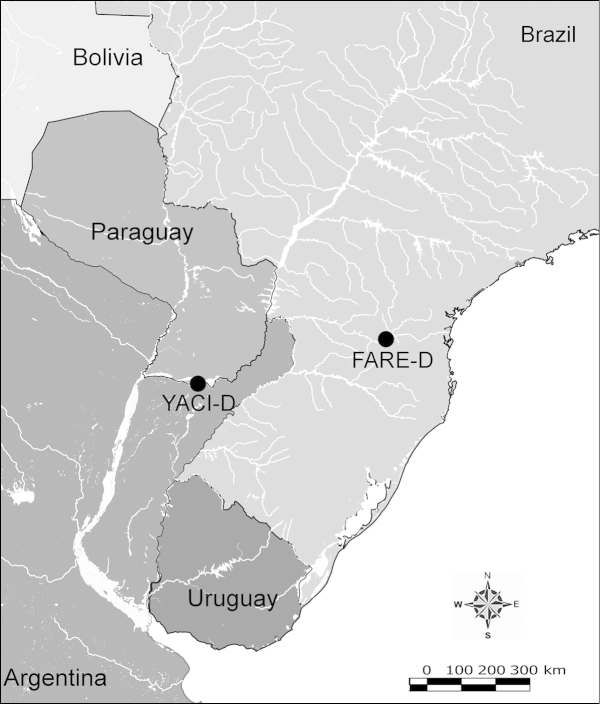
Geographical distribution of *Argyrodiaptomus furcatus* in de la Plata river basin.

Figs 20 , 21 , 22 , 23 , 24

Diaptomus furcatus Sars, 1901

###### Diagnosis.

**Adult male, body length 1354 µm.** Modified seta of segment 13 of A1R with bifid apex, reaching or exceeding proximal border of segment 14 (Fig. 20K); spinous process on anterior margin of segment 15 present, or sometimes absent; distal process of segment 20 sometimes absent, if present, variable from short (Fig. 20G) to strong, long and curved (Fig. 21A, C, D). Basipodite of A2 with spinular ornamentation (Fig. 21B, I); Enp1A2 with row of spinules and single pore (Fig. 20F). Left BspP5 ornamented with setules and patches of small spinules (Fig. 20H–J); left Exp2P5 with long lateral seta (Fig. 21F); right CxP5 with distal process, ornamented with fine spinules (Fig. 20D, E); right BspP5 with smooth surface, with simple distal process (Fig. 20B); lateral spine of right Exp2P5 slightly curved at apex, inserted posterodistally on segment very close to base of claw (Figs 20A–E, 21E), reaching about to middle of terminal claw; terminal claw curved and twisted, with torsion in three planes. Leg 1 with distinctive spinulation on coxa, basipodite and both rami (Fig. 21G, H).

**Adult female, body length 1712 µm.** Dorsal surface of Ped4 and Ped5 unornamented, lacking lines of spinules (Figs 22A, 23A). Incomplete suture present dorsally between Ped4 and Ped5; lateral wings slightly asymmetrical, left side larger than right (Fig. 23B); both wings with pair of sensillae, located at distal corner. GS asymmetrical, about twice as long as wide; slightly swollen anteriorly, more on left side than on right (Fig. 22A). P5 symmetrical, with small conical process at outer distal corner of CxP5 bearing short, triangular sensilla (Fig. 22B). BspP5 with short outer seta (Fig. 22C), measuring about 70–80% of external margin of EnpP5. EndP5 2-segmented, although suture only visible from certain angles (Figs 22C, 23C–E). ExpP5 3-segmented; lateral spine of Exp2P5 short, not exceeding length of external margin of Exp3P5; external seta of Exp3P5 about ¼ (25%) length of internal seta; internal seta about 2/3 length of terminal claw (Fig. 23E).

###### Remarks.

The figured specimens were collected from the Iguaçu River at the reservoir of Foz do Areia (FARE-D) (Figure 24). This species was originally described from material collected in the state of São Paulo and, according to Wright (1927), occurs in Brazil (south and southeastern, and possibly also in the mid-west) and in Argentina. Santos-Silva (2008) and Previattelli et al. (2013) cited several records of *Argyrodiaptomus furcatus* from south and southeastern Brazil. Together these studies confirm the occurrence of this species mainly in reservoirs of southeastern and southern Brazil, and in northern Argentina. Along with other large species of this genus, this species can co-occur with *Argyrodiaptomus azevedoi* (in southeastern Brazil) and with *Notodiaptomus spiniger* (Brian, 1925) (in southern Brazil and northern Argentina). *Argyrodiaptomus furcatus* tends to be closer to *Notodiaptomus spiniger* in body size, whereas *Argyrodiaptomus azevedoi* is larger.

#### Genus *Notodiaptomus* Kiefer, 1936

##### Notodiaptomus anisitsi

Taxon classificationAnimaliaCalanoidaDiaptomidae

(Daday, 1905)

**Figure 25.**
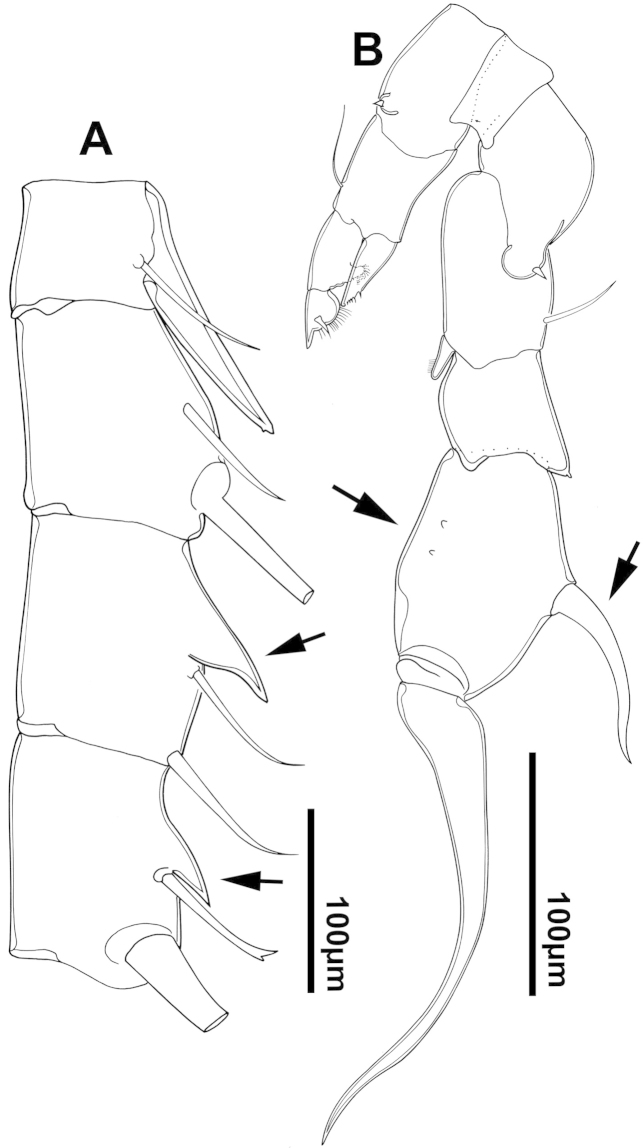
*Notodiaptomus anisitsi* male. **A** Segments 13–16 of A1R **B** P5.

**Figure 26.**
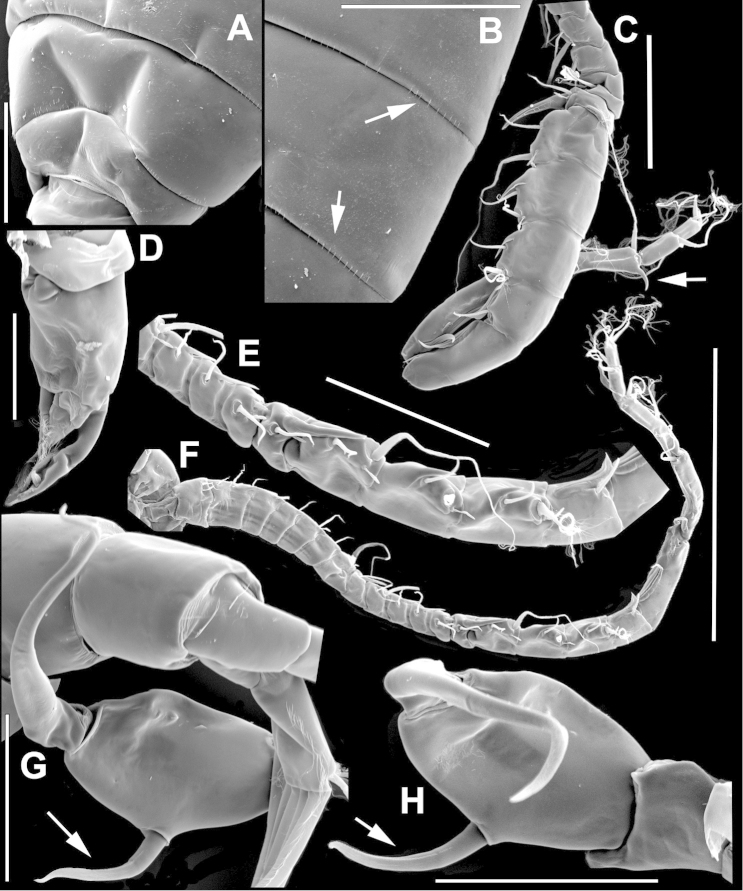
*Notodiaptomus anisitsi* male, SEM photographs. **A** Ped3, Ped4, and Ped5 (100 µm) **B** Details of ornamentation on Ped3, Ped4 and Ped5 (100 µm) **C** Segments 10–22 of A1R (300 µm) **D** P5L (500 µm) **E** Segments 9–18 of A1R (200 µm) **F** A1R (500 µm) **G** Terminal somites of urosome and Exp2P5R (100 µm) **H** ExpP5R (100 µm).

**Figure 27.**
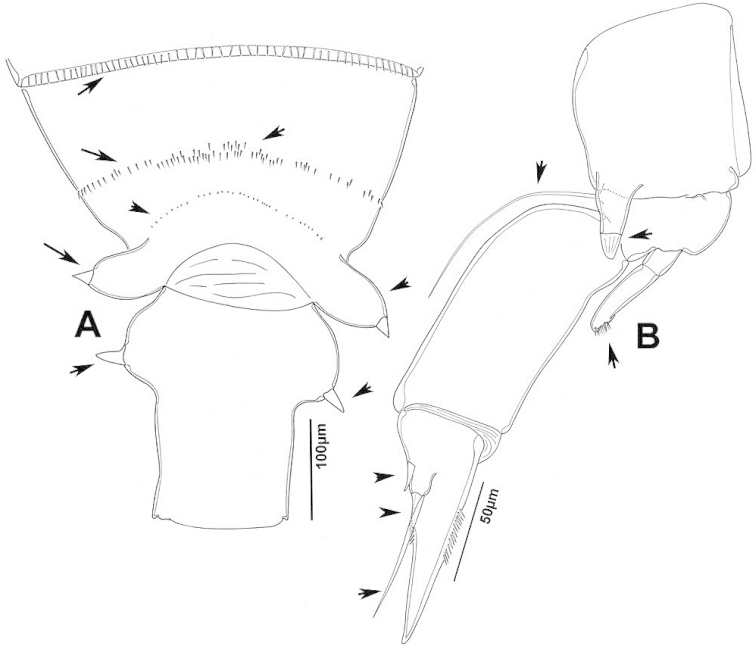
*Notodiaptomus anisitsi* female. **A** Dorsal view of Ped4 and Ped5, showing spinular ornamentation on dorsal surface, and GS **B** P5R.

**Figure 28.**
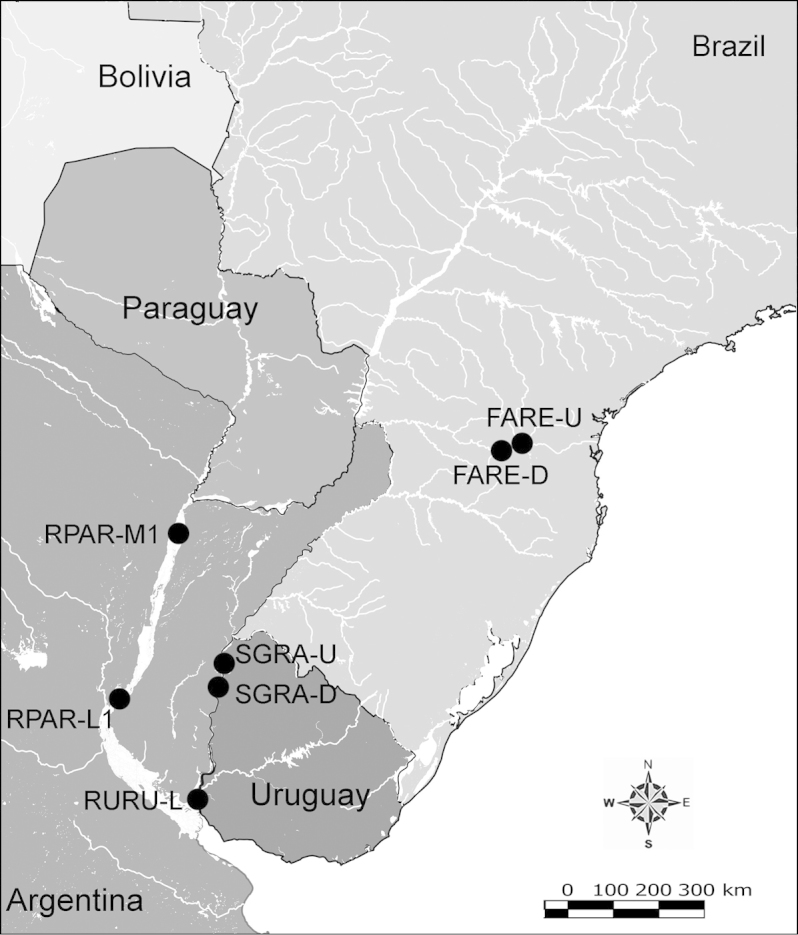
Geographical distribution of *Notodiaptomus anisitsi* in de la Plata river basin.

Figs 25 , 26 , 27 , 28

Diaptomus anisitsi Daday, 1905
Diaptomus inflexus Brian, 1925

###### Diagnosis.

**Adult male, body length 1144 µm.** Row of spinules present along posterior margins of Ped3 and Ped4 (Fig. 26A, B), Ped4 and Ped5 completely fused; A1R with well-developed spinous process on segments 15 and 16 (Figs 25A, 26C, E, F); segment 20 often with short curved distal process (Fig. 26C). Right Exp2P5 with two small nodules on surface (Fig. 25B); and bearing curved lateral spine inserted in middle of segment margin (Fig. 26G, H), and nearly half as long as terminal claw.

**Adult female, body length 1309 µm.** Single row of spinules present along posterior margin of Ped3; irregular rows of spinules present, marking plane of posterior margin of Ped4; discrete transverse row of minute spinules present across middle of Ped5 (Fig. 27A). Lateral wings asymmetrical, similar in size but left wing located more anteriorly and right wing positioned and directed more posteriorly; both lateral wings with single sensilla at apex (Fig. 27A). GS nearly symmetrical, no more than 20% longer than wide; dilated anteriorly with left and right swellings of similar size, left sensilla on apex of swelling and aligned perpendicular to longitudinal axis of body; right sensilla directed postero-laterally. P5 symmetrical (Fig. 27B) with conical process at outer distal corner of CxP5 bearing strong triangular sensilla at apex. BspP5 with long outer seta, extending beyond middle of outer margin of Exp1P5. EnpP5 2-segmented, not reaching middle of inner margin of Exp1P5. ExpP5 3-segmented; lateral spine of Exp2P5 about as long as outer margin of Exp3P5; external seta of Exp3P5 less than 1/4 (25%) length of internal seta; internal seta almost reaching tip of terminal claw.

###### Remarks.

The specimens illustrated were collected from the lower Paraná River (RPAR-L2). This species appears to be widely distributed in the south of the basin, from the Iguaçu River into more temperate conditions (Fig. 28).

Paggi (2001) provided good illustrations of this species and, in the same work, described a new species, *Notodiaptomus dentatus* Paggi, 2001, resembling and potentially confused with *Notodiaptomus anisitsi*. These two species deserve further investigation, as they may be part of a larger complex of species with very similar morphology.

##### Notodiaptomus carteri

Taxon classificationAnimaliaCalanoidaDiaptomidae

(Lowndes, 1934)

Figs 29 , 30 , 31

Diaptomus carteri Lowndes, 1934

###### Diagnosis.

**Figure 29.**
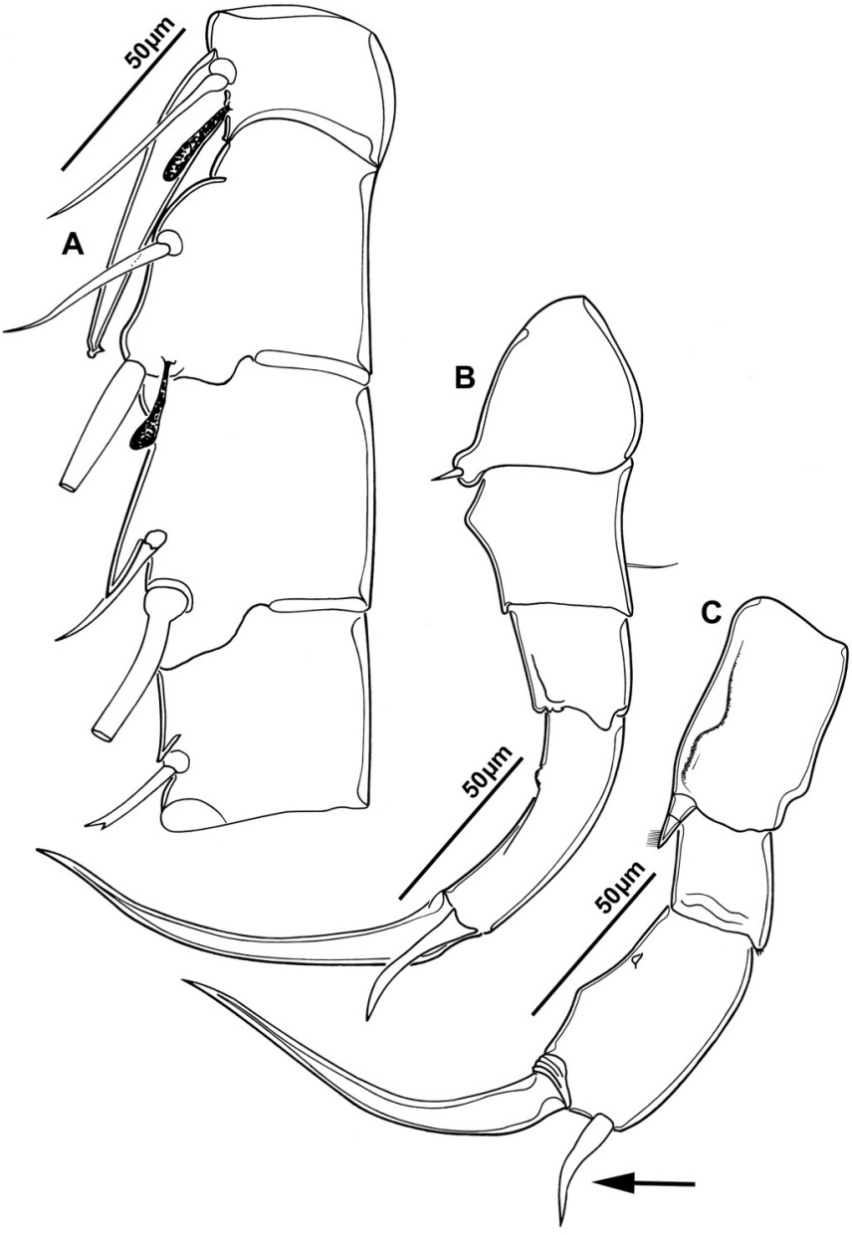
*Notodiaptomus carteri* male. **A** Segments 13–16 of A1R **B, C** P5R, different views.

**Adult male, body length 1484 µm.** Modified seta on segment 13 of A1R reaching distal end of segment 14 (Fig. 29A). Right BspP5 with longitudinal groove in surface, ornamented with small surface granulations (Fig. 29C). Right Exp2P5 with slightly outwardly curved lateral spine inserted on distal margin of segment, length about 1/3 of terminal claw; insertion of lateral spine separated from base of terminal claw by gap barely greater than basal width of spine (Fig. 29B, C).

**Figure 30.**
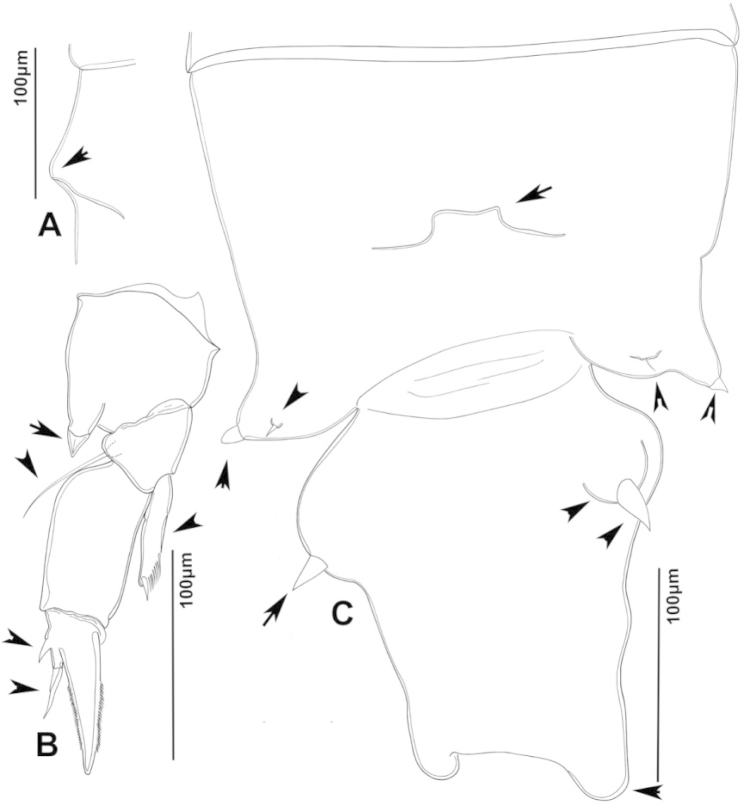
*Notodiaptomus carteri* female. **A** Lateral view of dorsal process on prosome located near suture incomplete between Ped4 and Ped5 **B** P5R **C** Posterior pedigers and GS.

**Adult female, body length 1770 µm.** Ped4 and Ped5 separated by incomplete suture; process present on mid-dorsal surface of Ped4 (Fig. 30A, C); lateral wings slightly asymmetrical, with two pairs of sensillae on each side; internal sensilla of right side thin, setule-like; left sensilla slightly larger than right. GS asymmetrical, about 1.5 times longer than wide, swollen anteriorly (Fig. 30C), swelling on left side greater than that on right margin; left side swelling hemispherical, bearing single posterolaterally-directed sensilla; swelling on right side with projecting semi-circular lobe with posteriorly-directed sensilla. Right distal margin of GS longer than left, each side with rounded process at posterior end. P5 symmetrical (Fig. 30B) with small conical process at distal corner of Cx bearing short and triangular sensilla, about as long as wide. BspP5 with outer seta barely reaching middle of outer margin of Exp1P5. EnpP5 one-segmented, reaching 2/3 distance along inner margin of Exp1P5. ExpP5 3-segmented; lateral spine of Exp2P5 short, about as long as outer margin of Exp3P5; external seta of Exp3 about half length of internal seta; internal seta reaching middle of terminal claw.

###### Remarks.

**Figure 31.**
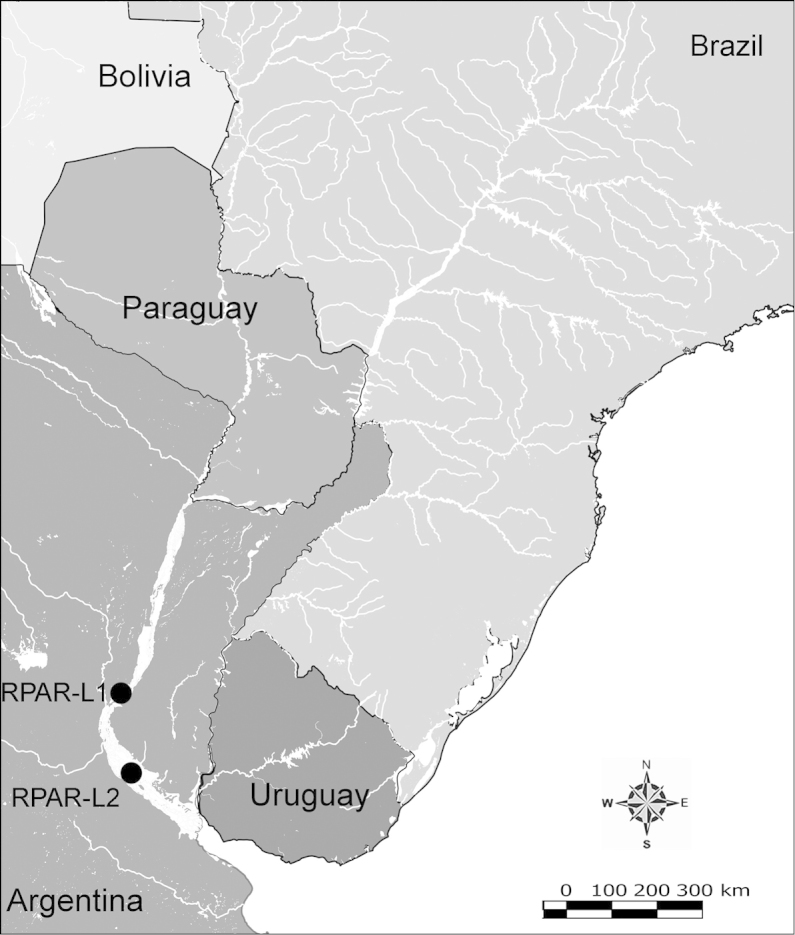
Geographical distribution of *Notodiaptomus carteri* in de la Plata river basin.

The illustrated specimens were collected from the lower Paraná River (RPAR-L2). The body length is slightly larger than the known range for males, 1315 to 1439 μm, given by Ringuelet and Martínez de Ferrato (1967). These authors published the first record from Argentina, near Santa Fe (equivalent to site RPAR-M3 in this study, Fig. 1), and they associated this species with the presence of floating aquatic macrophytes. The original description of this species (Lowndes 1934) was based on material from Paraguay, collected in flooded regions and wetlands, typically where there are macrophytes. In the present study the records of this species (Fig. 31) were about 500 km downstream from the locality sampled by Ringuelet and Martínez de Ferrato (1967). Santos-Silva (2008) provided records of this species from Paraguay, Argentina and southern Brazil, in the Patos Lagoon. Ringuelet and Martínez de Ferrato (1967) provided physical and chemical data for the water in the ponds where they found this species. Given the range of sites reported in this study, we infer that this species can occur in a wide range of habitat types.

Females of *Notodiaptomus carteri* can be readily distinguished from congeners by the asymmetrical swellings of the genital double-somite.

##### Notodiaptomus cearensis

Taxon classificationAnimaliaCalanoidaDiaptomidae

(Wright, 1936)

Figs 32 , 33 , 34

Diaptomus cearensis Wright, 1936

###### Diagnosis.

**Figure 32.**
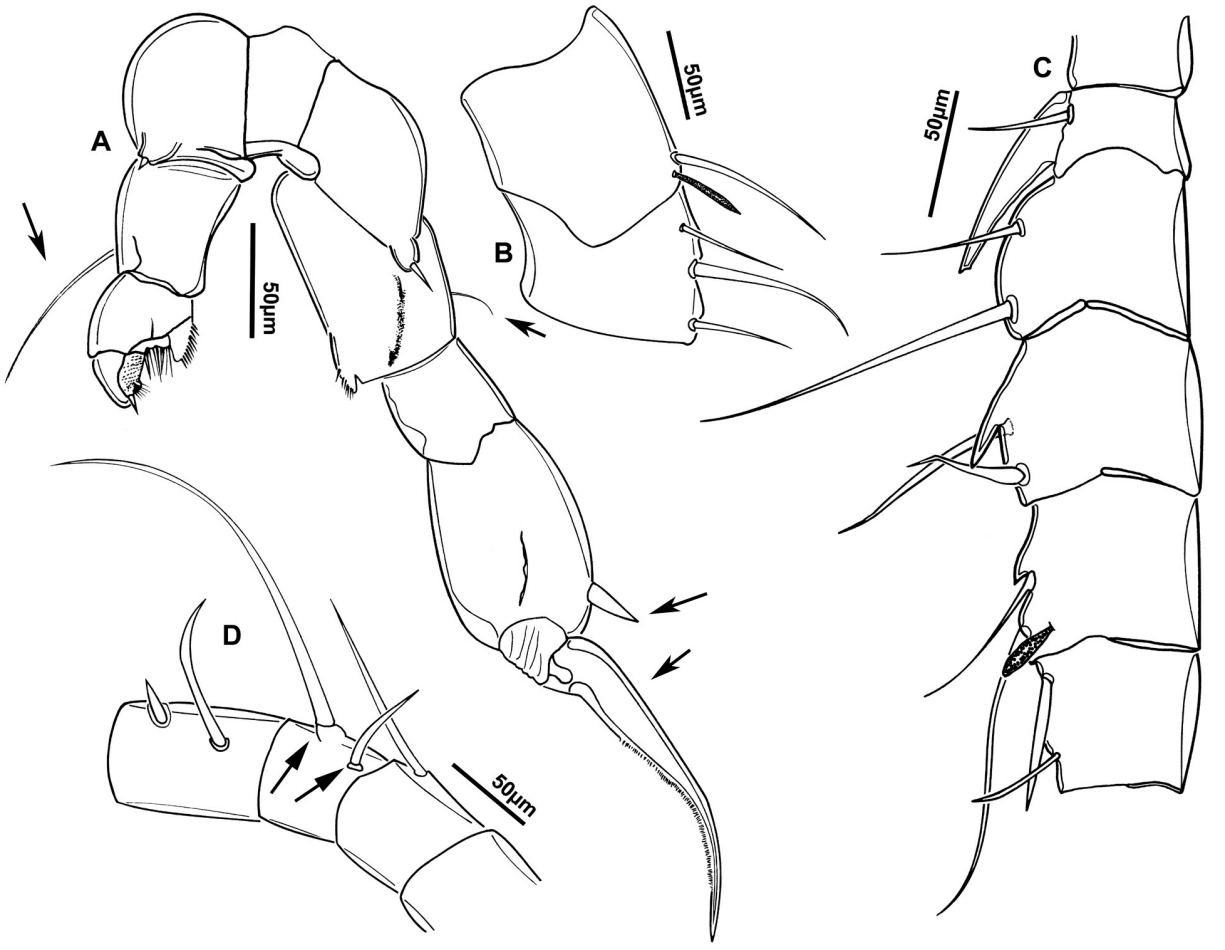
*Notodiaptomus cearensis* male. **A** P5 **B** Segments 1 and 2 of A1R **C** Segments 13–17 of A1R **D** Segments 12–10 of A1L.

**Adult male, body length 1008 µm.** Ped4 lacking row of spinules along posterior margin. First segment of A1R unornamented (lacking typical spinule row) (Fig. 32B); modified seta of A1R on segment 13 forming strong process with bifid apex and reaching middle of segment 14 (Fig. 32C). Segment 11 of A1L with 2 setae (Fig. 32D). Right BspP5 slightly longer than wide with longitudinal groove ornamented with small surface granules (Fig. 32A); longitudinal groove also present on Exp2P5; lateral spine of Exp2P5 short and straight, length approximately 1/6 (16%) of length of terminal claw, inserted sub-terminally.

**Figure 33.**
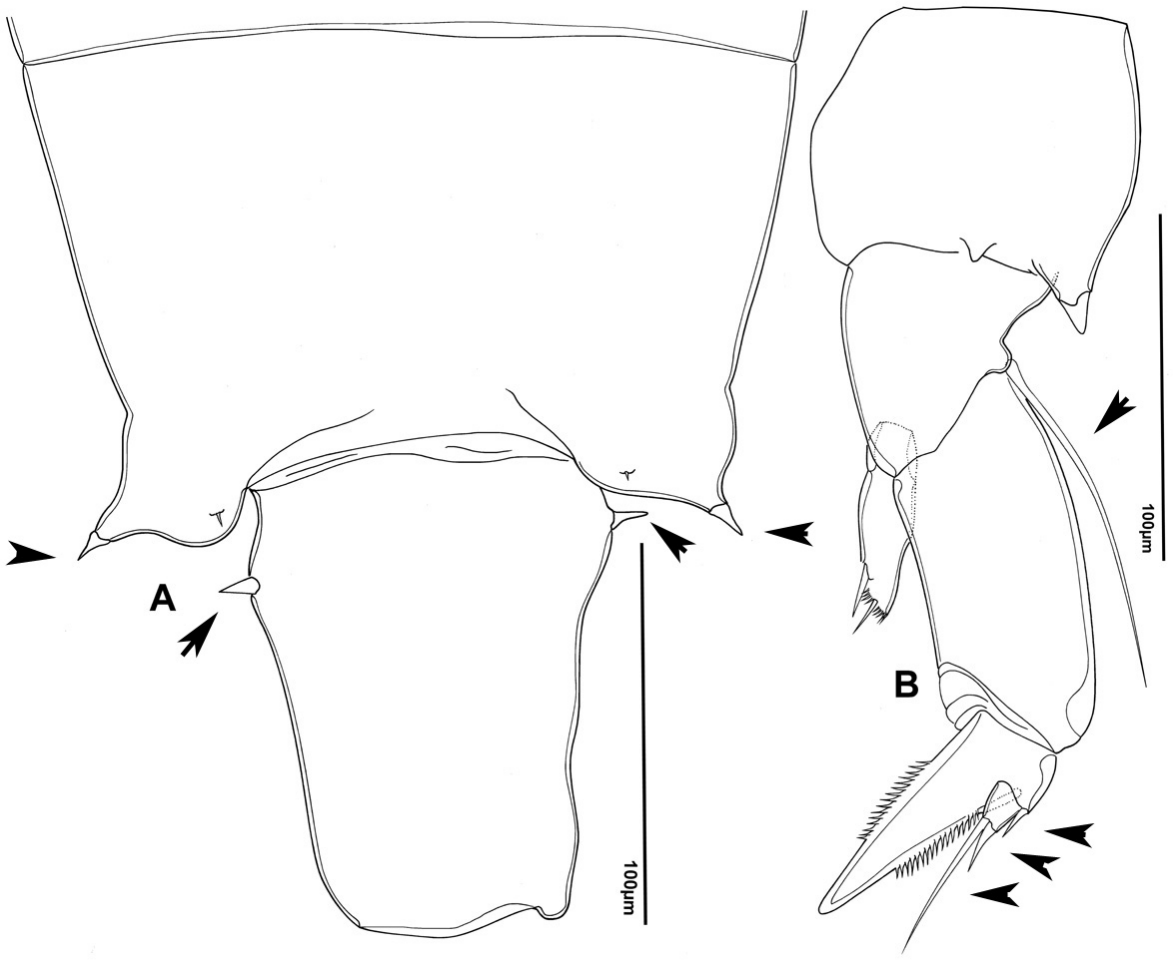
*Notodiaptomus cearensis* female. **A** Dorsal view of posterior pedigers and GS **B** P5.

**Adult female, body length 1485 µm.** Ped4 and Ped5 fused, without trace of suture; lateral wings symmetrical, both with two pairs of sensillae (Fig. 33A), one at distal tip, the other close to inner margin of wing. GS almost symmetrical, 1.3 times longer than wide; slightly dilated anteriorly, with swelling on right side slightly more marked than on left, both swellings with apical sensilla; left sensilla aligned perpendicular to longitudinal axis of body, right sensilla directed slightly anteriorly. P5 symmetrical (Fig. 33B), with short conical process at distal corner of Cx bearing triangular sensilla, longer than wide; BspP5 with long outer seta, almost reaching distal margin of Exp1P5; EnpP5 2-segmented, about as long as internal margin of Exp1P5. Exp 3-segmented; lateral spine of Exp2P5 similar in length to external margin of Exp3P5; external seta of Exp3 1/3 as long as internal seta; internal seta almost reaching apex of terminal claw.

###### Remarks.

**Figure 34.**
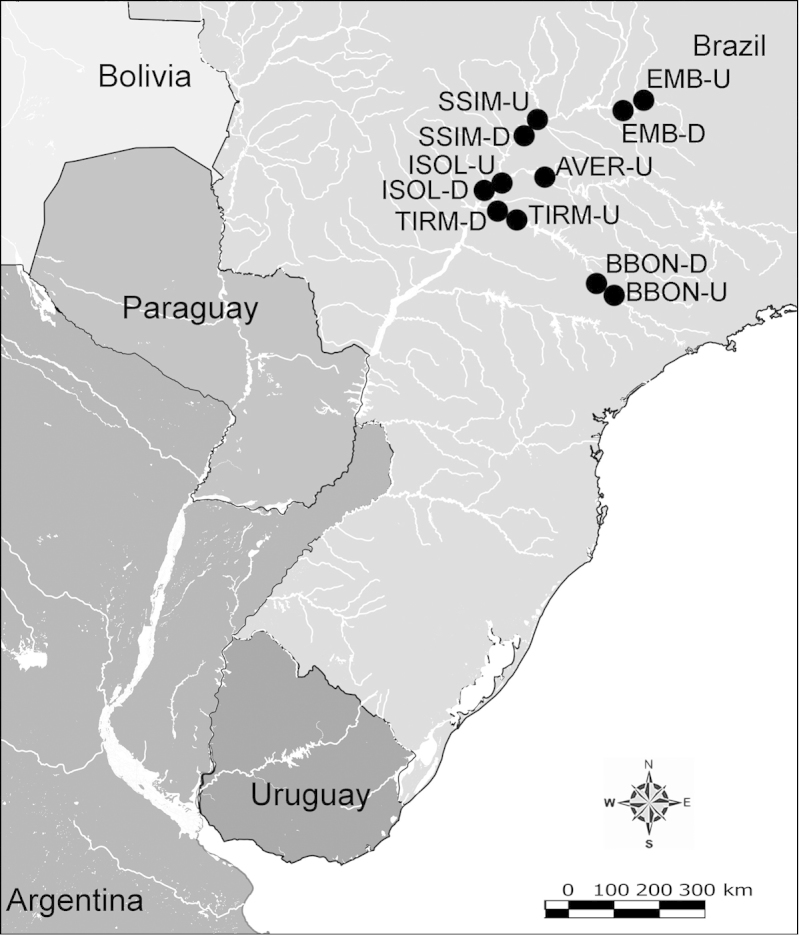
Geographical distribution of *Notodiaptomus cearensis* in de la Plata river basin.

The illustrated specimens were collected from the Parnaíba River at Emborcação Reservoir (Fig. 34), where this species co-occurred with at least two other diaptomids: *Argyrodiaptomus azevedoi* and *Notodiaptomus iheringi* (Wright, 1935). This species has been widely reported from across the north and northeast of Brazil and in Venezuela. In de la Plata river basin, Tundisi and Matsumura-Tundisi (1994) found this species in Barra Bonita Reservoir. This species can be confused with *Notodiaptomus iheringi*, but differs in the setal formula of some segments of the A1, in the absence of a row of spinules on the first segment of A1R, and in having a smaller body than *Notodiaptomus iheringi*.

##### Notodiaptomus conifer

Taxon classificationAnimaliaCalanoidaDiaptomidae

(Sars, 1901)

**Figure 35.**
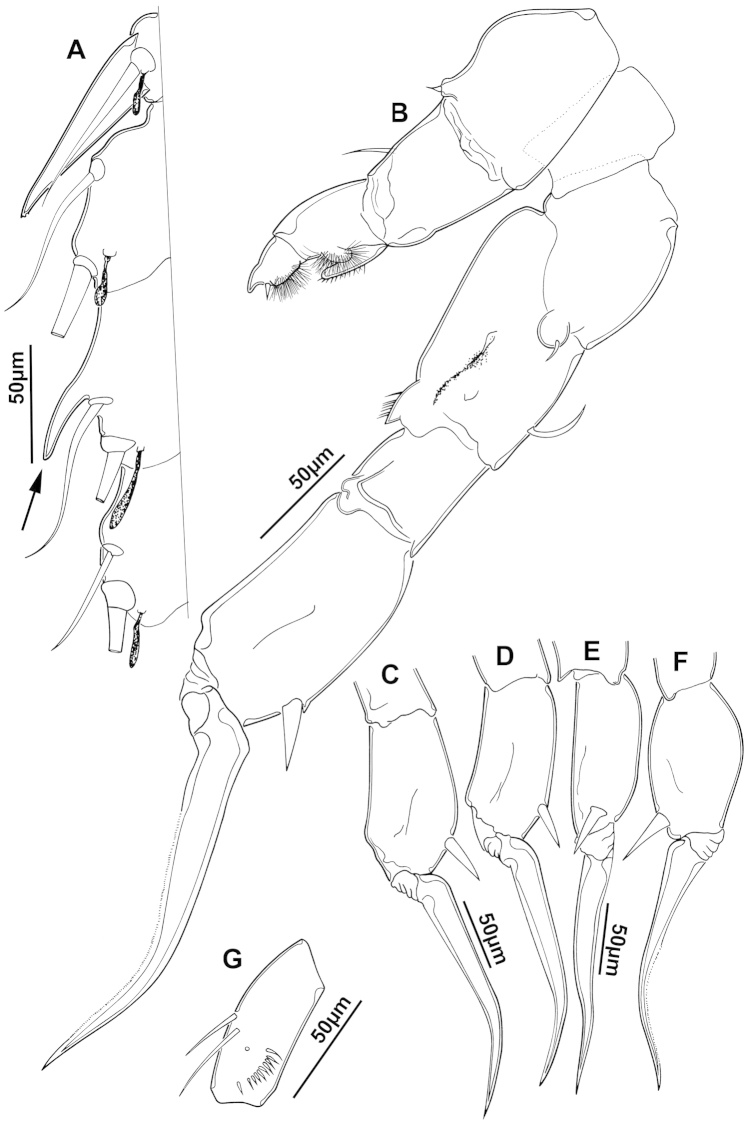
*Notodiaptomus conifer* male. **A** Segments 13–16 of A1R **B** P5; **C–F** Different views of ExpP5R **G** First segment of Enp A2.

**Figure 36.**
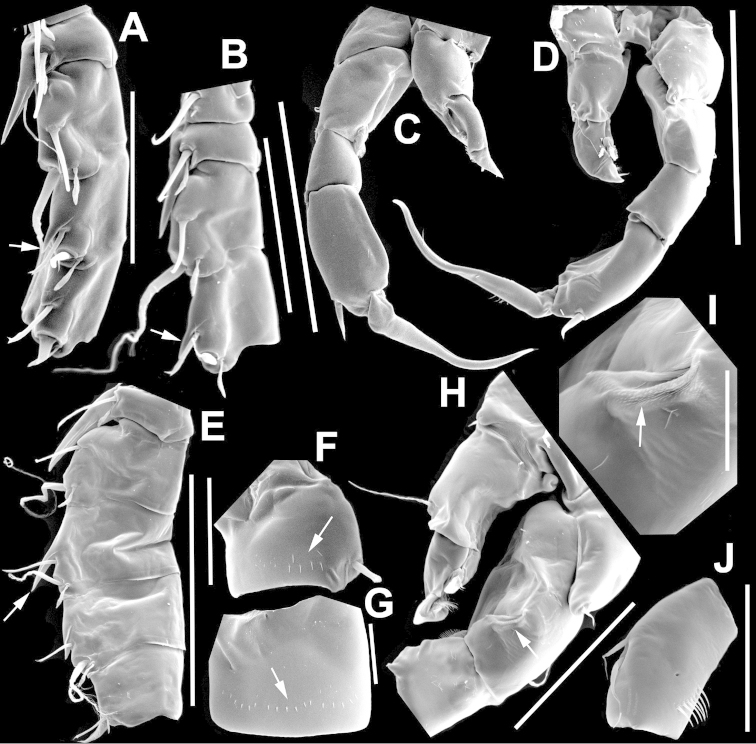
*Notodiaptomus conifer* male, SEM photographs. **A** Segments 13–16 of A1R (100 µm) **B** Segments 12–15 of A1R (100 µm) **C** Caudal view of P5 (100 µm) **D** Frontal view of P5 (100 µm) **E** Segments 13–17 of A1R (200 µm) **F, G** Segment 1 of A1R with spinule row arrowed (**F** = 50 µm; **G** = 20 µm) **H** P5L, and right Cx, Bsp and Exp1P5 (100 µm) **I** Detail of surface ornamentation of BspP5R (20 µm) **J** Segment 1 of Enp of A2 (50 µm).

**Figure 37.**
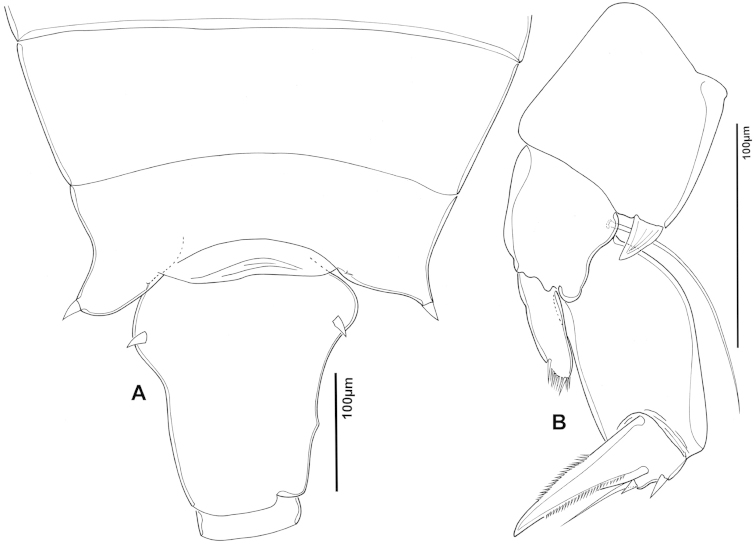
*Notodiaptomus conifer* female. **A** Dorsal view of posterior segments of prosome and GS **B** P5L.

**Figure 38.**
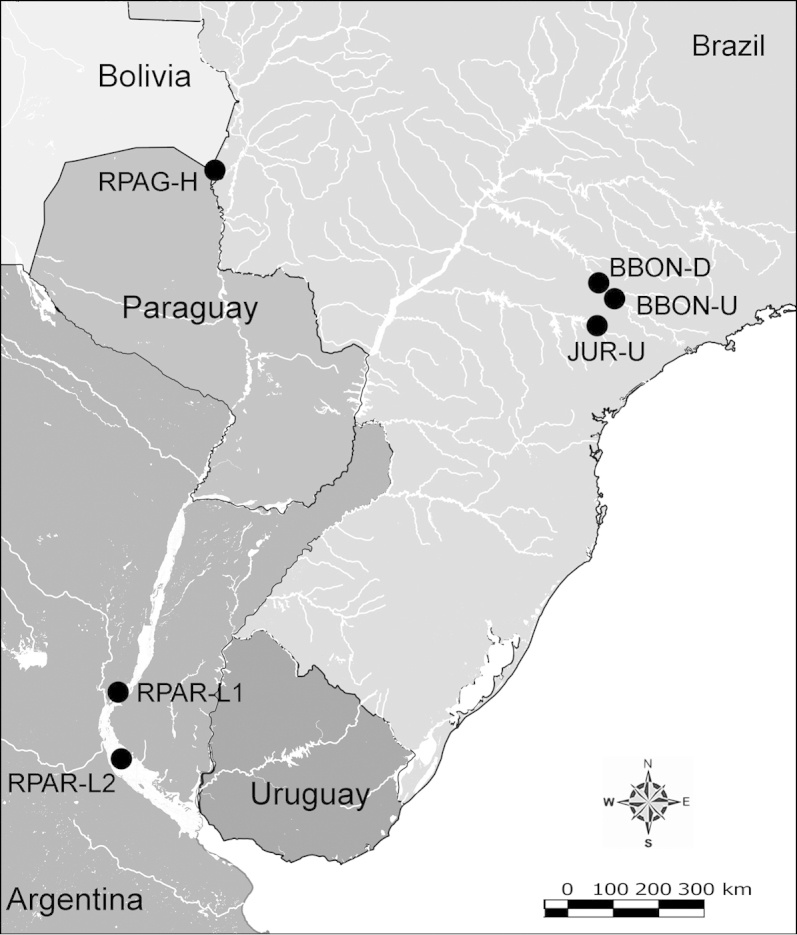
Geographical distribution of *Notodiaptomus conifer* in de la Plata river basin.

Figs 35 , 36 , 37 , 38

Diaptomus conifer Sars, 1901

###### Diagnosis.

**Adult male, body length 1548 µm.** Segment 1 of A1R with spinule row (Fig. 36F, G); segment 15 typically with well-developed spinous process reaching to end of segment (Figs 35A, 36A, B, E); process sometimes absent; segment 20 of A1R lacking distal projection. Enp1 of A2 ornamented with spinule row and single pore (Figs 35G, 36J). Right BspP5 with longitudinal fissure ornamented with surface granulations (Fig. 36H, I); Right Exp2P5 twice as long as wide (Figs 35B, 36C, D); lateral spine positioned close to outer distal angle of segment and directed posteriorly relative to longitudinal axis of body, length of lateral spine less than width of segment; terminal claw long and slightly curved (Fig. 35C–F), more slender than in *Notodiaptomus cearensis*.

**Adult female, body length 1734 µm.** Ped4 and Ped5 separated by complete suture; lateral wings slightly asymmetrical, each wing with sensilla at apex (Fig. 37A). GS asymmetrical, about 1.5 times longer than wide; slightly dilated anteriorly, with swelling on left side larger than on right, left swelling hemispherical with sensilla directed slightly posteriorly; right swelling more pronounced, sensilla located on dorsal surface, not on lateral margin; right lateral margin of GS with small projection about at 2/3 length and with small notch at posterior border (Fig. 37A). P5 symmetrical (Fig. 37B), with small expansion at outer distal corner of Cx, bearing large robust, triangular sensilla, approximately 1.5 times longer than wide. BspP5 with long outer seta, almost reaching distal margin of Exp1P5. EnpP5 with incomplete suture, similar in length to inner margin of Exp1P5. Exp 3-segmented; lateral spine of Exp2P5 similar in length to external margin of Exp3P5; external seta of Exp3P5 about 1/5 (20%) length of internal seta; internal seta attaining 3/4 length of terminal claw.

###### Remarks.

The illustrated specimens were collected from the upper Tiete River, at the Barra Bonita Reservoir. In addition to the well-developed spinous process on segment 15 of A1R of the male, *Notodiaptomus conifer* can be distinguished from other congeners like *Notodiaptomus iheringi* and *Notodiaptomus cearensis*, by its larger body size. These species also differ in their relative lengths of the lateral spines on the right Exp3P5 of the male.

Earlier studies (Nogueira 2001, Nogueira et al. 2008; Matsumura-Tundisi and Tundisi 2003), which sampled several rivers in São Paulo State (Brazil) between 1970 and 2005 noted changes in the abundance of *Notodiaptomus conifer*. Matsumura-Tundisi and Tundisi (2003) suggested that such changes might be in response to variations in the ionic concentrations resulting from decreasing water quality and increasing conductivity over the surveyed period. *Notodiaptomus conifer* was found in the current study (based on samples taken in 2010) in only two reservoirs (JUR and BBON) (Fig. 38), and in both of these Matsumura-Tundisi and Tundisi (2003) had indicated that the species had disappeared. Studies on long time series (e.g. Polli and Simona 1992) have demonstrated cycles of 25 years for some diaptomid species, over which they dramatically decrease in population density but subsequently recover to become dominant again. It is necessary to sample extensively before putative disappearances can be confirmed.

*Notodiaptomus conifer* has been reported from Argentina to the northeast of Brazil, thus suggesting a widespread but scattered occurrence across South America.

##### Notodiaptomus coniferoides

Taxon classificationAnimaliaCalanoidaDiaptomidae

(Wright, 1927)

**Figure 39.**
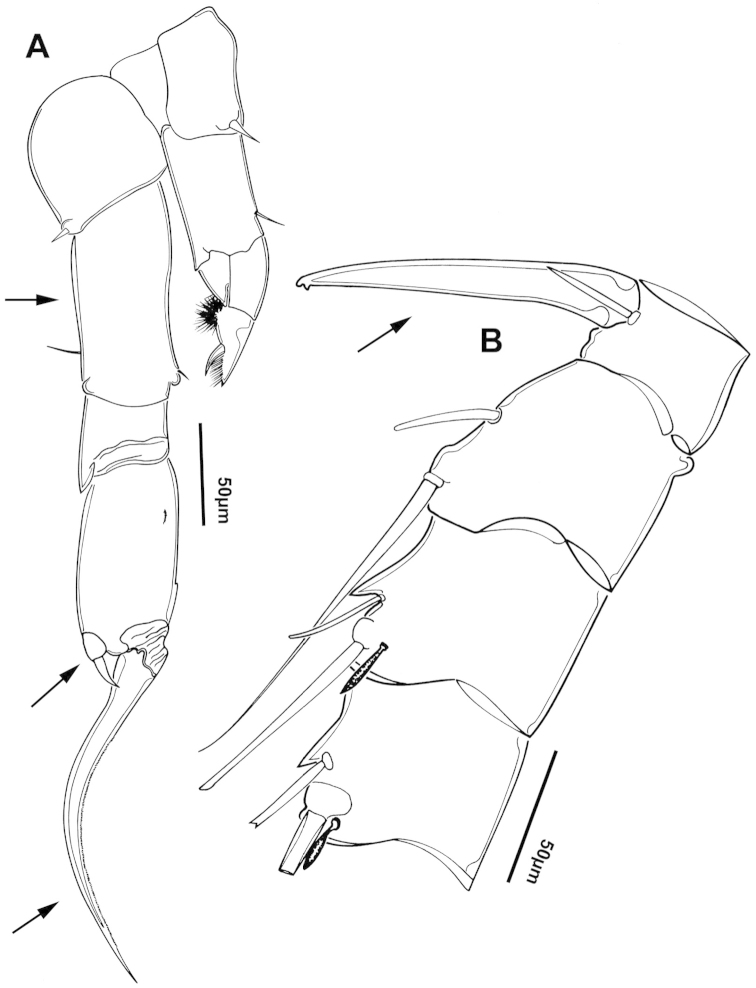
*Notodiaptomus coniferoides* male. **A** P5 **B** Segments 13–16 of A1R.

**Figure 40.**
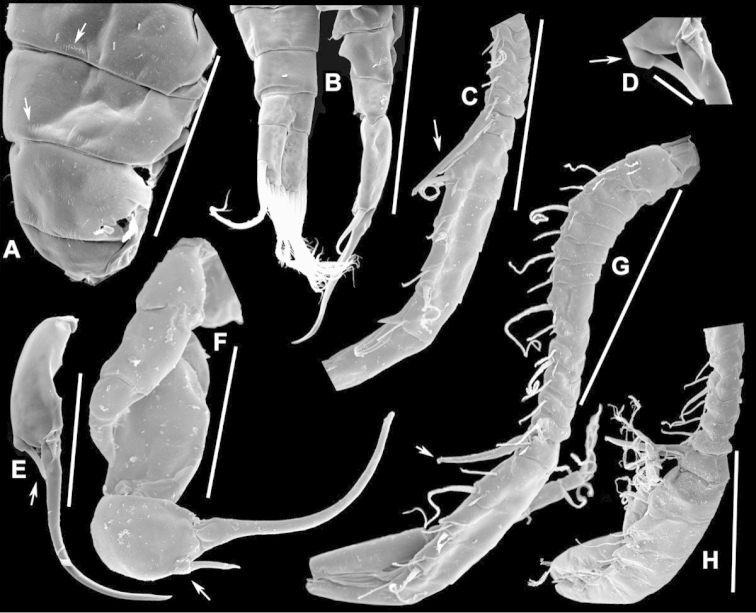
*Notodiaptomus coniferoides* male, SEM photographs. **A** Pedigers 2–5 (200 µm) **B** Last 3 urosome somites, CR, and P5R (300 µm) **C** Segments 9–18 of A1R (200 µm) **D** Detail showing insertion of lateral spine on Exp2P5R (20 µm) **E** Exp2P5R (100 µm) **F** P5 (100 µm) **G** A1R (300 µm) **H** A1R from segment 7 to tip (200 µm).

**Figure 41.**
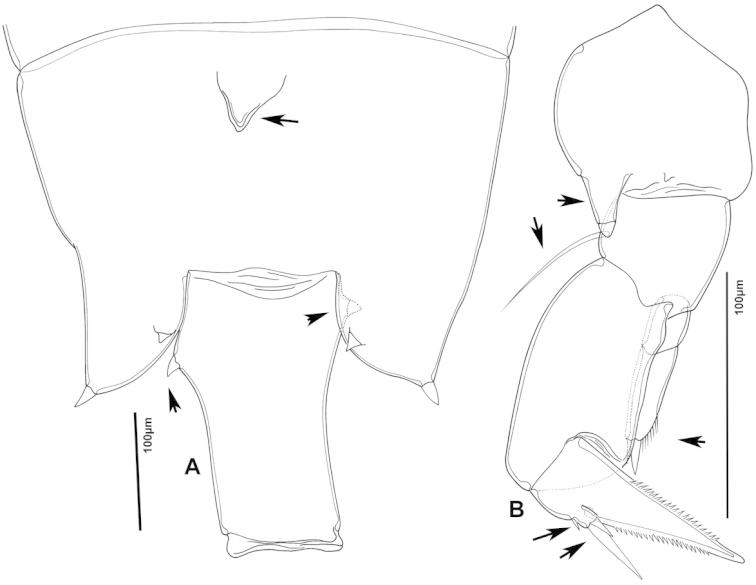
*Notodiaptomus coniferoides* female. **A** Posterior part of prosome and GS **B** P5.

**Figure 42.**
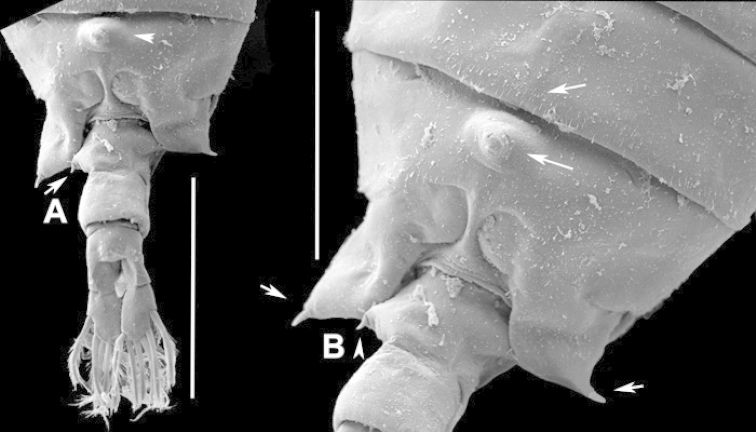
*Notodiaptomus coniferoides* female, SEM photographs. **A** Posterior part of prosome, and GS (300 µm) **B** Posterior view of prosome and GS, showing detail of dorsal process on Ped4 and dorsal rows of spinules (200 µm).

**Figure 43.**
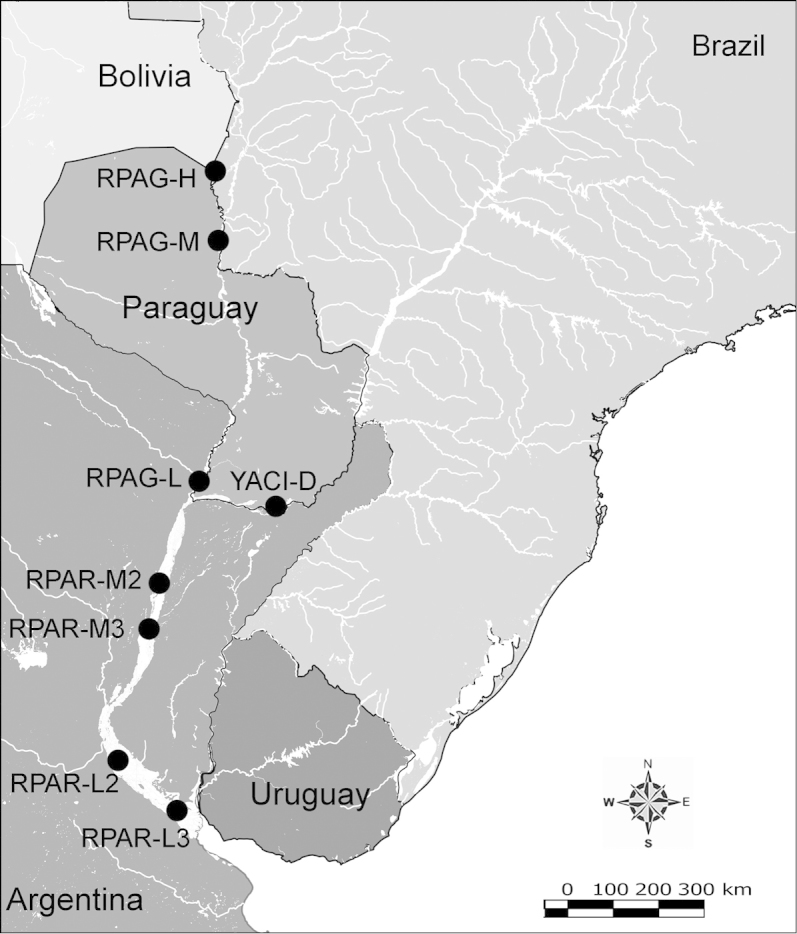
Geographical distribution of *Notodiaptomus coniferoides* in de la Plata river basin.

Figs 39 , 40 , 41 , 42 , 43

Diaptomus conferoides Wright, 1927
Diaptomus lobifer Pesta, 1927

###### Diagnosis.

**Adult male, body length 1051 µm.** Ped2, Ped3 and Ped4 ornamented with spinules near posterior margin (Fig. 40A). Ur3 to Ur5 lacking ventral spinulation (Fig. 40B). Segment 13 of A1R with strong spinous process (Fig. 39B) reaching distal margin of segment 14; segment 15 with short spinous process slightly larger than spinous process on segment 16 (Fig. 40C, G, H). Lateral spine of right Exp2P5 short, inserted on small lobe-like protuberance (Figs 39A, 40D, F); spine slightly curved towards terminal claw. Terminal claw smooth and relatively long (Fig. 40E) compared to other species, approximately twice length of internal margin of right Exp2P5.

**Adult female, body length 1411 µm.** Suture between Ped4 and Ped5 incomplete; conical dorsal process present on Ped4 (Figs 41A, 42A, B); lateral wings symmetrical, with two pairs of sensillae, larger pair on apex of each wing (Fig. 42B), each about twice as long as wide; inner pair of sensillae located dorsally near internal margin of each wing, each about as long as wide. GS asymmetrical, approximately 1.7/1.8 times longer than wide; anterior part slightly dilated, each anterior swelling with apical sensilla; left sensilla distinctly curved posteriorly, right sensilla aligned perpendicular to longitudinal body axis (Fig. 41A). P5 symmetrical with small conical process at outer distal corner of Cx bearing short, robust and triangular sensilla (Fig. 41B). BspP5 with external seta almost reaching middle of external margin of Exp1P5. EnpP5 2-segmented, almost reaching end of inner margin of Exp1P5; EnpP5 with 2 strong unequal apical spines. ExpP5 3-segmented; lateral spine of Exp2P5 almost as long as external margin of Exp3P5; internal seta on Exp3P5 about 3.5 times longer than external seta; internal seta reaching middle of terminal claw.

###### Remarks.

The specimens illustrated here were collected in the upper Paraguay River. This is the only calanoid species that was found throughout the Paraguay basin (Fig. 43). Frutos et al. (2006) previously reported this species from along the Paraguay River. We did not find it in the basin of the Paraná River or the Uruguay River but it was previously observed in the middle section of the Paraná River by Dussart and Frutos (1985). Santos-Silva (2008) mentioned a record from the Itaipu Reservoir in the upper Paraná River basin, and other reports indicate its presence in the lower Paraná River, near the delta (e.g. Ringuelet 1958). The type locality of this species is in the Amazon (Wright 1927).

This species has a wide distribution in rivers and associated systems like marginal ponds, but it is not typically recorded in reservoirs. This species is easily distinguishable by the position of the lateral spine on a lobe on the coxa of P5 and by the length of spinous processes on segments 13 and 15 of the male A1R. Some studies have reported this species under the name *Notodiaptomus coniferoide* (sic.) (see Matsumura-Tundisi 1986), but the name presented in the original description by Wright (1927) is *Notodiaptomus coniferoides*.

Cicchino et al. (2001) described a new species from the Amazon region, which they named *Notodiaptomus simmilimus*, and its etymology alluded to its close resemblance with *Notodiaptomus coniferoides*. Comparison of the specimens described here with the observations made by previous authors revealed some distinctive features. However, we consider that the specimens from Venezuela identified as *Notodiaptomus coniferoides* by Dussart (1984) are in fact *Notodiaptomus simmilimus*, based on the differences in the proportions of BspP5 and the size of the modified seta on segment 13 of A1R. Dussart (1984) and Cicchino et al. (2001) did not refer to differences in the size of the modified seta on this segment, which tends to be smaller in *Notodiaptomus simmilimus* than in *Notodiaptomus coniferoides*. In the Amazonian specimens of *Notodiaptomus coniferoides*, the modified seta reaches the end of segment 14, according to the original description (Wright 1927), whereas in *Notodiaptomus simmilimus* it only extends to the middle of this segment.

We recommend a thorough comparative analysis of specimens of *Notodiaptomus coniferoides* found in the south of Brazil and in the lower Parana River. Comparison of our *Notodiaptomus coniferoides* with Amazonian specimens indicates that the material described in the present study was relatively smaller and we consider it is necessary to confirm the identity and status of *Notodiaptomus coniferoides* in de la Plata River Basin.

##### Notodiaptomus dentatus

Taxon classificationAnimaliaCalanoidaDiaptomidae

Paggi, 2001

Figs 44 , 45 , 46

###### Diagnosis.

**Figure 44.**
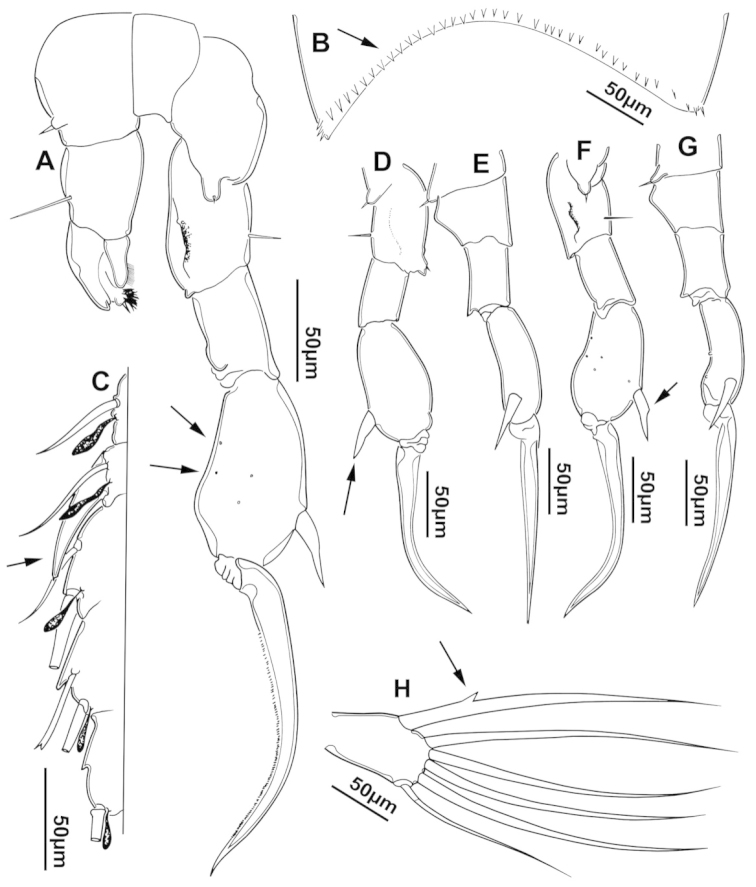
*Notodiaptomus dentatus* male. **A** P5 **B** Detail of spinular ornamentation on prosome 4 **C** Segments 12–16 of A1R **D–G** Different views of P5R **H** Caudal ramus showing tooth-like denticle on external seta.

**Adult male, body length 1046 µm.** Posterior margin of Ped4 ornamented with more or less regular row of spinules (Fig. 44B). Small denticle present on outer margin of external caudal seta, near base (Fig. 44H). Modified seta on segment 13 of A1R well developed with minutely bifid apex, reaching middle of segment 14 (Fig. 44C); segment 20 of A1R variable, with or without falciform process. Right BspP5 1.8 times longer than wide, with longitudinal fissure ornamented with surface granulations. Surface of right Exp3P5 with 3-6 sclerotized processes (Fig. 44A, F, G); lateral spine of Exp2P5R short (Fig. 44A, D, E).

**Figure 45.**
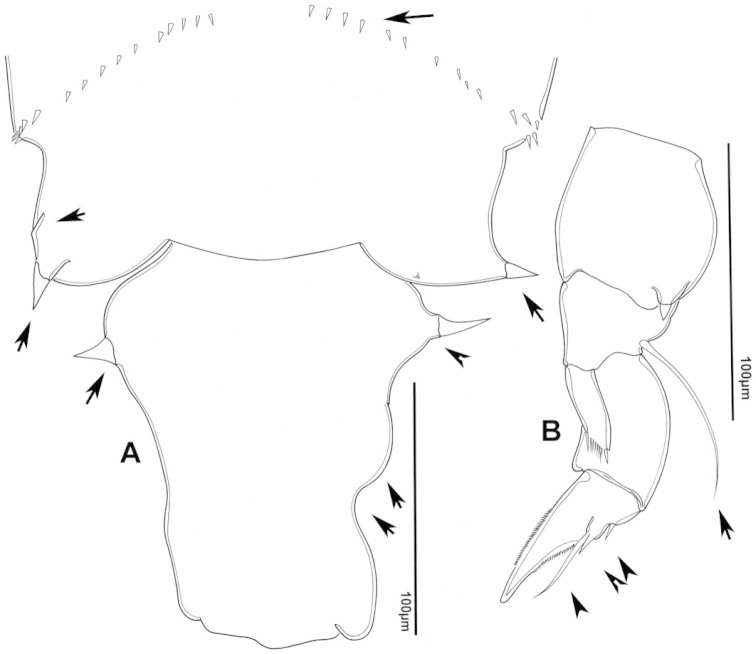
*Notodiaptomus dentatus* female. **A** Dorsal posterior part of prosome and GS **B** P5.

**Adult female, body length 1335 µm.** Incomplete suture present between Ped4 and Ped5, with spinule row marking plane of fusion (Fig. 45A); row of spinules present dorsally along posterior margin of Ped4 (Fig. 45A); lateral wings of similar size but slightly asymmetrical; two sensillae, one large and one small, present on each wing; apical sensilla on left side located on small projection and with apex directed posteriorly; right sensilla located at apex of projection directed perpendicular to body axis. GS asymmetrical (Fig. 45A), approximately 1.9 times longer than wide. Anterior part slightly dilated, left swelling larger than right one; left side hemispherical, with strong sensilla at apex of swelling, about 1.5 times longer than wide; right side ornamented with sensilla at apex of triangular projection, sensilla about twice as long as wide; posterior to sensillate swelling, external margin sinuous in dorsal view, ending in well-developed lobe at posterior end of right side. P5 symmetrical (Fig. 45B), with small conical process at outer distal corner of CxP5 bearing triangular sensilla, about 1.4 times longer than wide. BspP5 with long outer seta long, reaching almost to end of outer margin of Exp1P5. EnpP5 one-segmented, length exceeding middle of inner margin of Exp1P5. Exp 3-segmented; lateral spine of Exp2P5 just reaching end of external margin of Exp3P5; external seta of Exp3P5 about 1/4 (25%) length of internal seta; internal seta extending almost to tip of terminal claw.

###### Remarks.

**Figure 46.**
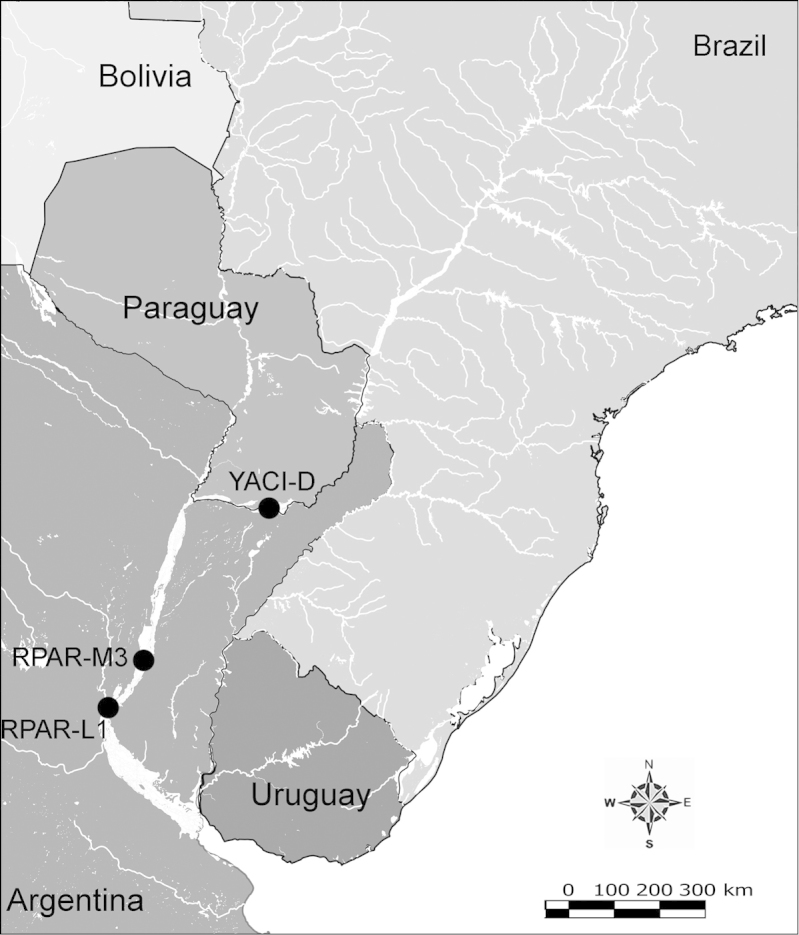
Geographical distribution of *Notodiaptomus dentatus* in de la Plata river basin.

The illustrated specimens were collected from the middle stretch of the Paraná River (Fig. 46, RPAR-M3). The body length of the sampled specimens is slightly less than that reported by Paggi (2001) but falls within the known range for this species. Paggi (2001) highlighted two important characteristics of this species: 1) the presence of sclerotized processes on the surface of the right Exp2P5 of the male, and 2) the presence of a small tooth-like denticle on the outer margin of external caudal seta, near its base. This tooth-like process provides the basis for the etymology of the species.

##### Notodiaptomus henseni

Taxon classificationAnimaliaCalanoidaDiaptomidae

(Dahl, 1894)

Figs 47 , 48 , 49 , 50 , 51 , 52 , 53 , 54

Diaptomus henseni Dahl, 1894
Notodiaptomus oliveirai Matsumura-Tundisi, Espindola, Tundisi, Souza-Soares & Degani, 2010 [new synonym]

###### Diagnosis.

**Figure 47.**
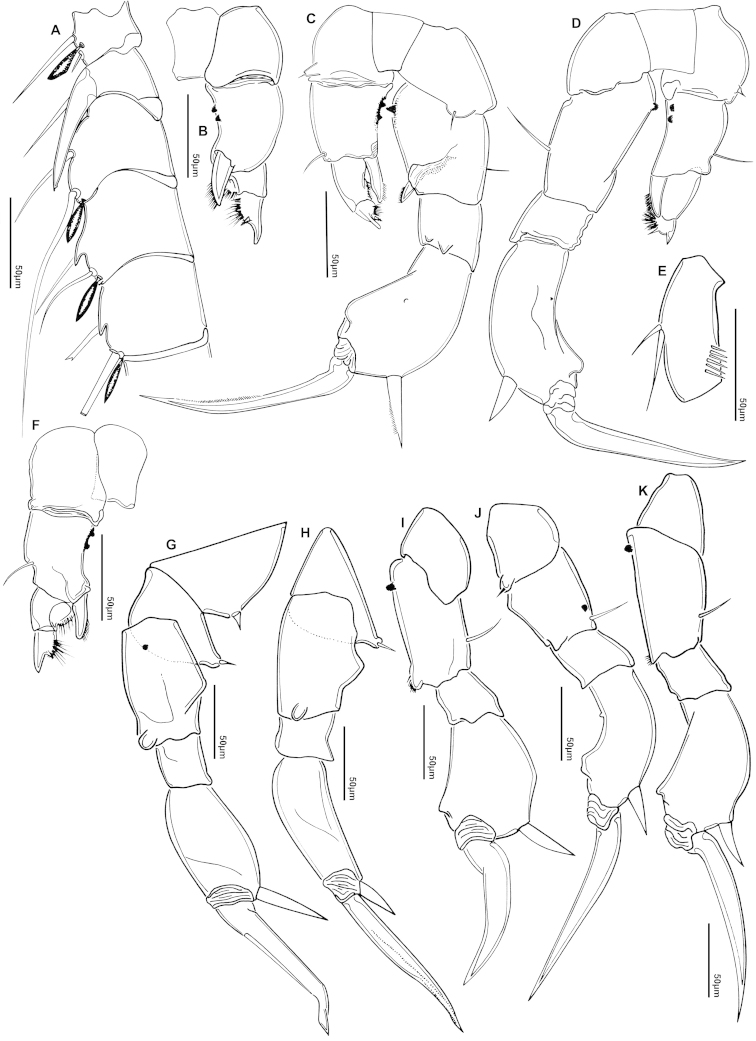
*Notodiaptomus henseni* male. **A** Segments 12–16 of A1R **B** Left P5 **C, D** Different views of P5 **E** Segment 1 of Enp of A2 **F** P5L **G–K** Different views of P5R.

**Figure 48.**
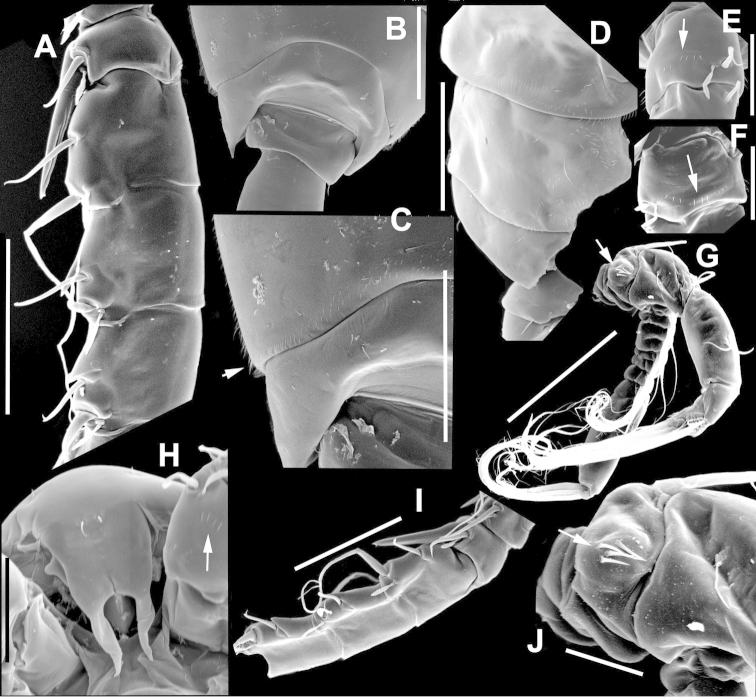
*Notodiaptomus henseni* male, SEM photographs. **A** Segments 13–16 of A1R (100 µm) **B** Ped4 and Ped5, and anterior part of urosome (100 µm) **C** Detail showing spinular ornamentation of Ped3 and Ped4 (100 µm) **D** Detail of spinules on Ped2-Ped4 (100 µm) **E, F** Segment 1 of A1R, showing spinular ornamentation (50 µm) **G** A2 (100 µm) **H** Rostrum and spinules on segment 1 of A1L (50 µm) **I** Segments 11–16 of A1R (100 µm) **J** Detail of Bsp of A2 (20 µm).

**Figure 49.**
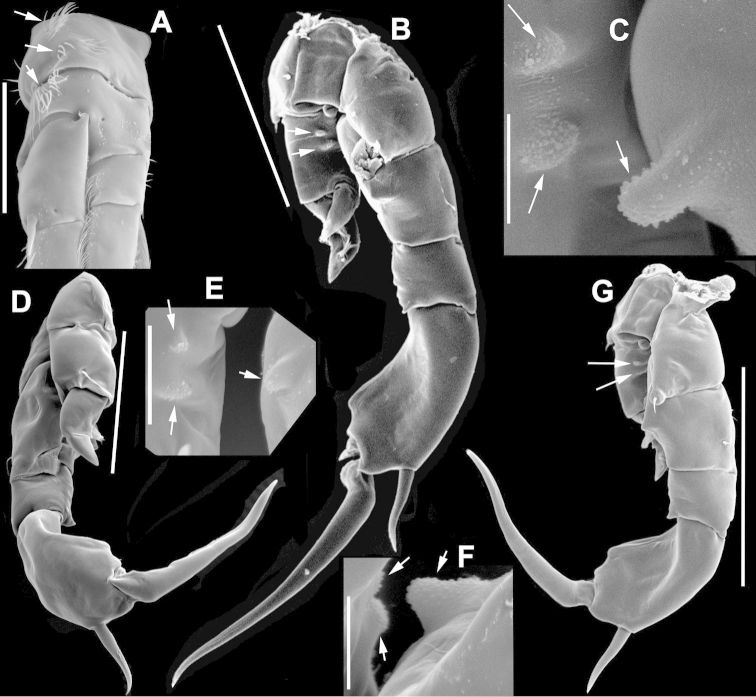
*Notodiaptomus henseni* male, SEM photographs. **A** P1, Details of spinular ornamentation on Cx and Bsp (50 µm) **B** Left and right P5 (150 µm) **C** Sclerotized processes on left and right BspP5 (10 µm) **D** P5 (100 µm) **E, F** Detail of sclerotized processes on left and right BspP5 (**E** = 20 µm; **F** = 10 µm) **G** P5 (200 µm).

**Adult male, body length 1123 µm.** Dorsal suture between Ped4 and Ped5 incomplete (Fig. 48B); surface of Ped3, Ped4, and Ped5 ornamented postero-dorsally and laterally with fine covering of slender spinules (Fig. 48C–D). Lateral wings small, slightly asymmetrical, directed posteriorly; left side larger than right; both lateral wings with short, posteriorly-directed sensilla at distal corner, left sensilla better developed than right (Fig. 48B). GS slightly asymmetrical. Rostrum asymmetrical (Fig. 48H). Segment 1 of A1R ornamented with spinule row (Fig. 48E, F); modified seta on segment 13 reaching to end of segment 14 (Figs 47A, 48A, I). Bsp of A2 ornamented with spinules (Fig. 48G, J); Enp1 of A2 with spinule row (Fig. 47E). Cx and Bsp of P1 ornamented with long setules laterally (Fig. 49A). Right CxP5 with conical process bearing sensilla at apex (Figs 47H, J, 49G). Inner margin of right BspP5 with sclerotized knob, covered with tiny granulations (Figs 47C, D, 49C, E, F); right BspP5 with oblique fissure ornamented with small surface granulations (Fig. 47C); external seta of right BspP5 inserted subdistally, short, typically shorter than outer margin of Exp1P5. Right Exp1P5 bearing triangular process distally; right Exp2P5 with internal margin concave proximally, lateral spine slightly curved (Figs 47K, 49D) and inserted sub-terminally on external margin, less than 1/3 length of terminal claw (Fig. 47G–K). CxP5L with small conical process at outer distal corner bearing short apical sensilla; BspP5L with short external seta inserted distally; internal margin of BspP5L slightly concave, bearing two small sclerotized knobs proximally (Figs 47B–D, 49B, G), each ornamented with tiny granulations (Fig. 49C, E, F). EndP5 one-segmented, with row of spinules on inner distal margin (Fig. 47B, F). Exp2P5L with digitiform and sclerotized terminal process, lacking ornamentation.

**Figure 50.**
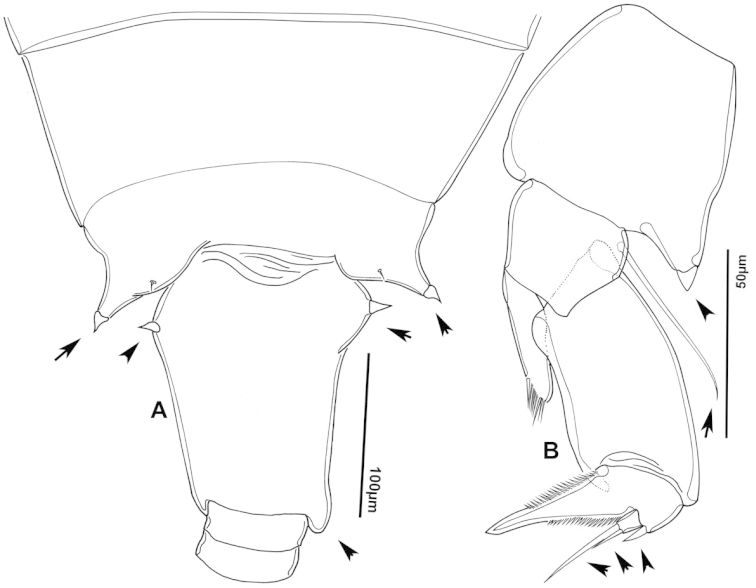
*Notodiaptomus henseni* female. **A** Posterior part of prosome, GS and urosome **B** P5.

**Figure 51.**
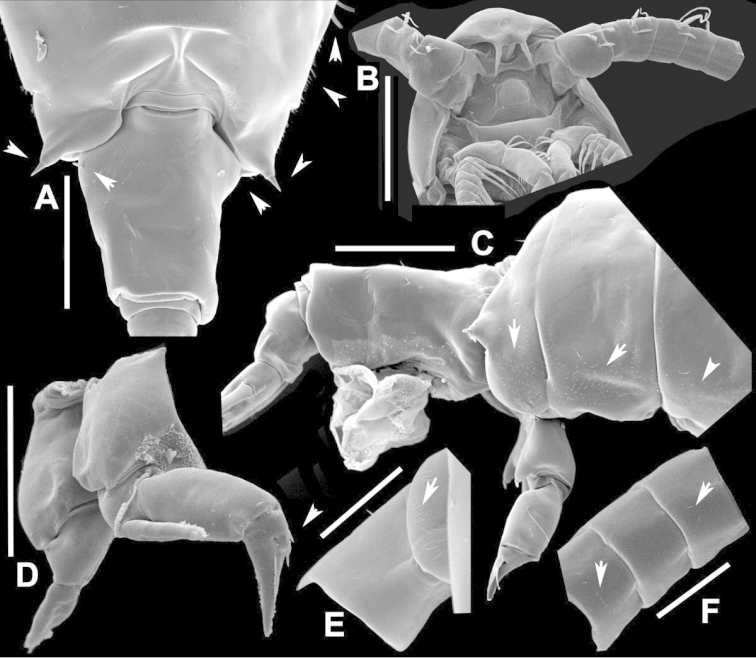
*Notodiaptomus henseni* female, SEM photographs. **A** Dorsal view of posterior part of prosome and GS (300 µm) **B** Ventral view of anterior end of cephalothorax, with rostrum and basal segments of antennule (150 µm) **C** Lateral view of posterior part of prosome, with arrows showing surface ornamentation of spinules, GS, urosome and CR (100 µm) **D** P5 (100 µm) **E** Dorsal view of segments 1 and 2 of left antennule, showing spinules (50 µm) **F** Dorsal view of segments 3–5 of left antennule, with some spinules (50 µm).

**Adult female, body length 1275 µm.** Complete suture present between Ped4 and Ped5 (Fig. 50A); lateral surfaces of Ped3, Ped4 and Ped5 ornamented with covering of fine spinules (Fig. 51C); lateral wings more or less symmetrical (Fig. 51A); both wings with two sensillae, larger sensilla located at apex of wing and about 1.5 times longer than wide, smaller sensilla located near posterior margin of wing (Fig. 50A). GS asymmetrical, about 1.9 to 2 times longer than wide; slightly dilated anteriorly, with swelling of similar size on each side; each swelling with sensilla at apex; sensilla on right side of GS located at apex of hemispherical swelling; small lobe located at posterior end of right margin of GS (Figs 50A, 51A). Rostrum symmetrical (Fig. 51B). Fine setules present on segments 1 and 3 to 5 of A1 (Fig. 51E, F). P5 symmetrical (Fig. 50B) with small conical process at outer distal angle of CxP5, with short and strong sensilla, barely longer than wide. BspP5 with long external seta, extending beyond middle of external margin of Exp1P5. EnpP5 extending to middle of internal margin of Exp1P5, with incomplete suture (Fig. 51D). Exp 3-segmented; external seta of Exp2P5 exceeding length of external margin of Exp3P5 and similar in length to external seta of Exp3P5; external seta of Exp3P5 about 40% length of internal seta. Internal seta about 2/3 length of terminal claw.

###### Remarks.

**Figure 52.**
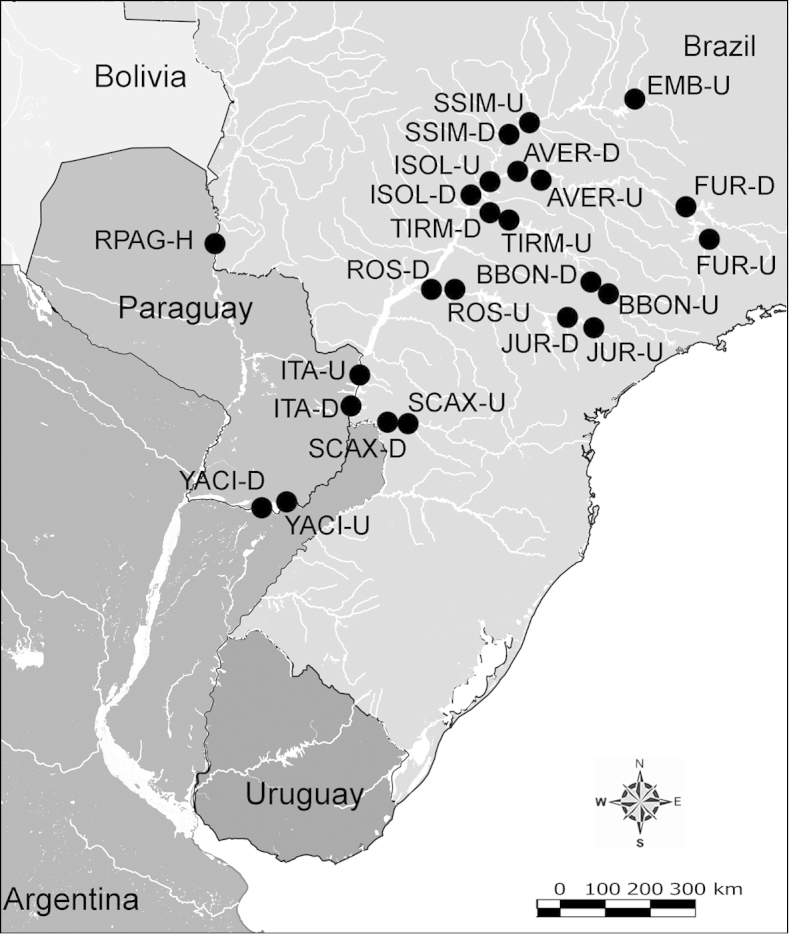
Geographical distribution of *Notodiaptomus henseni* in de la Plata river basin.

**Figure 53.**
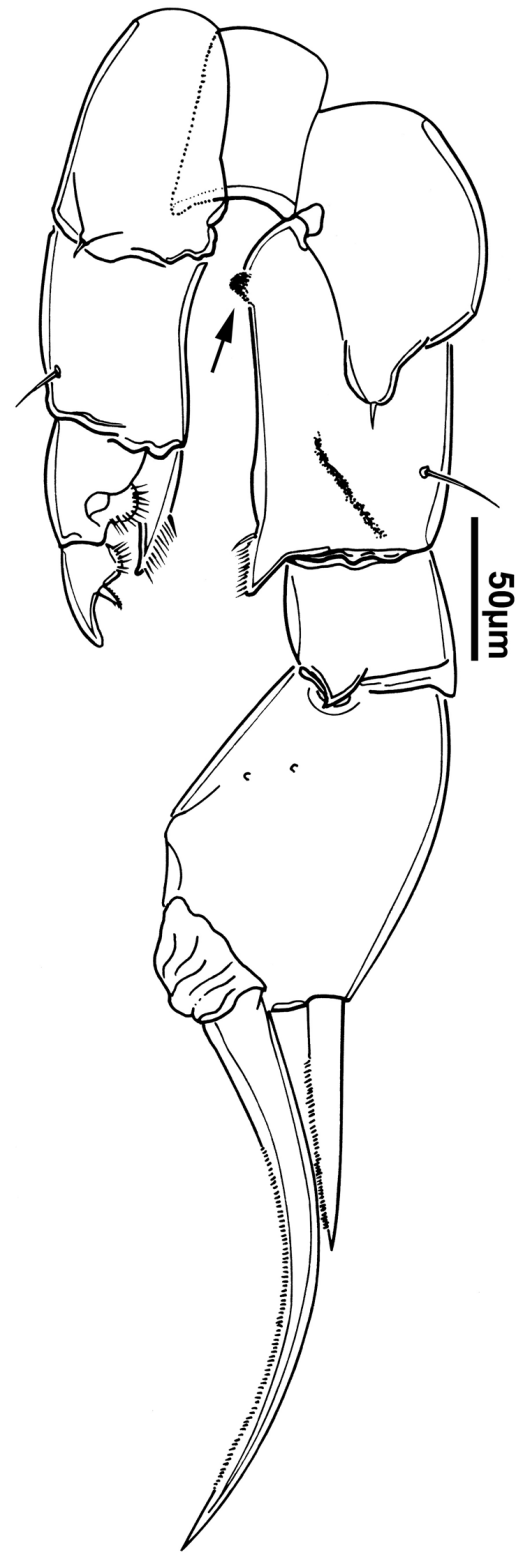
*Notodiaptomus amazonicus* male. P5. Specimen sampled in the Balbina Reservoir, Uatumã River (Central Amazonia), in the State of Amazonas, Brazil.

**Figure 54.**
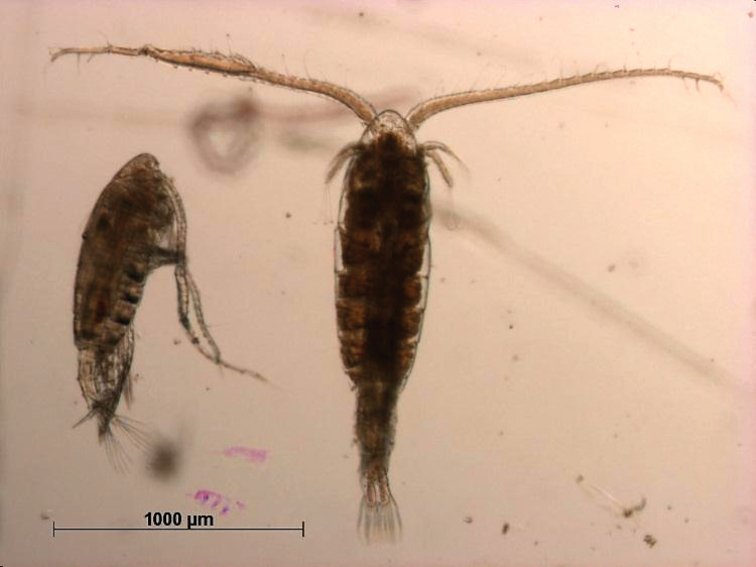
Light micrograph comparing males of *Notodiaptomus henseni* (on left) – specimen from the Grande River (FUR-D), and *Notodiaptomus amazonicus* (on right) – specimen from Uatumã River, State of Amazonas, Brazil.

The specimens depicted here were collected in Furnas Reservoir on the Grande River (Fig. 52, FUR-U). Wright (1936) commented on the possible confusion between *Notodiaptomus amazonicus* (Wright, 1935) and *Notodiaptomus henseni*. However, comparisons between the specimens of *Notodiaptomus henseni* found in this work and *Notodiaptomus amazonicus* collected in Balbina Reservoir (Uatumã River, Amazonas) showed clear differences in the structure of P5 (Fig. 53), the dorsal spinular ornamentation of the prosomal somites, and in body length. In the present study, specimens of *Notodiaptomus amazonicus* similar to those obtained in the Amazon region were not found, but *Notodiaptomus henseni* was found to be widely distributed in the Upper Paraná River. There are significant differences in size range between *Notodiaptomus henseni* and *Notodiaptomus amazonicus* (Fig. 54). *Notodiaptomus amazonicus* males had a body length of 1608 µm and a body width of 397 µm, both significantly larger than *Notodiaptomus henseni*, whose body length does not exceed 1300 µm. It is possible that some records of *Notodiaptomus amazonicus* from de la Plata basin are erroneous, reflecting the taxonomic confusion between this species and *Notodiaptomus henseni*. For example, Ringuelet and Martinez de Ferrato (1967) reported a body length of about 1270 µm for a diaptomid identified as *Notodiaptomus amazonicus* in Argentina. However, this length is much less than would be expected for this species and falls within the typical size range of *Notodiaptomus henseni*.

In addition, Ringuelet and Martinez de Ferrato (1967) mentioned that their specimens of *Notodiaptomus amazonicus* that carried a process on segment 20 of the male A1R were larger (about 1375 µm), a condition which is closer to the expected size of *Notodiaptomus amazonicus* from the Amazon region, although still relatively small. More comparisons are needed between specimens of *Notodiaptomus amazonicus* from the Amazon basin and those reported by other researchers from de la Plata River Basin.

Recently, Matsumura-Tundisi et al. (2010) described a new species, *Notodiaptomus oliveirai* Matsumura-Tundisi, Espindola, Tundisi, Souza-Soares & Degani, 2010, which shows a close similarity to *Notodiaptomus henseni*, and stated that many records of *Notodiaptomus henseni* from the state of São Paulo (Brazil) would represent records of *Notodiaptomus oliveirai*. However, their description is supported by illustrations lacking many important details (e.g. description of A1 formula, dorsal spinules) and the diagnostic difference between these two species as stated by these authors was based only on the curvature of the outer margin of the second segment of the exopod of right P5 (Exp2P5R). We consider this evidence insufficient to justify the establishment of a new species. The form of the P5 appears to vary according to the angle of observation (e.g. Fig. 47G to 47K), and this can be misleading. The degree of curvature alone does not provide adequate evidence upon which to base the establishment of a new species. The typical sclerotized processes on internal margins of left and right BspP5 of *Notodiaptomus henseni* are visible in the photograph of *Notodiaptomus oliveirai* included by Matsumura-Tundisi et al. (2010) and their presence provides further evidence pointing to the synonymy of this nominal species. We formally propose the recognition of *Notodiaptomus oliveirai* as a junior subjective synonym of *Notodiaptomus henseni*.

##### Notodiaptomus iheringi

Taxon classificationAnimaliaCalanoidaDiaptomidae

(Wright, 1935)

**Figure 55.**
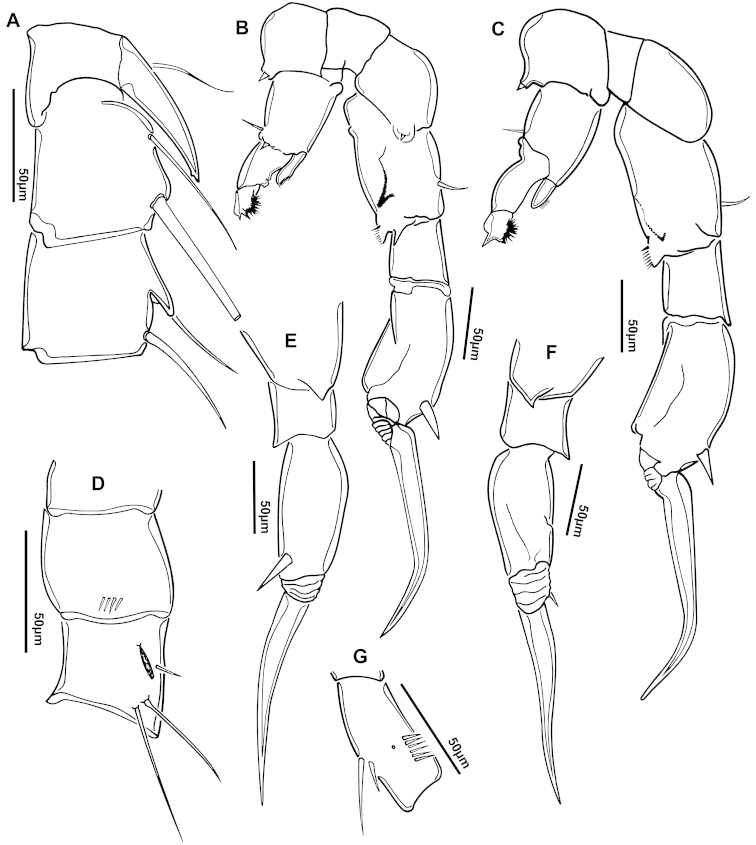
*Notodiaptomus iheringi* male. **A** Segments 13–15 of A1R **B, C** Different views of P5 **D** Segments 1 and 2 of A1R **E, F** Different views of terminal segments of ExpP5 **G** Segment 1 of Enp of A2.

**Figure 56.**
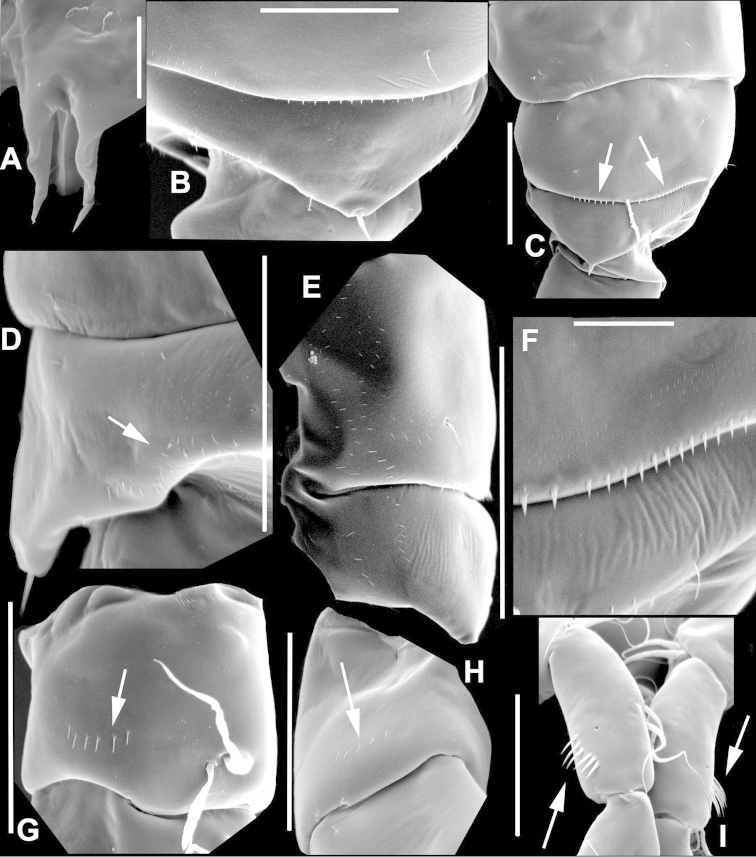
*Notodiaptomus iheringi* male, SEM photographs. **A** Rostrum (20 µm) **B** Ped4 and Ped5, lateral view (50 µm) **C** Ped3, Ped4, Ped5, lateral view (100 µm) **D** Detail of spinular ornamentation adjacent to sensilla on Ped5 (50 µm) **E** Ped4 and Ped5 (100 µm) **F** Ped3 and Ped4 (20 µm) **G, H** Segment 1 of A1R, showing row of spinules (50 µm) **I** Segment 1 of Enp of A2 (50 µm).

**Figure 57.**
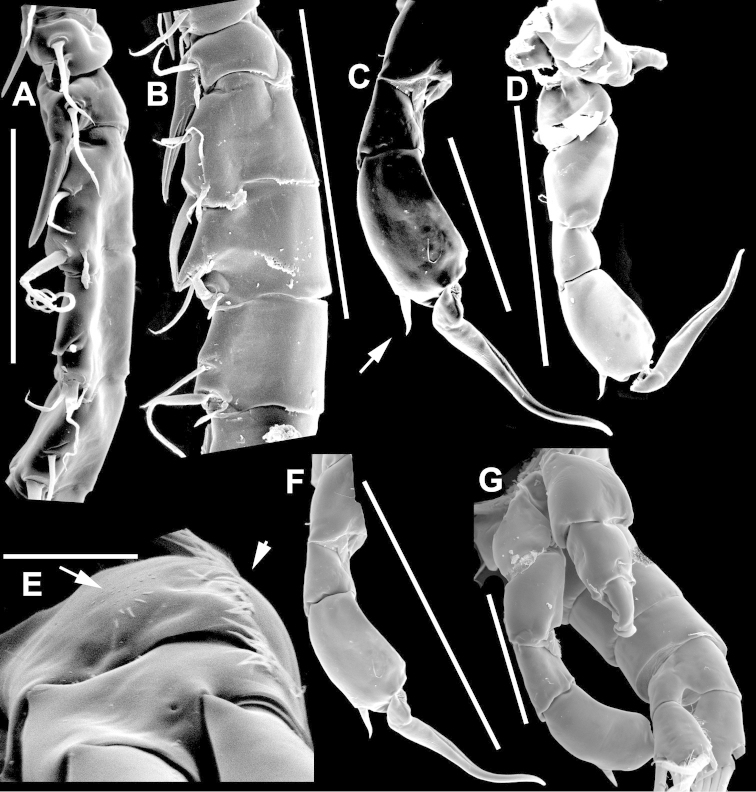
*Notodiaptomus iheringi* male, SEM photographs. **A** Segments 12–16 of A1R (100 µm) **B** Segments 13–16 of A1R **C, D** P5R (**C** = 100 µm; **D** = 200 µm) **E** Detail of spinules on Cx and BspP1 (20 µm) **F** P5R (200 µm) **G** P5 (100 µm).

**Figure 58.**
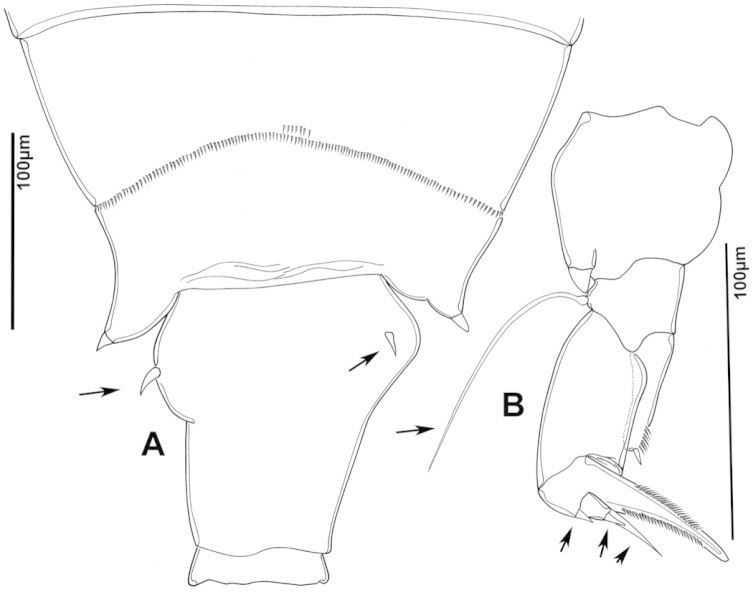
*Notodiaptomus iheringi* female. **A** Dorsal posterior part of prosome, GS and Ur2 **B** P5.

**Figure 59.**
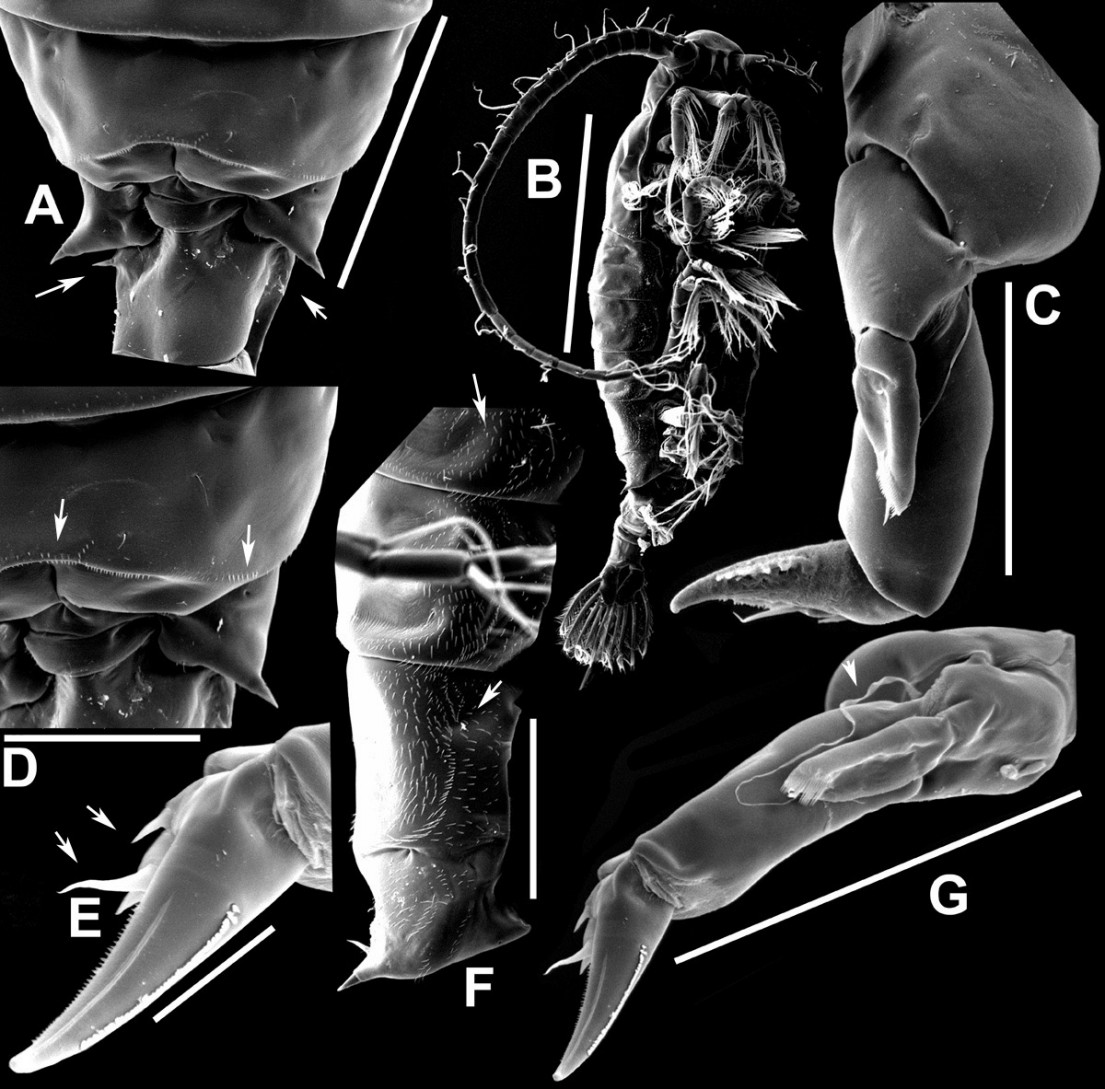
*Notodiaptomus iheringi* female, SEM photographs. **A** Dorsal posterior pedigers and GS (500 µm) **B** Male, ventral view (500 µm) **C** P5L (100 µm) **D** Posterior pedigers (100 µm) **E** Exp3P5 and terminal claw (20 µm) **F** Ventral view of posterior part of prosome (100 µm) **G** P5L (100 µm).

**Figure 60.**
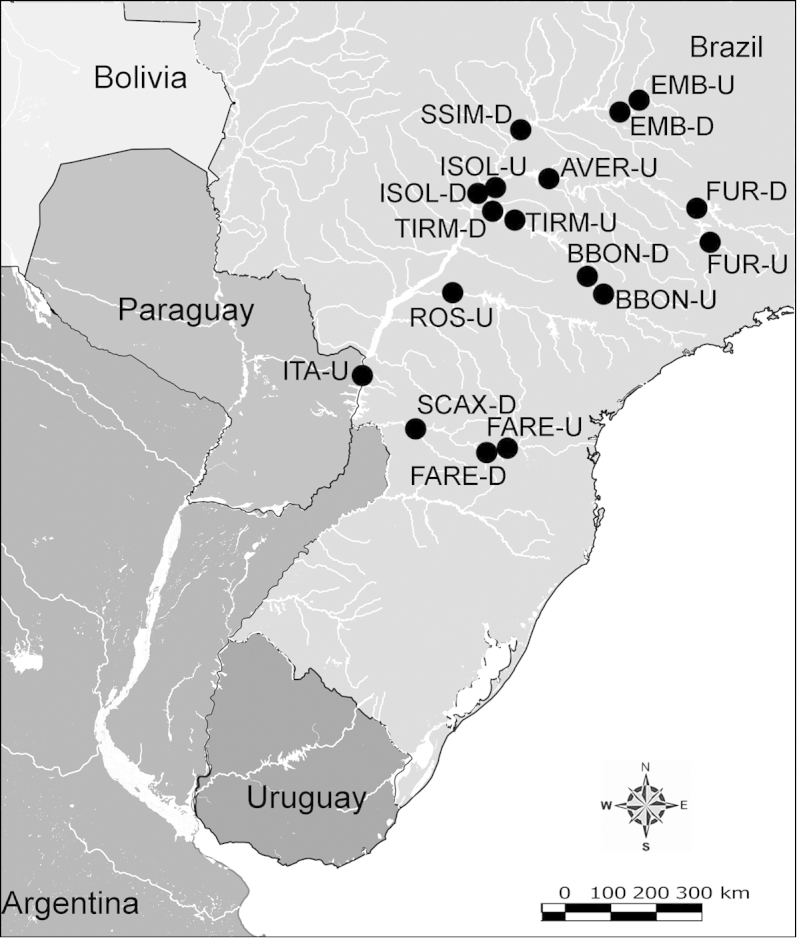
Geographical distribution of *Notodiaptomus iheringi* in de la Plata river basin.

Figs 55 , 56 , 57 , 58 , 59 , 60

Diaptomus iheringi Wright, 1935

###### Diagnosis.

**Adult male, body length 922 µm.** Posterior margin of Ped3 ornamented with rows of spinules; Ped4 and Ped5 ornamented with spinule rows along posterior margins and on lateral surfaces (Fig. 56B–F). Rostral filaments asymmetrical (Fig. 56A). Single row of spinules present on first segment of A1R (Figs 55D, 56G, H); modified seta on segment 13 of A1R strong, with minutely bifid apex, reaching beyond level of insertion of proximal seta on segment 14 (Fig. 55A); spinous process of segment 15 longer than spinous process of segment 16) (Fig. 57A, B). Enp1 of A2 ornamented with pore and spinule row (Figs 55G, 56I). Cx of P1 with setules on outer surface (Fig. 57E). Right and left internal margins of P5 without sclerotized processes (Figs 55B, C, 57G). Right BspP5 with irregular oblique fissure on middle of surface; distal part of fissure ornamented with small surface granulations; external seta inserted distally on lateral margin. Lateral spine on right Exp2P5 inserted in distal third of external margin, length about 1/6 (16%) of length of terminal claw (Figs 55E, F, 57C, D, F, G).

**Adult female, body length 1093 µm.** Incomplete suture present between Ped4 and Ped5 with plane of fusion marked by transverse row of strong spinules, with double row in middle section of dorsal surface (Figs 58A, 59D); lateral surfaces of posterior prosomal somites ornamented with spinules (Fig. 59F). Lateral wings bearing two unequal sensillae; large sensilla located at apex about 1.5 times longer than wide. GS asymmetrical, about 1.3 to 1.4 times longer than wide; dilated anteriorly, swellings of similar size; left swelling hemispherical; both swellings bearing sensilla approximately 2.5 times longer than wide, right sensilla inserted on dorso-lateral surface and not on apex of swelling (Figs 58A, 59A). P5 symmetrical (Fig. 58B) with small conical process at outer distal corner of Cx bearing short, robust triangular sensilla, about 1.1 times longer than wide. BspP5 with long external seta, extending beyond distal end of external margin of Exp1P5 (Fig. 58B). EnpP5 one-segmented (Fig. 59C, G), about 3/4 length of internal margin of Exp1P5. Exp 3-segmented; lateral spine of Exp2P5 not reaching end of external margin of Exp3P5; external seta of Exp3P5 approximately 3.5 times shorter than internal seta; internal seta reaching just beyond middle of terminal claw (Fig. 59D).

###### Remarks.

Our specimens were taken in the Grande River at Furnas Reservoir. In the present study this species was found in southeastern and southern Brazil and in the upper part of the Paraná River basin, with its southerly distribution boundary represented by the Iguaçu River (Fig. 60). Other studies (Santos-Silva 2008) indicate a widespread distribution in Brazil, and the type locality is in Paraiba State in northeastern Brazil, but it also occurs in some parts of northern Argentina. This species can be confused with its congeners *Notodiaptomus cearensis* and *Notodiaptomus isabelae* (Wright, 1936), but details of the male A1R and P5 are useful to distinguish *Notodiaptomus iheringi* from these other two species. This species also resembles *Notodiaptomus conifer* in possessing a small lateral spine on the P5, but *Notodiaptomus conifer* has a well-developed spinous process on segment 15 of A1R (Fig. 35A), which is much larger than that of *Notodiaptomus iheringi* (Fig. 55A). In the present study this species occurred in a variety of habitats with different trophic states, from oligotrophic, such as the Emborcação and Furnas reservoirs, to the eutrophic Barra Bonita and Foz do Areia reservoirs. Its presence or absence, therefore, is not indicative of trophic status and this species is not suitable for biomonitoring purposes.

##### Notodiaptomus incompositus

Taxon classificationAnimaliaCalanoidaDiaptomidae

(Brian, 1926)

**Figure 61.**
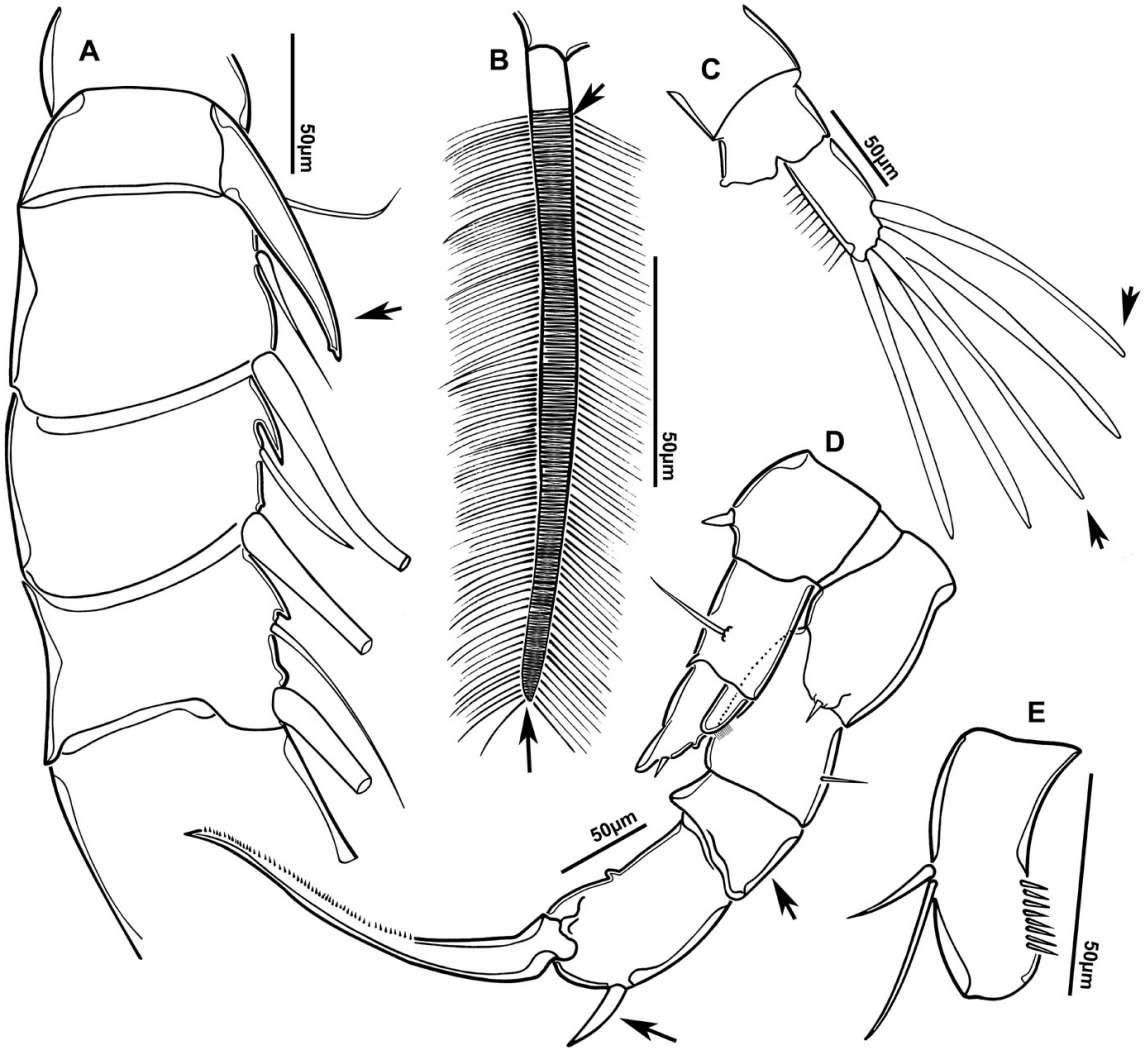
*Notodiaptomus incompositus* male. **A** Segments 13–16 of A1R **B** Caudal seta showing reticulate form **C** Dorsal view of CR **D** P5 **E** First segment of Enp of antenna.

**Figure 62.**
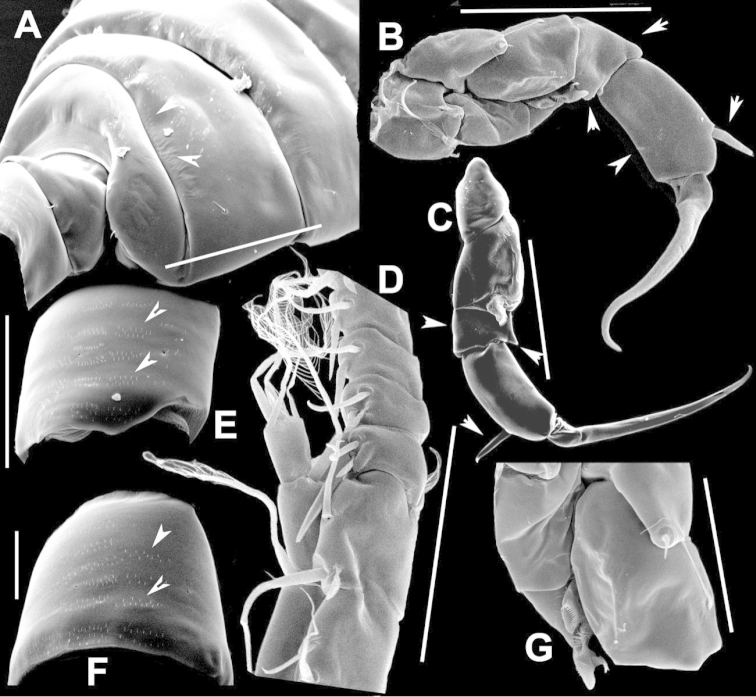
*Notodiaptomus incompositus* male, SEM photographs. **A** Dorsal view of Ped3, Ped4, Ped5 and GS (100 µm), showing detail of dorsal rows of spinules **B, C** P5 (100 µm) **D** Segments 10–15 of A1R (100 µm) **E** Dorsal view of Ur4 (50 µm) **F** Dorsal view of Ur3 (20 µm) **G** P5L, and Cx and Bsp of P5R (100 µm).

**Figure 63.**
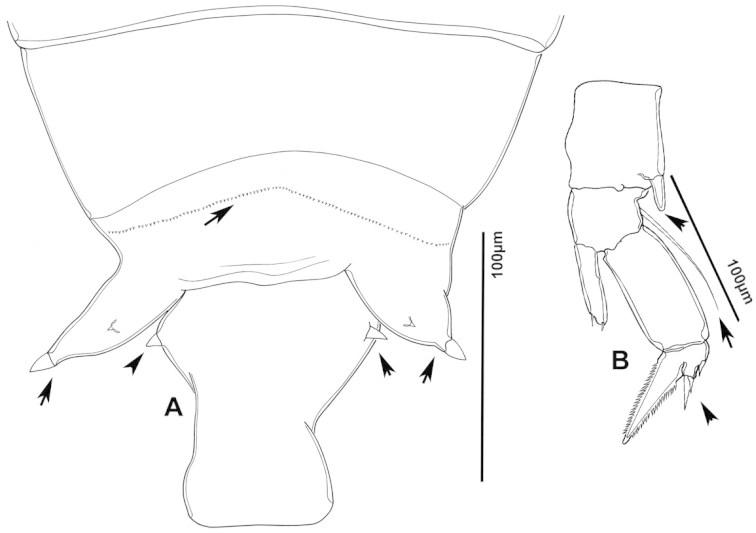
*Notodiaptomus incompositus* female. **A** Posterior part of prosome and GS, showing details of dorsal spinule row **B** P5.

**Figure 64.**
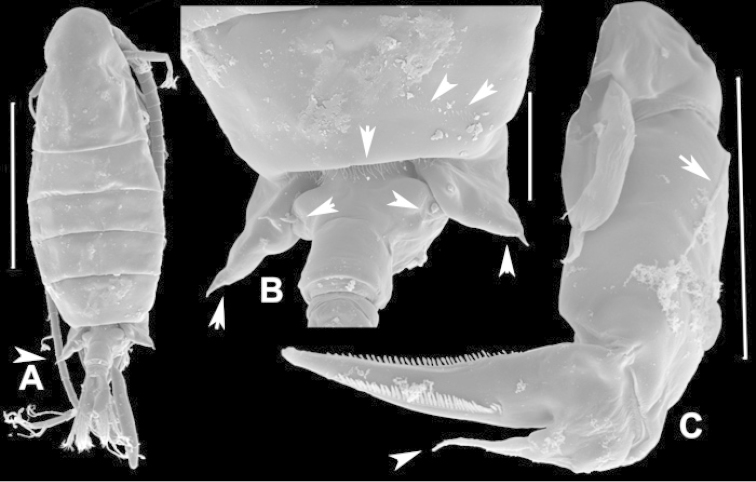
*Notodiaptomus incompositus* female, SEM photographs. **A** Dorsal view (100 µm) **B** Posterior pedigers and GS (50 µm) **C** P5 (100 µm).

**Figure 65.**
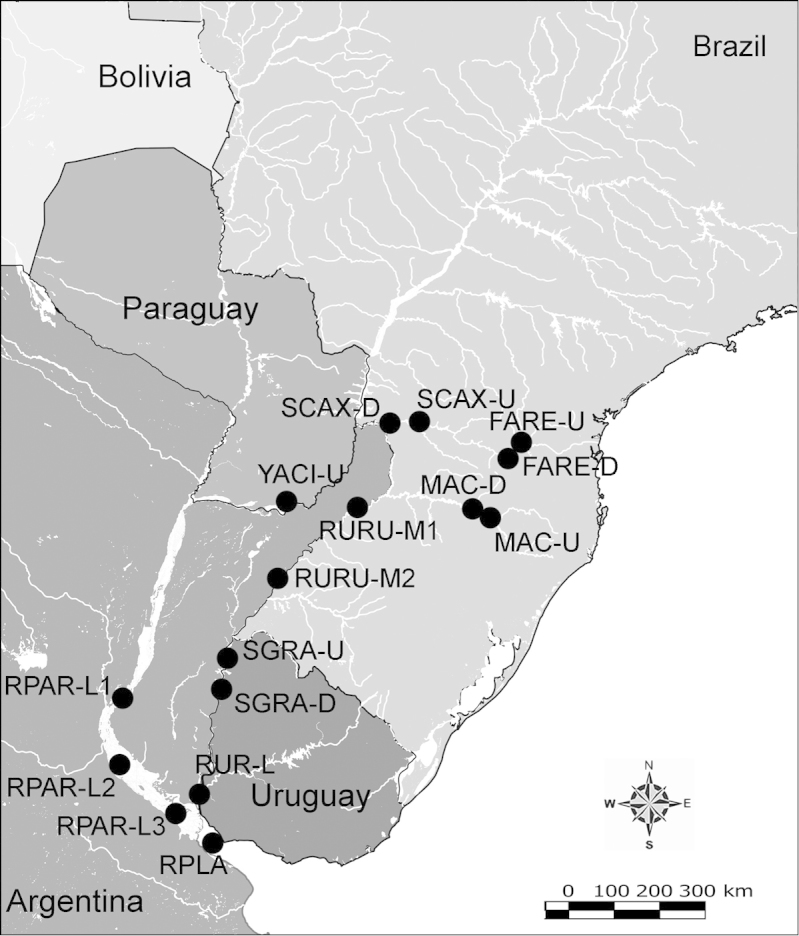
Geographical distribution of *Notodiaptomus incompositus* in de la Plata river basin.

Figs 61 , 62 , 63 , 64 , 65

Diaptomus incompositus Brian, 1926
Diaptomus paranaensis Pesta, 1927

###### Diagnosis.

**Adult male, body length 1029 µm.** Rows of spinules present dorsally along posterior margins of Ped3 and Ped4 (Fig. 62A), and dorsal surfaces of Ur3 and Ur4 extensively ornamented with patches of small spinules (Fig. 62E, F). Caudal setae modified; each seta with strong plumose setules bilaterally and rounded apex (Fig. 61B, C). Modified seta of segment 13 of A1R strong, with bifid apex and not extending beyond distal margin of segment 14; spiniform process on segment 15 larger than on segment 16 (Figs 61A, 62D). Enp1 of A2 ornamented with pore and spinule row (Fig. 61E). Right BspP5 with internal and outer margins smooth (Fig. 62G). Right Exp1P5 longer than wide; right Exp2P5 cylindrical, internal margin with sclerotized process near middle (Figs 61D, 62B); lateral spine of Exp2P5 inserted subterminally, slightly curved, short, less than 1/3 length of terminal claw (Fig. 62C).

**Adult female, body length 1310 µm.** Complete suture present between Ped4 and Ped5; transverse row of short spinules present along posterior margin of Ped4 (Fig. 63A); row of long setules, present dorsally on Ped5 (Fig. 64B); lateral wings asymmetrical (Fig. 64A, B), left wing narrower than right, each with two unequal sensillae, large sensilla at apex of wing. Sensilla about 1.5 times longer than wide. GS asymmetrical, approximately 1.5 times longer than wide: slightly dilated anteriorly, with swelling on right side larger than left; sensilla present at apex of each swelling, slightly on dorso-lateral surface on right side (Fig. 63A). P5 symmetrical (Figs 63B, 64C) with small conical process at outer distal corner of CxP5 bearing long blunt sensilla, approximately 2.6 times longer than wide. BspP5 with long external seta, equal in length to external margin of Exp1P5. EnpP5 approximately 3/4 of length of internal margin of Exp1P5. Exp 3-segmented; lateral spine of Exp2P5 as long as external margin of Exp3P5; internal seta of Exp3 about 2.3 times longer than external seta; internal seta reaching almost to middle of terminal claw (Fig. 64C).

###### Remarks.

This species was collected in the Machadinho Reservoir (MAC-U) on the Uruguay River and it can be easily identified by the unusual form of the caudal setae. It is distributed across the southern part of de la Plata river basin, including southern Brazil, south of the Iguaçu River (Fig. 65), and it also occurs widely in Argentina and Uruguay. In the present study, *Notodiaptomus incompositus* was among the dominant species and it tended to occur abundantly in reservoirs and river stretches irrespective of their trophic status (eutrophic, mesotrophic, and oligotrophic), as observed for *Notodiaptomus henseni*. In general, *Notodiaptomus henseni* was the dominant species in the northern part of de la Plata river basin, while *Notodiaptomus incompositus* was dominant in the southern sector.

##### Notodiaptomus isabelae

Taxon classificationAnimaliaCalanoidaDiaptomidae

(Wright, 1936)

Figs 66 , 67 , 68

Diaptomus isabelae Wright, 1936

###### Diagnosis.

**Figure 66.**
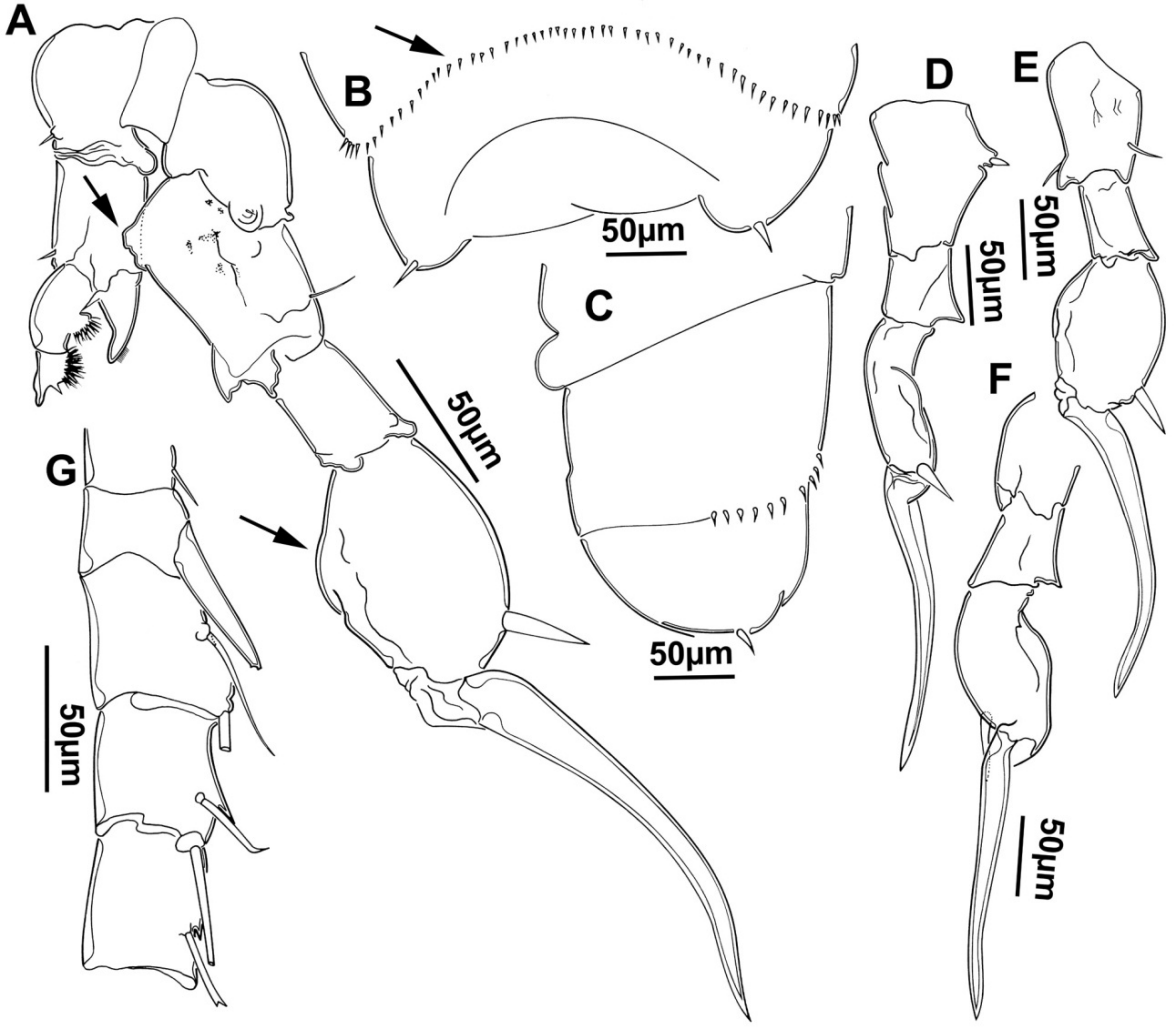
*Notodiaptomus isabelae* male. **A** P5 **B** Ornamentation of spinules on Ped4 **C** Lateral view of Ped3, Ped4 and Ped5, showing details of the spinule rows **D–F** Different views of P5R **G** Segments 12–16 of A1R.

**Adult male, body length 919 µm.** Transverse row of strong spinules present along posterior margin of Ped4 (Fig. 66B, C). Modified seta on segment 13 of A1R extending beyond middle of segment 14, but not reaching end of segment; spinous process present on segments 15 and 16 of A1R (Fig. 66G). Two small expansions present proximally on internal margin of right BspP5 (arrowed in Fig. 66A); right Exp1P5 with wide distal expansion on margin (Fig. 66D, F); right Exp2P5 broad, with width about ¾ length (Fig. 66A, E); lateral spine short, inserted distally, close to insertion of terminal claw.

**Figure 67.**
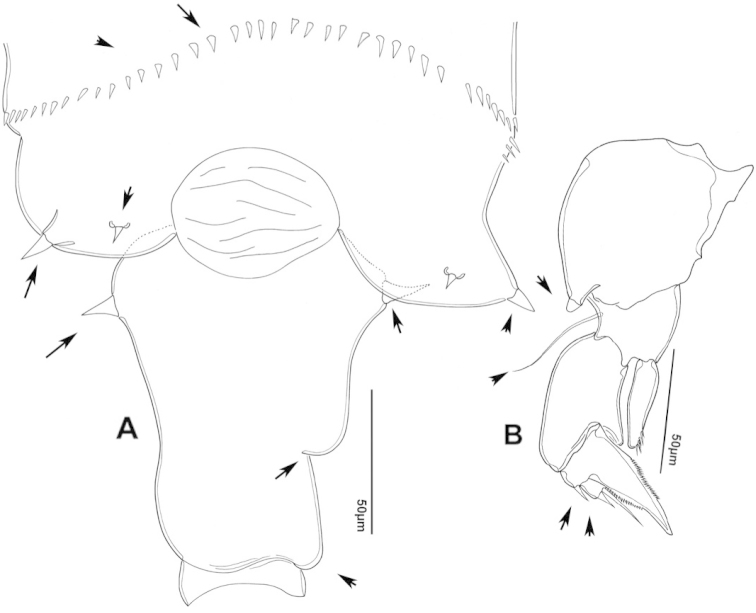
*Notodiaptomus isabelae* female. **A** Dorsal posterior pedigers and GS **B** P5.

**Adult female, body length 1056 µm.** Complete suture present between Ped4 and Ped5, ornamented with row of strong spinules dorsally (Fig. 67A); lateral wings asymmetrical, left wing smaller and located anterior to right wing; two pairs of sensillae present on each wing, large sensilla on left positioned on small dorsal projection; large sensilla on right positioned at apex of wing. GS asymmetrical, approximately 2.5 times longer than wide; anterior part slightly dilated, with swelling on right side larger than that on left; left swelling rounded with strong sensilla at apex, approximately 1.5 times long than wide; anterior swelling on right side with strong sensilla at apex, approximately twice as long as wide; anterior swelling extending back along two thirds of lateral margin to acute indentation; margin straight posterior to indentation (Fig. 67A). P5 symmetrical (Fig. 67B) with small conical process at outer distal corner of CxP5, bearing sensilla with rounded apex, barely longer than wide. BspP5 with external seta reaching middle of external margin of Exp1P5. EnpP5 one-segmented, about as long as inner margin of Exp1P5. ExpP5 three-segmented; lateral spine of Exp2P5 almost reaching distal tip of external margin of Exp3P5; external seta of Exp3P5 about one third length of internal seta; internal seta about two thirds length of terminal claw.

###### Remarks.

**Figure 68.**
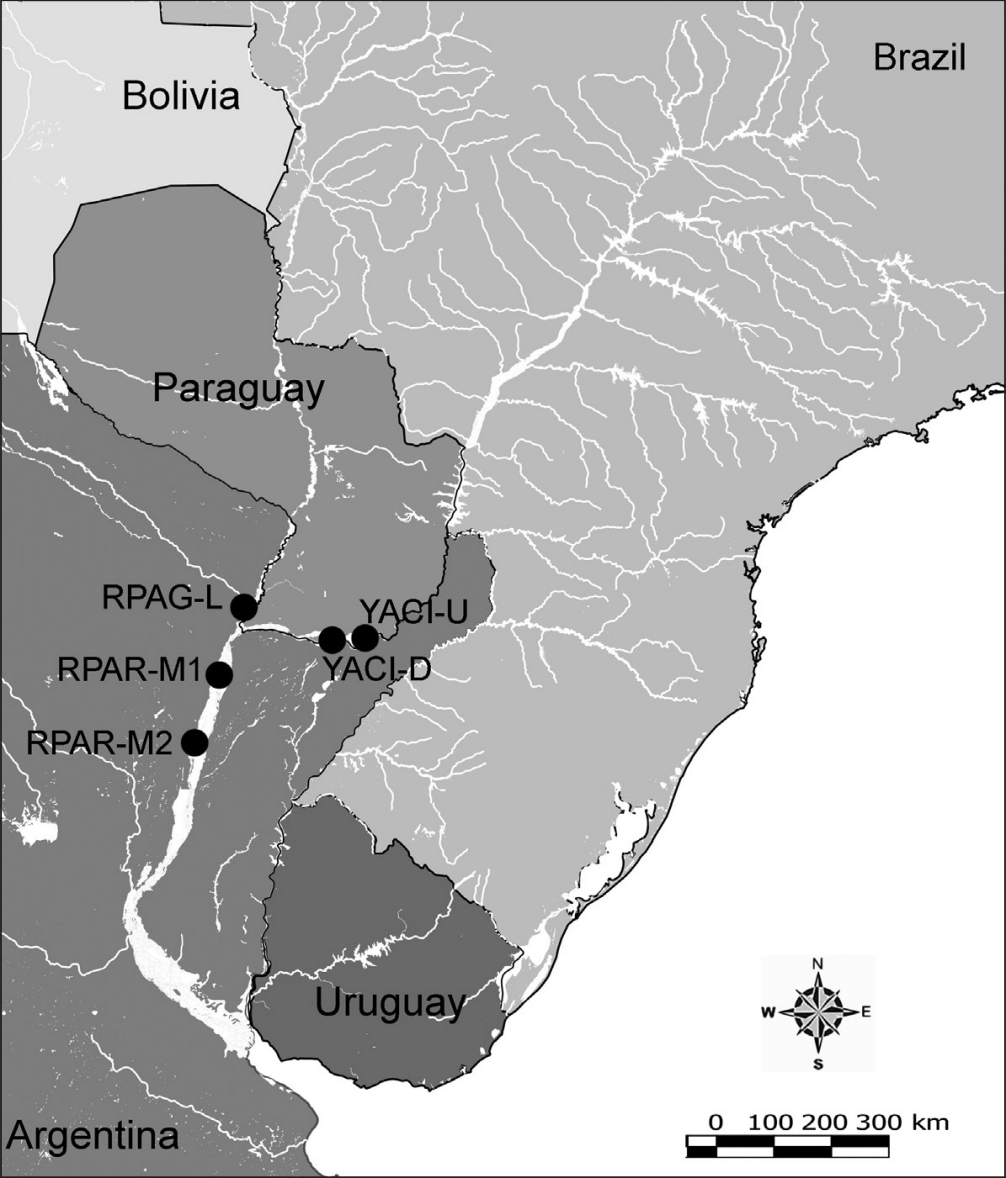
Geographical distribution of *Notodiaptomus isabelae* in de la Plata river basin.

The specimens were collected in the middle section of the Paraná River at Yaciretá Reservoir (Fig. 68, YACI-D). The type locality of this species is in the state of Pernambuco (northeastern Brazil) but this species is distributed across much of Brazil and Argentina, with its southernmost boundary in the middle region of the Paraná River. It does not seem to occur in the Iguaçu and Uruguay rivers, and the low winter temperatures in this region may be a factor limiting its distribution. Dussart and Frutos (1986) recorded this species in the middle section of the Paraná River.

This species can be easily distinguished from its congeners by the proximal processes on the internal margin of the male right BspP5 and by the shape of the wings and GS of females. A comparison with the description by Paggi (1976) revealed minor differences in the shape of segment 20 of the male A1R. No falciform process was found on this segment in our specimens, but this absence can occur in some populations, and a similar range of variability of this process has been noted for other species (Ringuelet and Martínez de Ferrato 1967, Paggi 1976).

##### Notodiaptomus santafesinus

Taxon classificationAnimaliaCalanoidaDiaptomidae

(Ringuelet & Martínez de Ferrato, 1967)

**Figure 69.**
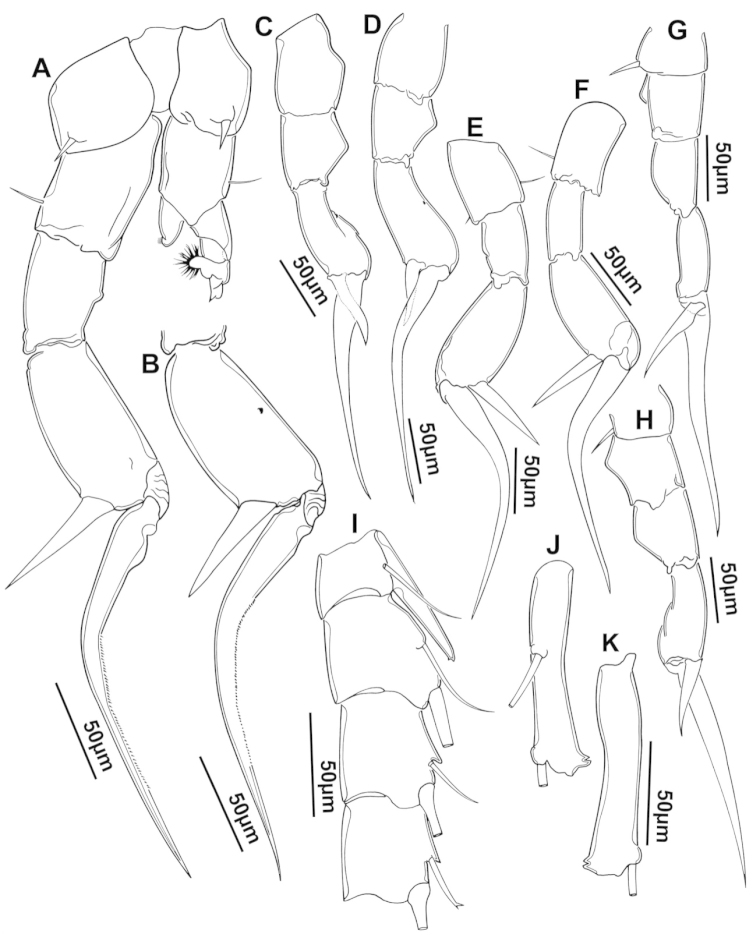
*Notodiaptomus santafesinus* male. **A** P5 **B** Right Exp2P5; **C–H** Different views of P5R **I** Segments 13–16 of A1R **J, K** Different views of segment 20 of A1R.

**Figure 70.**
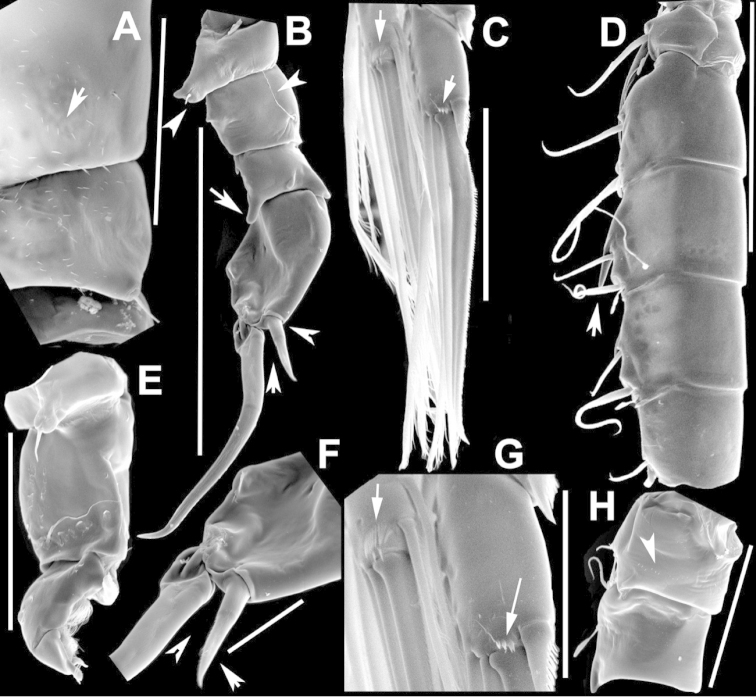
*Notodiaptomus santafesinus* male, SEM photographs. **A** Dorsal view of right side of Ped3, Ped4 and Ped5 (100 µm) **B** Right P5 (300 µm) **C** Terminal segments of EnpP4 and ExpP4 (100 µm) **D** Segments 13–17 of A1R (200 µm) **E** P5L (100 µm) **F** Detail of Exp2P5R (50 µm) **G** Detail of ornamentation of spinules of EnpP4 and ExpP4 (50 µm) **H** Segments 1 and 2 of A1R (100 µm).

**Figure 71.**
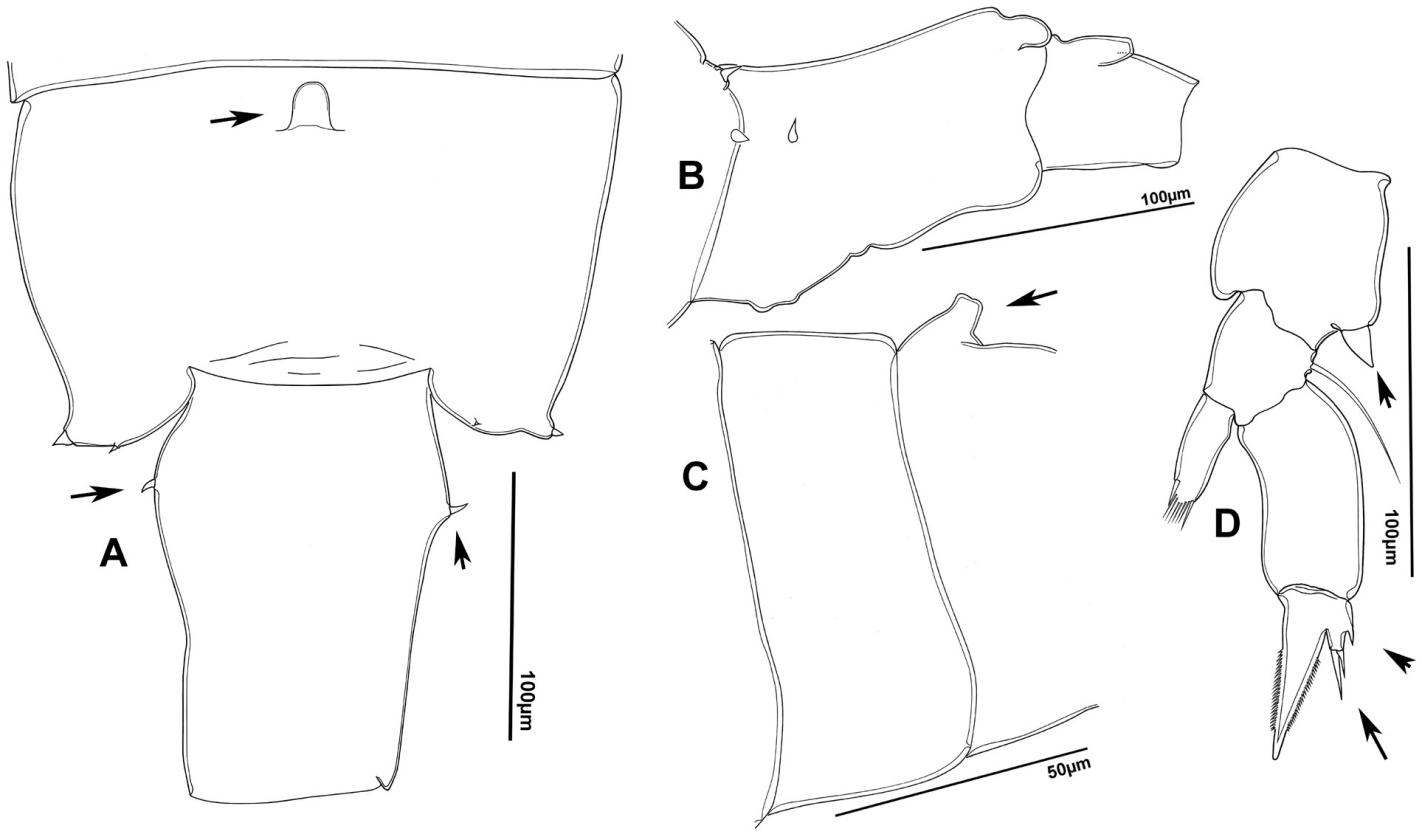
*Notodiaptomus santafesinus* female. **A** Dorsal posterior segments of prosome, with dorsal process, and GS **B** Lateral view of posterior margin of prosome, GS and urosome **C** Lateral view of prosome, showing dorsal process **D** P5.

**Figure 72.**
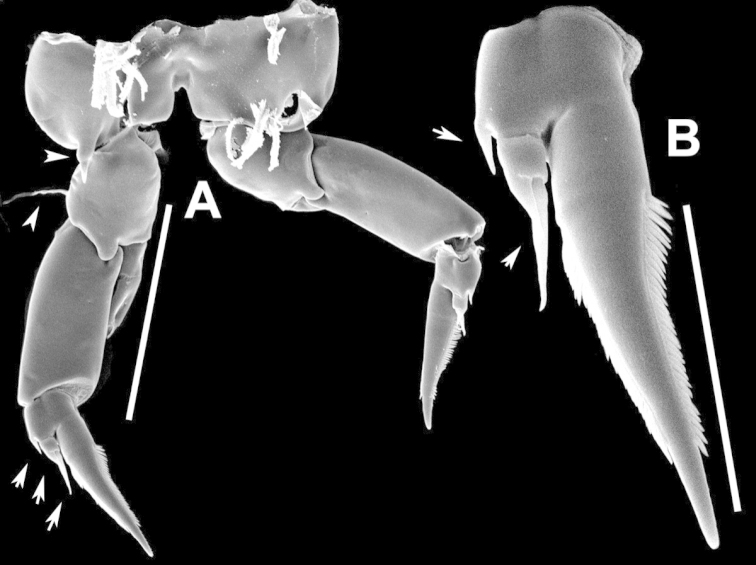
*Notodiaptomus santafesinus* female, SEM photographs. **A** P5 (100 µm) **B** Detail of Exp3P5 and terminal claw (50 µm).

**Figure 73.**
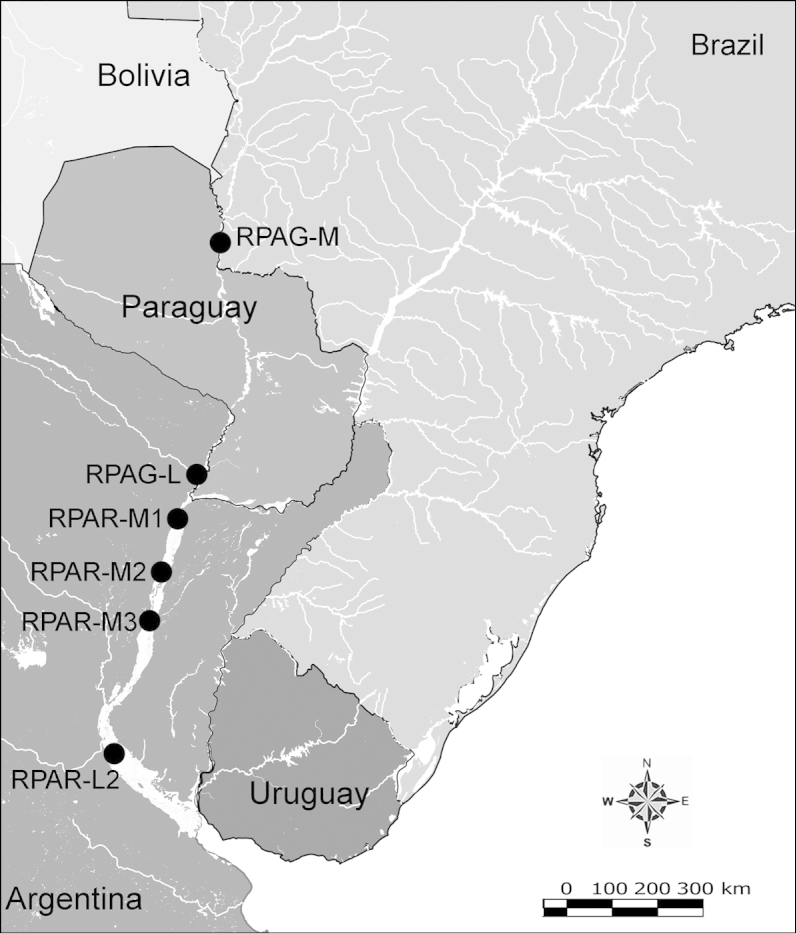
Geographical distribution of *Notodiaptomus santafesinus* in de la Plata river basin.

Figs 69 , 70 , 71 , 72 , 73

Diaptomus santafesinus Ringuelet & Martínez de Ferrato, 1967

###### Diagnosis.

**Adult male, body length 967 µm.** Dorsal and lateral surfaces of Ped3, Ped4 and Ped5 ornamented with scattered setules (Fig. 70A). Segment 1 of A1R with setule row (Fig. 70H); segment 13 with modified seta reaching beyond middle of segment 14; segments 15 and 16 each with small spinous processes (Figs 69I, 70D); distal margin of segment 20 of A1R terminating in small bifid process (Fig. 69J, K). Tips of rami of P4 ornamented with minute spinule combs (Fig. 70C, G). Left and right CxP5 each with conical process bearing sensilla at apex (Figs 69A, G, 70B, E). BspP5L with smooth inner surface, lacking spinulation or processes (Figs 69A, 70E). Right BspP5 with smooth inner surface, lacking spinulation or processes (Fig. 69A), outer seta short, less than half length of external margin Exp1P5; lateral spine on right Exp2P5 inserted close to insertion of terminal claw, strong and slightly outward curved, approximately four times longer than wide (Figs 69B–H, 70F); terminal claw with main curvature in proximal 1/3.

**Adult female, body length 1271 µm.** Incomplete suture present between Ped4 and Ped5; lacking rows of spinules on posterior margin of pedigers (Fig. 71A). Dorsal process present on midline of Ped4 (Fig. 71A, C); lateral wings slightly asymmetrical; both projections with pair of sensillae, one large and one small. GS slightly asymmetrical, about 1.8 to 1.9 times longer than wide; anterior part slightly dilated, with sensilla at apex of each swelling; left sensilla positioned slightly anterior to right; left sensilla directed slightly posteriorly, right sensilla directed slightly anteriorly. P5 symmetrical (Figs 71D, 72A) with small conical process at outer distal corner of CxP5, bearing sensilla approximately 1.5 times longer than wide; BspP5 with external seta of medium length, not exceeding length of internal margin of Exp3P5. EnpP5 unsegmented, reaching middle of inner margin of Exp1P5. Exp 3-segmented; lateral spine of Exp2P5 shorter than external margin of Exp3; external seta of Exp3 about half length of internal seta; internal seta almost reaching middle of terminal claw (Fig. 72B).

###### Remarks.

The specimens examined were collected in the lower stretch of the Paraguay River (RPAG–B). This species is found in Argentina in the middle and lower stretches of the Paraná River and it can be considered to be a common species in the zooplankton community of this region (Fig. 73). However, Ringuelet and Martínez de Ferrato (1967) found that this species was uncommon during the period of their study, occurring in lotic or adjacent systems only between February and April. It was not found in reservoirs. This species can be readily distinguished from its congeners because of the position of the robust lateral spine on the right Exp2P5, close to the insertion of the terminal claw.

##### Notodiaptomus spiniger

Taxon classificationAnimaliaCalanoidaDiaptomidae

(Brian, 1925)

**Figure 74.**
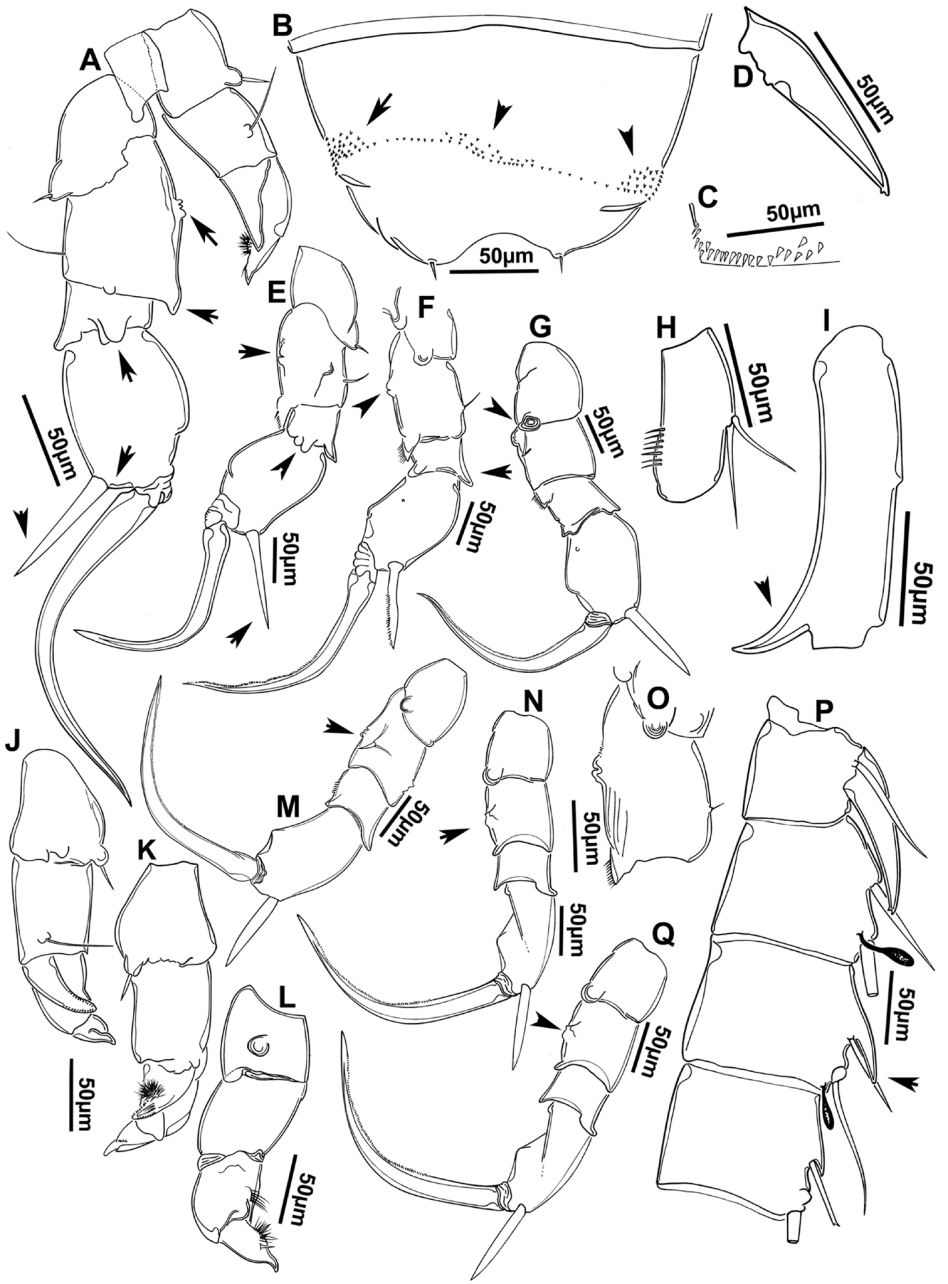
*Notodiaptomus spiniger* male. **A** Dorsal P5 **B** Ped3 and Ped4, showing details of spinular ornamentation **C** Details of spinules on corner of Ped4 **D** Spinous process of segment 13 of A1R **E–G** P5R **H** First segment of Enp of antenna 2 **I** Segment 20 of A1R **J–L** Different views of P5L **M, N** Different views of P5R **O** Detail of mammiform process on internal margin of right BspP5 **P** Segments 13–16 of A1R **Q** P5R.

**Figure 75.**
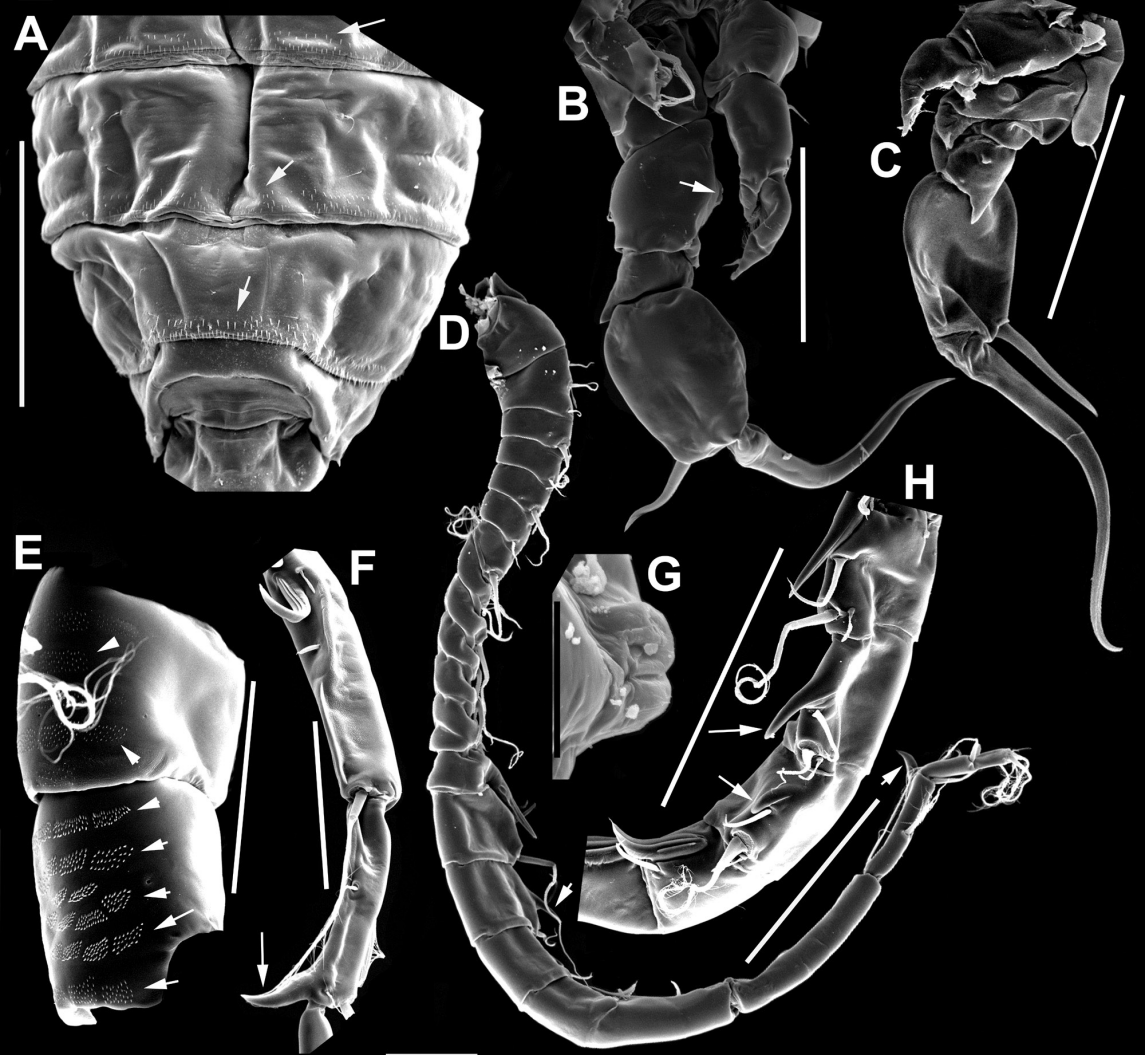
*Notodiaptomus spiniger* male, SEM photographs. **A** Dorsal prosome and urosome, showing details of spinular ornamentation (200 µm) **B, C** P5 (150 µm) **D** A1R (120 µm) **E** Dorsal urosome somites 3 and 4, showing details spinular ornamentation (100 µm) **F** Segments 19 and 20 of A1R (100 µm) **G** Mammiform process on internal margin of BspP5R (20 µm) **H** Spinous process on segment 13 of A1R, and segments 14–17 (200 µm).

**Figure 76.**
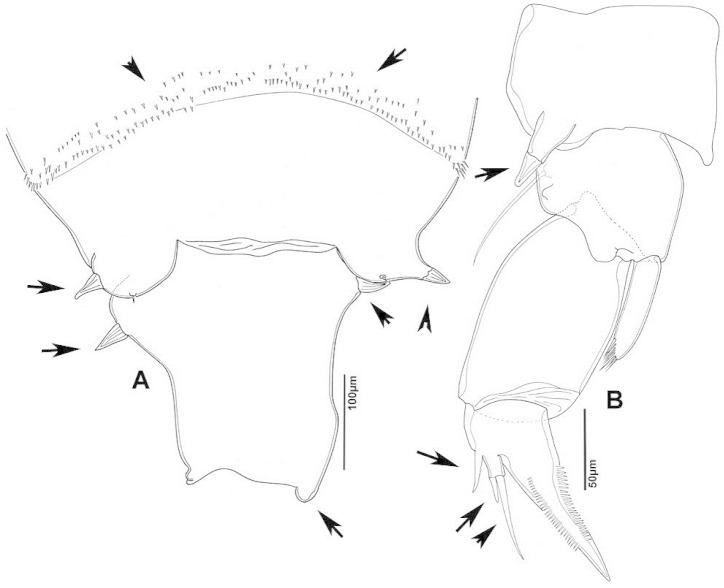
*Notodiaptomus spiniger* female. **A** Dorsal posterior pedigers, showing details of spinular ornamentation on dorsal surface, and GS **B** P5.

**Figure 77.**
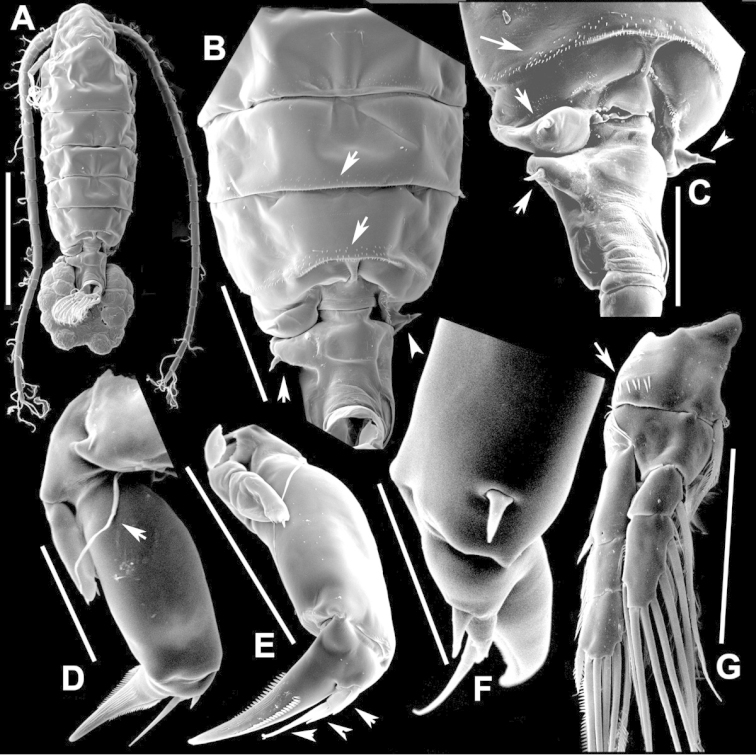
*Notodiaptomus spiniger* female, SEM photographs. **A** Dorsal view of adult (500 µm) **B** Posterior pedigers, showing details of spinule rows, and GS (150 µm) **C** Dorsal view of posterior pedigers showing details of spinule rows, and GS (150 µm) **D** P5L (50 µm) **E** P5L (100 µm) **F** ExpP5 (50 µm) **G** Left P1, showing details of rows of spinules on Cx (100 µm).

**Figure 78.**
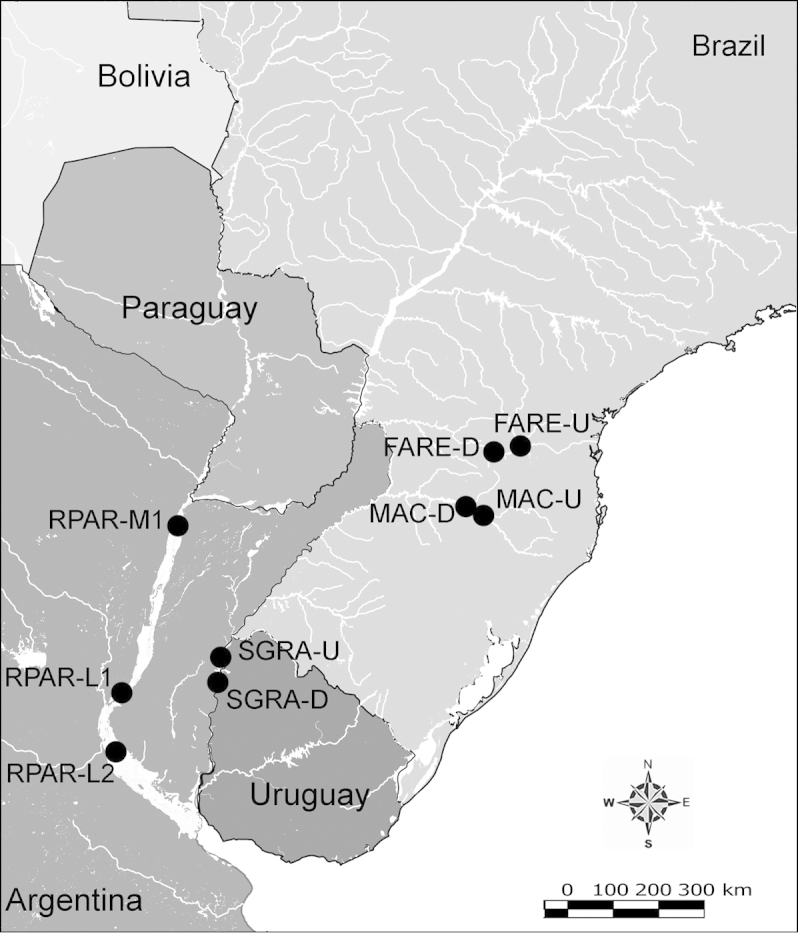
Geographical distribution of *Notodiaptomus spiniger* in de la Plata river basin.

Figs 74 , 75 , 76 , 77 , 78

Diaptomus spiniger Brian, 1925
Argyrodiaptomus spiniger (Brian, 1925)
Diaptomus toldti Pesta, 1927
Notodiaptomus orellanai Dussart, 1979

###### Diagnosis.

**Adult male, body length 1466 µm.** Patches of spinules present dorsally and laterally along suture between Ped3 and Ped4 (Figs 74B, C, 75A), and on the surface of Ur3 and Ur4 (Fig. 75E). Modified seta well developed on segment 13 (Fig. 74D, Q) and spinous process well developed on segment 15 of A1R (Figs 74Q, 75H); segment 20 of A1R typically produced into distal falciform process, longer than wide (Figs 74J, 75D, F), but process sometimes absent (see Dussart and Frutos 1985). End1 of A2 ornamented with spinule row, lacking pore (Fig. 74I). Rounded mammiform process present on internal margin of right BspP5 (Figs 74A, P, 75B, G). Left CxP5 1.5 times longer than wide; BspP5L about 1.2 longer than wide (Fig. 74K–M). Lateral spine of right Exp2P5 straight, almost as long as segment, inserted close to origin of terminal claw; terminal claw long, approximately 3 times as long as lateral spine (Figs 74F–H, N, O, R, 75C).

**Adult female, body length 1688 µm.** Complete suture present between Ped4 and Ped5; with several irregular rows of spinules present dorsally along posterior margin of Ped4 (Figs 76A, 77A–C); lateral wings slightly asymmetrical, left wing larger than right; both wings bearing pair of sensillae, one large and one small; large sensilla on left side located on hemispherical projection, right sensilla located on apex of wing (Fig. 77C). GS asymmetrical, about 1.5 times longer than wide; anterior part slightly dilated, with swelling on left side larger than on right, sensilla present on apex of each, both about twice as long as wide; sensilla on right side directed perpendicular to long axis of body (Fig. 76A). Right margin of GS longer than left. Cx of P1 with spinules on antero-lateral surface (Fig. 77G). P5 symmetrical (Fig. 76B) with small conical process at distal corner of CxP5, bearing triangular sensilla approximately 1.8 times longer than wide. BspP5 with outer seta of medium length, reaching middle of external margin of Exp1P5 (Fig. 77D). EnpP5 one-segmented, slightly longer than internal margin of Exp1P5. Exp 3-segmented; lateral spine of Exp2P5 long, reaching middle of external seta of Exp3P5 (Fig. 77E); internal seta of Exp3P5 about 3.5 times longer than external seta of Exp3P5; internal seta of Exp3P5 extending beyond middle of terminal claw.

###### Remarks.

The illustrated specimens were caught in the upper reaches of the Uruguay River, in the Machadinho Reservoir. The northernmost boundary of its distribution may lie in the northern sector of Paraná State (Brazil), its northernmost record is from the floodplain of the upper Paraná River (Fig. 78).

This species has been the subject of much taxonomic confusion. Ringuelet and Martínez de Ferrato (1967) argued that misinterpretation of the intraspecific variability of the spinous processes on segment 15 and of the falciform process on segment 20 of the male A1R is responsible for the establishment of “Diaptomus” toldti Pesta, 1927, a junior subjective synonym of *Notodiaptomus spiniger*. They added that individuals with well-developed spinous processes typically had a larger body size. Brehm (1933) included this species in the genus *Argyrodiaptomus*, but Kiefer (1936) doubted its inclusion in the genus because of its possession of a mammiform process on the internal margin of the right BspP5 and thus suggested an affinity with *Notodiaptomus*. Some subsequent authors (e.g. Ringuelet and Martínez de Ferrato 1967) have placed this species in “*Diaptomus*” as *Diaptomus spiniger*, while Dussart and Defaye (2002) treated it as *incertae sedis* within *Argyrodiaptomus*.

There are two other synonyms: “Diaptomus” birabeni Brehm, 1957 and *Notodiaptomus orellanai* Dussart, 1979 as proposed by Paggi in his MS Thesis in 1994. In each case the new species was established on the basis of morphological characters (Brehm 1957, Dussart 1979), but both synonymies are now widely accepted.

Further study of this species is necessary, including study of museum collections, in order to verify these synonymies and assess the evidence supporting the placement of this species in the genus *Notodiaptomus*, in part because of the presence of a mammiform process on the internal margin of the right BspP5, a character which conflicts with the current diagnosis of the genus.

#### Genus *Odontodiaptomus* Kiefer, 1936

##### Odontodiaptomus thomseni

Taxon classificationAnimaliaCalanoidaDiaptomidae

(Brehm, 1933)

**Figure 79.**
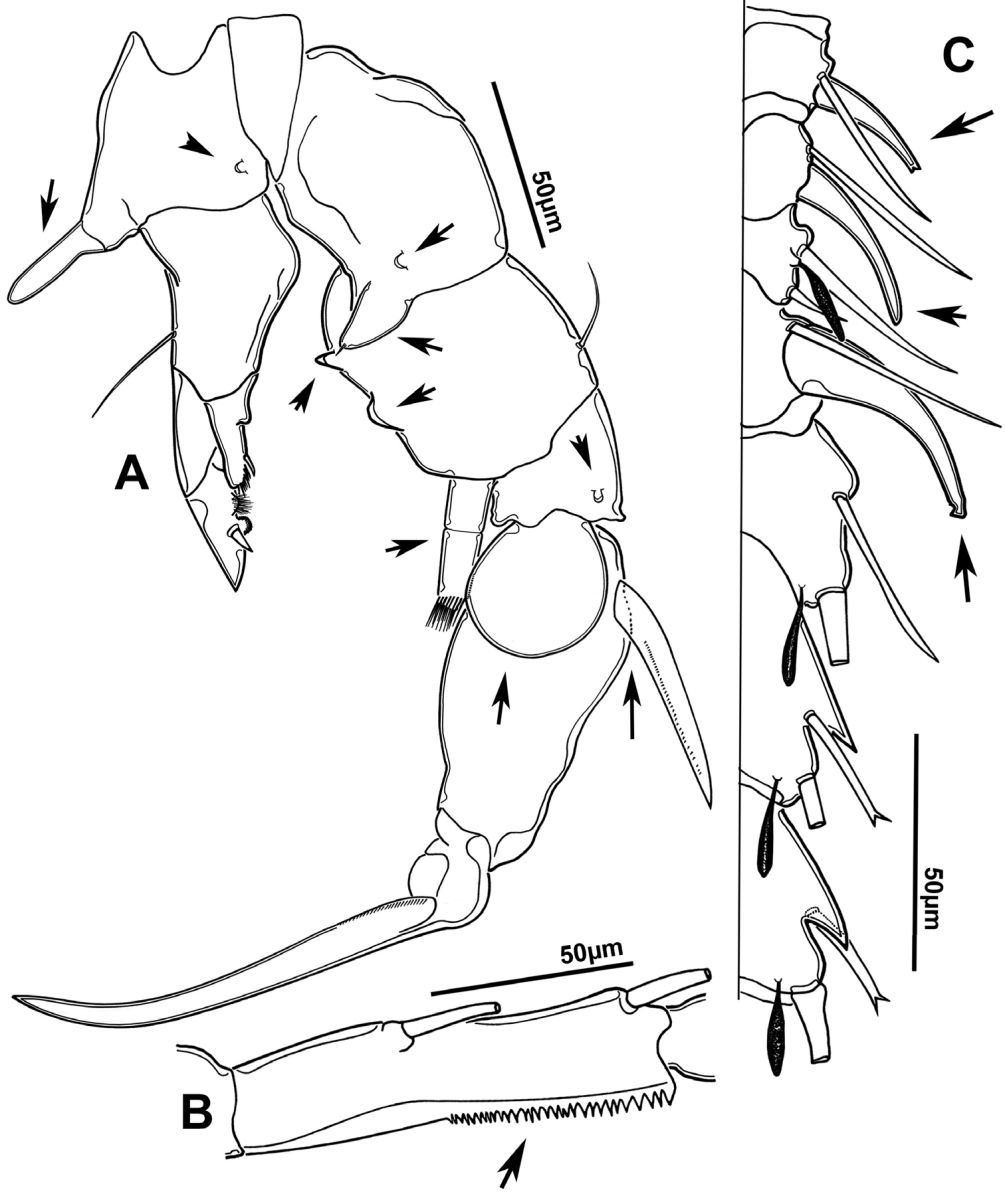
*Odontodiaptomus thomseni* male. **A** P5 in frontal view **B** Segment 20 of A1R **C** Segments 10–16 of A1R.

**Figure 80.**
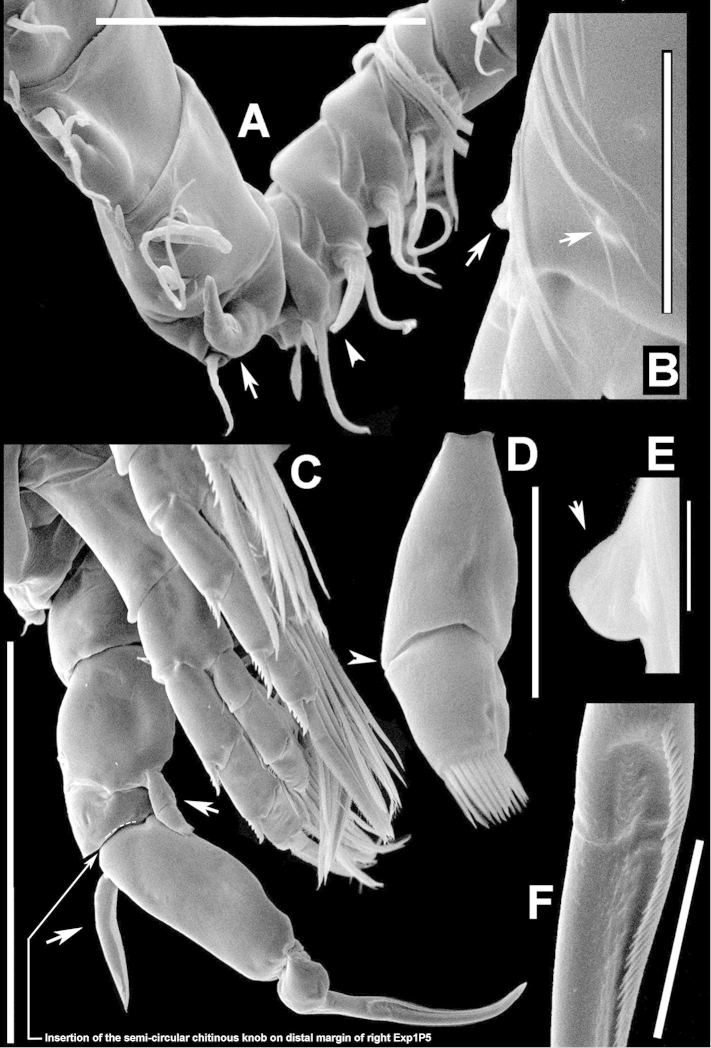
*Odontodiaptomus thomseni* male, SEM photographs. **A** Dorsal view of segments 11–16 of A1R (500 µm) **B** Detail of small chitinous knob on internal margin of left CR (20 µm) **C** P3, P4 and right P5 (caudal view), showing detail of EnpP5, lateral spine of P5 (200 µm) and were the semi-circular knob in inserted in Exp2P5 in frontal view **D** Right EnpP5 (2 µm) **E** Detail of chitinous knob on internal margin of CR (2 µm) **F** Detail of terminal claw (20 µm).

**Figure 81.**
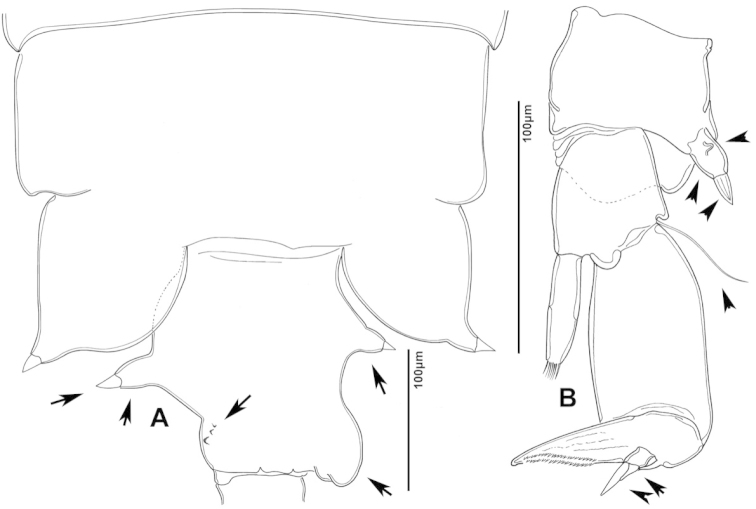
*Odontodiaptomus thomseni* female. **A** Posterior pedigers and GS **B** P5.

**Figure 82.**
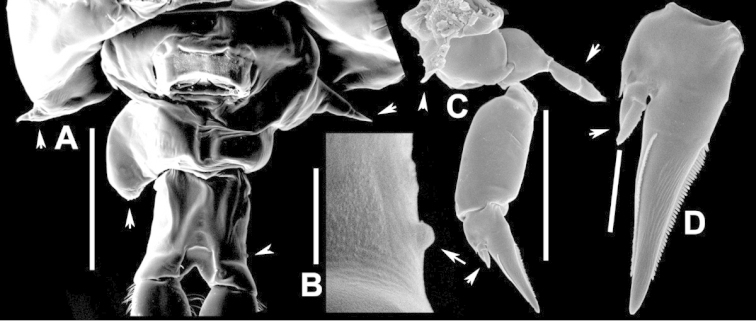
*Odontodiaptomus thomseni* female, SEM photographs. **A** Ventral view of posterior pedigers, GS, urosome and base of CR (100 µm) **B** Detail of chitinous knobs on left external margin of last somite of urosome (10 µm) **C** P5 (100 µm) **D** Exp3P5 and terminal claw (25 µm).

**Figure 83.**
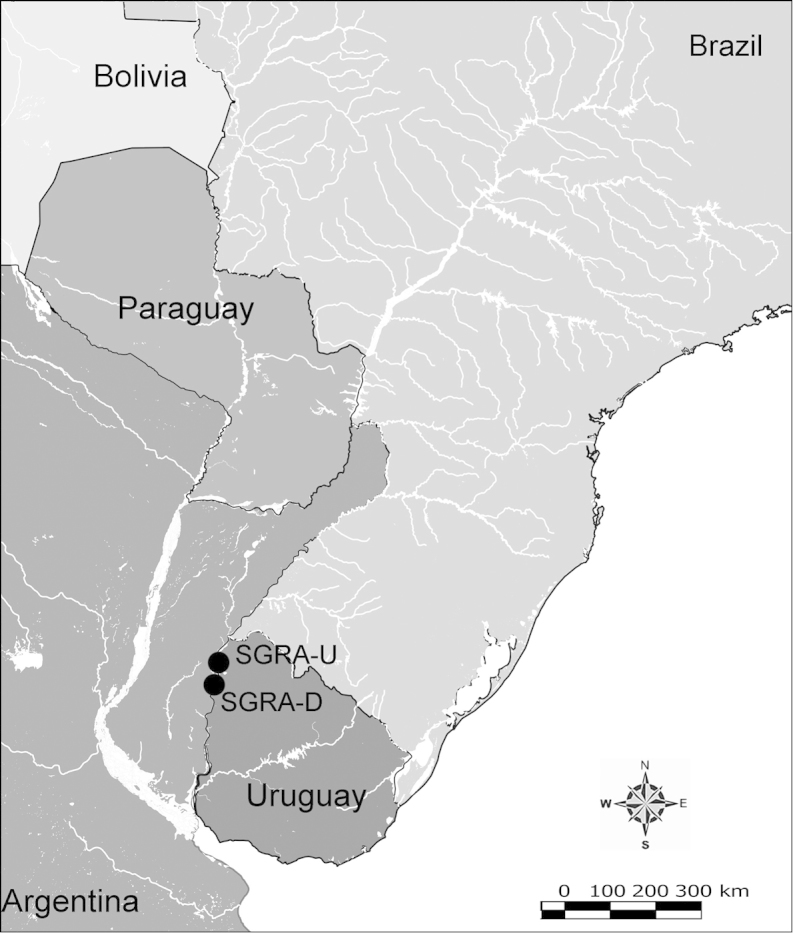
Geographical distribution of *Odontodiaptomus thomseni* in de la Plata river basin.

Figs 79 , 80 , 81 , 82 , 83

Diaptomus thomseni Brehm, 1933

###### Diagnosis.

**Adult male, body length 1088 µm.** Small chitinous knob present distally on internal margin of left CR, other small chitinous processes present on right CR (Fig. 80B, E). Modified setae on segments 10, 11 and 13 of A1R large (Figs 79C, 80A); segment 14 lacking spinous process; spinous processes on segments 15 and 16 well developed; segment 20 of A1R bearing dentate hyaline membrane (Fig. 79B). Right CxP5 with small chitinous knob on surface in middle of segment (Fig. 79A). Right BspP5 bearing three rounded chitinous projections on internal margin. Right EnpP5 2-segmented (Figs 79A, 80D). Right Exp1P5 with semi-circular chitinous knob on distal margin, visible in anterior view; right Exp2P5 with lateral spine inserted proximally (Figs 79A, 80C), about 6 to 7 times longer than wide. Terminal claw of right Exp2P5 about 2.3 times longer than lateral spine. CxP5L with distal outer projection bearing sensilla about 3 times longer than wide (Fig. 79A); well-developed outer seta present on BspP5L.

**Adult female, body length 1245 µm.** Incomplete suture present between Ped4 and Ped5; surface of Ped4 and Ped5 smooth, lacking rows of spinules dorsally along posterior margin (Fig. 81A); lateral wings slightly asymmetrical, similar in size; left sensilla directed posteriorly, right sensilla perpendicular to body axis (Fig. 82A). GS asymmetrical, about 1.6 times longer than wide; anterior part slightly dilated, with swelling on right side anterior to that on left; right side margin with large rounded process at posterior end (Fig. 81A). Ur2 with small chitinous process on outer margin on left side (Fig. 82B). P5 symmetrical (Fig. 81B) with small conical process at outer distal corner of CxP5, bearing short robust sensilla; BspP5 with short outer seta, about as long as EnpP5. EnpP5 one-segmented. ExpP5 3-segmented; lateral spine of Exp2P5 exceeding external margin of Exp3P5; external seta of Exp3P5 nearly 2/3 length of internal seta; internal seta robust, only about 1/5 length of terminal claw (Fig. 82C, D).

###### Remarks.

The specimens were collected in the low stretch of the Uruguay River at Salto Grande Reservoir (Fig. 83, SGRA-D). Only three individuals were found and a full re-description was published by Perbiche-Neves et al. (2012). This species is on the red list of endangered species (IUCN–Red List of Threatened Species, 2010–Reid 1996), due to lack of recent records. The only previous confirmed record of this species was its original description (Brehm 1933). There is a subsequent record of this species from pools in Venezuela, but it is doubtful and requires confirmation (Perbiche-Neves et al. 2012). Ringuelet (1958) did not find this species in his work on Argentinian copepods, nor did Paggi and José de Paggi (1990). This was only the second confirmed record of this rare species in 77 years.

The genus *Odontodiaptomus* comprises three species (equivalent to the *thomseni* group of Wright 1936), and there is a great interest in the genus because of its apparent isolation from other Neotropical Diaptomidae (Santos-Silva 2008). A second species of this genus, *Odontodiaptomus paulistanus* (Wright, 1936) is relatively common in Brazil, but was not found in the present study. The third species, *Odontodiaptomus michaelseni* (Mrázek, 1901), was not found in our study.

#### Genus *Diaptomus* Westwood, 1836

##### “Diaptomus” curvatus

Taxon classificationAnimaliaCalanoidaDiaptomidae

Perbiche-Neves, Boxshall & Paggi, 2013

**Figure 84.**
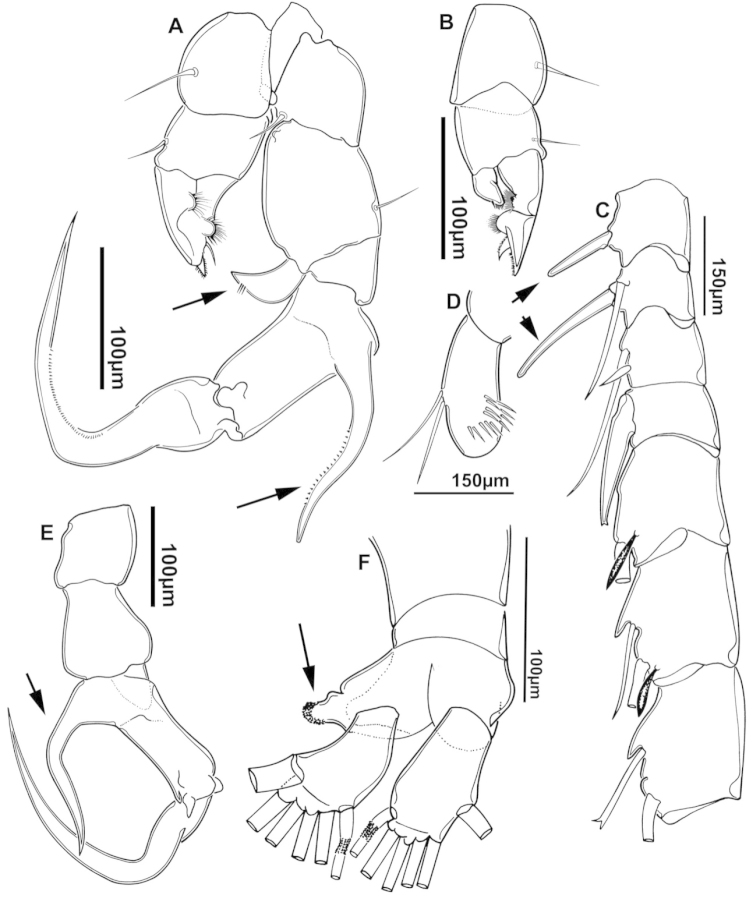
“Diaptomus” curvatus male. **A** P5 **B** Left P5 **C** Segments 10–16 of A1R **D** First segment of Enp of antenna 2 **E** Right P5 **F** Urosome somites 2–4 and CR, showing dorsal process on Ur4 (arrowed).

**Figure 85.**
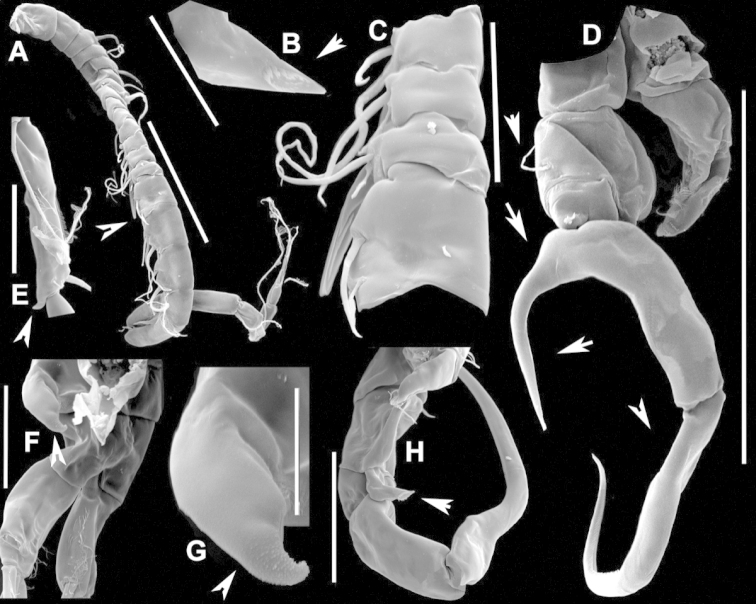
“Diaptomus” curvatus male, SEM photographs. **A** A1R (150 µm) **B** Detail of endopod of P5R (25 µm) **C** Segments 11–14 of A1R (50 µm) **D** P5 (200 µm) **E** Segment 20 of A1R (50 µm) **F** End of urosome and CR, showing detail of dorsal process (50 µm) **G** Detail of dorsal process on Ur4 (20 µm) **H** Right P5 (100 µm).

**Figure 86.**
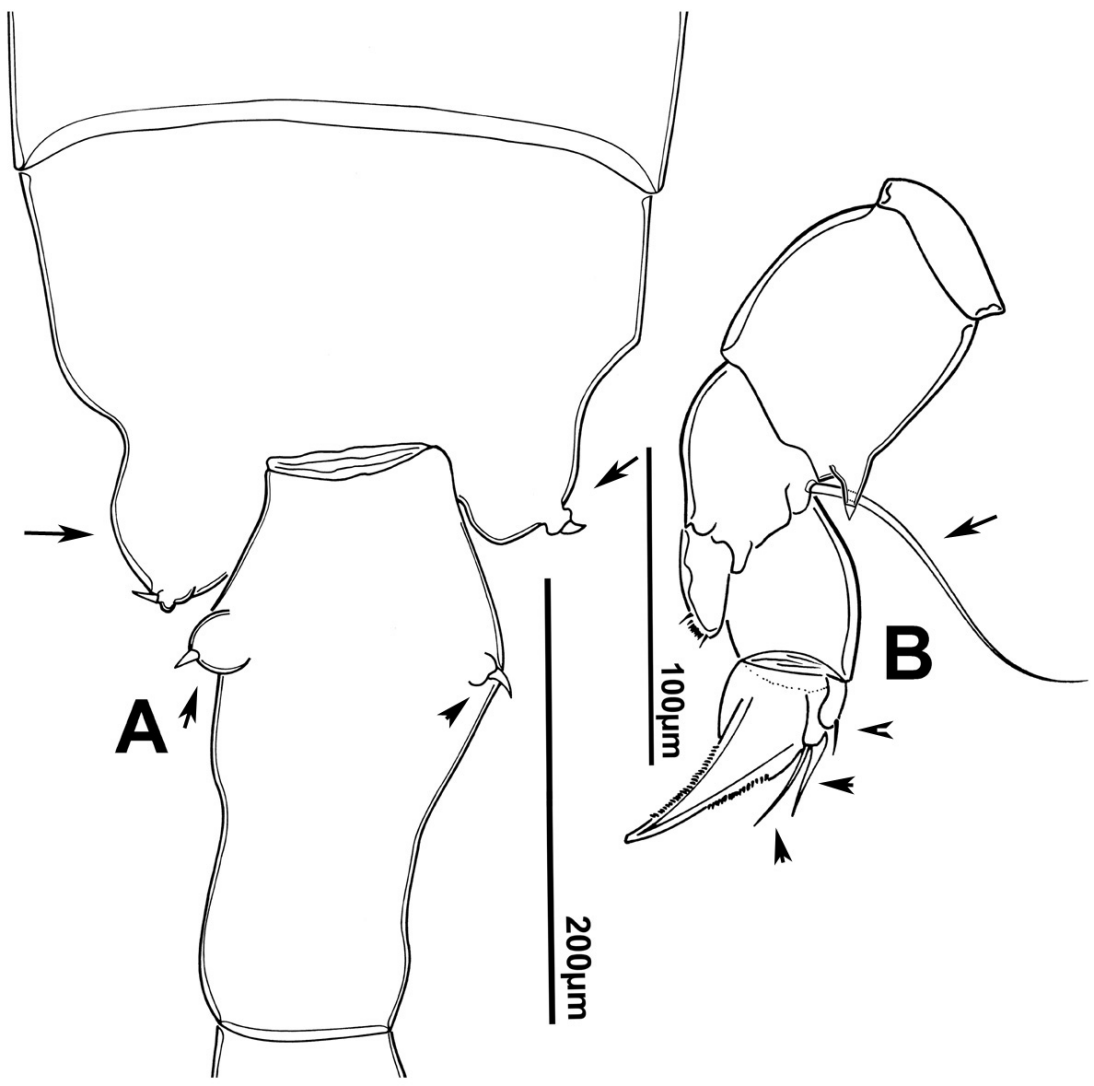
“Diaptomus” curvatus female. **A** Posterior pedigers and GS **B** P5.

**Figure 87.**
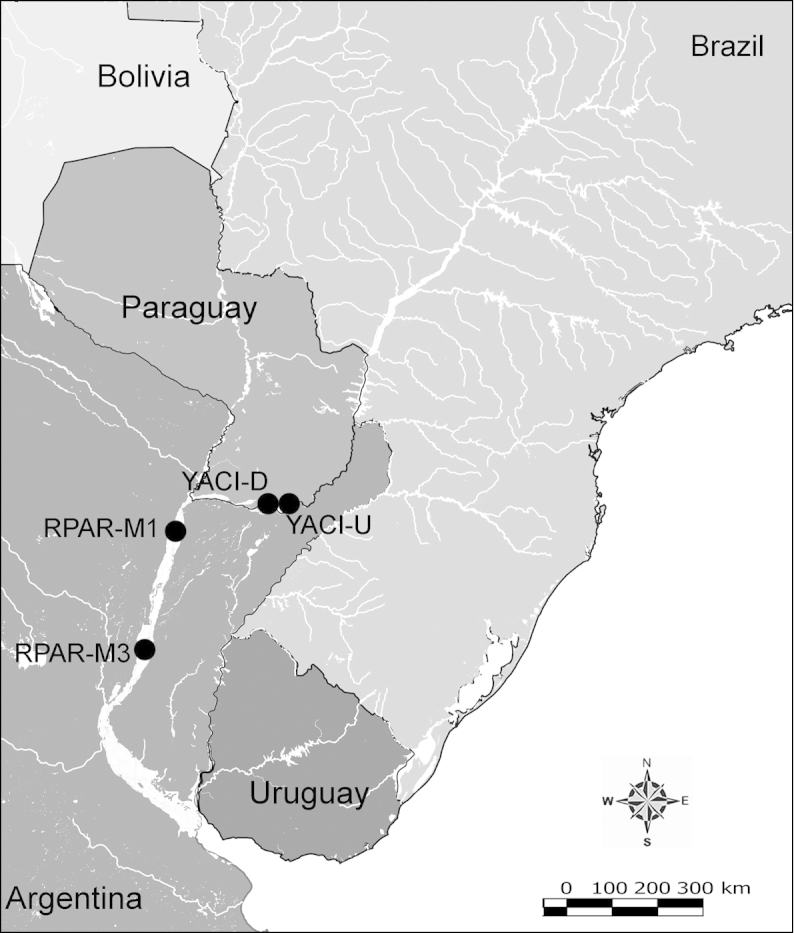
Geographical distribution of *Diaptomus curvatus* in de la Plata river basin.

Figs 84 , 85 , 86 , 87

“Diaptomus” curvatus Perbiche-Neves, Boxshall & Paggi, 2013 in Perbiche-Neves, Boxshall, Paggi, Rocha, Previattelli & Nogueira, 2013

###### Diagnosis.

**Adult male, body length 923 µm.** Ur4 with triangular-shaped dorsal process ornamented with small granulations at left distal corner (Figs 84F, 85F, G), length similar to width of Ur4. CR asymmetrical, left ramus larger than right, internal seta of CR with narrow section close to base (Fig. 84F). Segment 11 of A1R with modified seta on anterior margin longer than modified seta on segment 13; spinous processes present on segments 15 and 16 (Figs 84C, 85A, C); segment 20 with small, curved, distal process (Fig. 85E). Two rows of spinules present on surface of Enp1 of A2 (Fig. 84D). Small mammiform process present proximally on internal margin of left and right BspP5 (Fig. 84A); lateral spine on right Exp2P5 strong, inserted proximally, curved in midsection, about 2/3 length of terminal claw (Figs 84A, E, 85D); base of terminal claw thick, almost same width as thinnest part of Exp2P5. Right EnpP5 tapering distally, ornamented with spinules subapically (Figs 84A, 85B). Lateral seta of CxP5L as long as width of segment; BspP5L as wide as long (Fig. 84B).

**Adult female, body length 1120 µm.** Incomplete suture present between Ped4 and Ped5; dorsal surface smooth, without spinules (Fig. 86A); lateral wings asymmetrical, left wing larger than right. GS slender, asymmetrical, about 1.6 times longer than wide; anterior part slightly dilated, with swelling on left side larger than right. P5 symmetrical (Fig. 86B) with small conical process at distal corner of CxP5, bearing short, triangular sensilla. BspP5 with long external seta, extending beyond base of terminal claw. EnpP5 one-segmented. ExpP5 3-segmented; lateral spine of Exp2 longer than external margin of Exp3P5; external seta of Exp3P5 about two thirds length of internal seta; internal seta reaching mid-length of terminal claw.

###### Remarks.

Several individuals of this species were found in the Yaciretá Reservoir (Fig. 87, YACI-B) in the middle Paraná River. This species had been found by S. M. Frutos (pers. comm.) in Yaciretá Reservoir, and J. C. Paggi (pers. comm.) found three males in the middle section of the Paraná River in 1971 and in 1993. This species is distinct from all other Diaptomidae found in South America, especially with regard to the P5 and the dorsal process on the Ur4. A preliminary phylogenetic analysis based on morphological characters was inconclusive with regard to which genus this species should be attributed to, thus suggesting the possibility that it represents a new genus. However, Perbiche-Neves et al. (2013) decided to maintain the status as “*Diaptomus*” *sensu lato* until a comprehensive phylogenetic analysis of Neotropical diaptomids could be completed.

##### “Diaptomus” frutosae

Taxon classificationAnimaliaCalanoidaDiaptomidae

Perbiche-Neves & Boxshall, 2013

**Figure 88.**
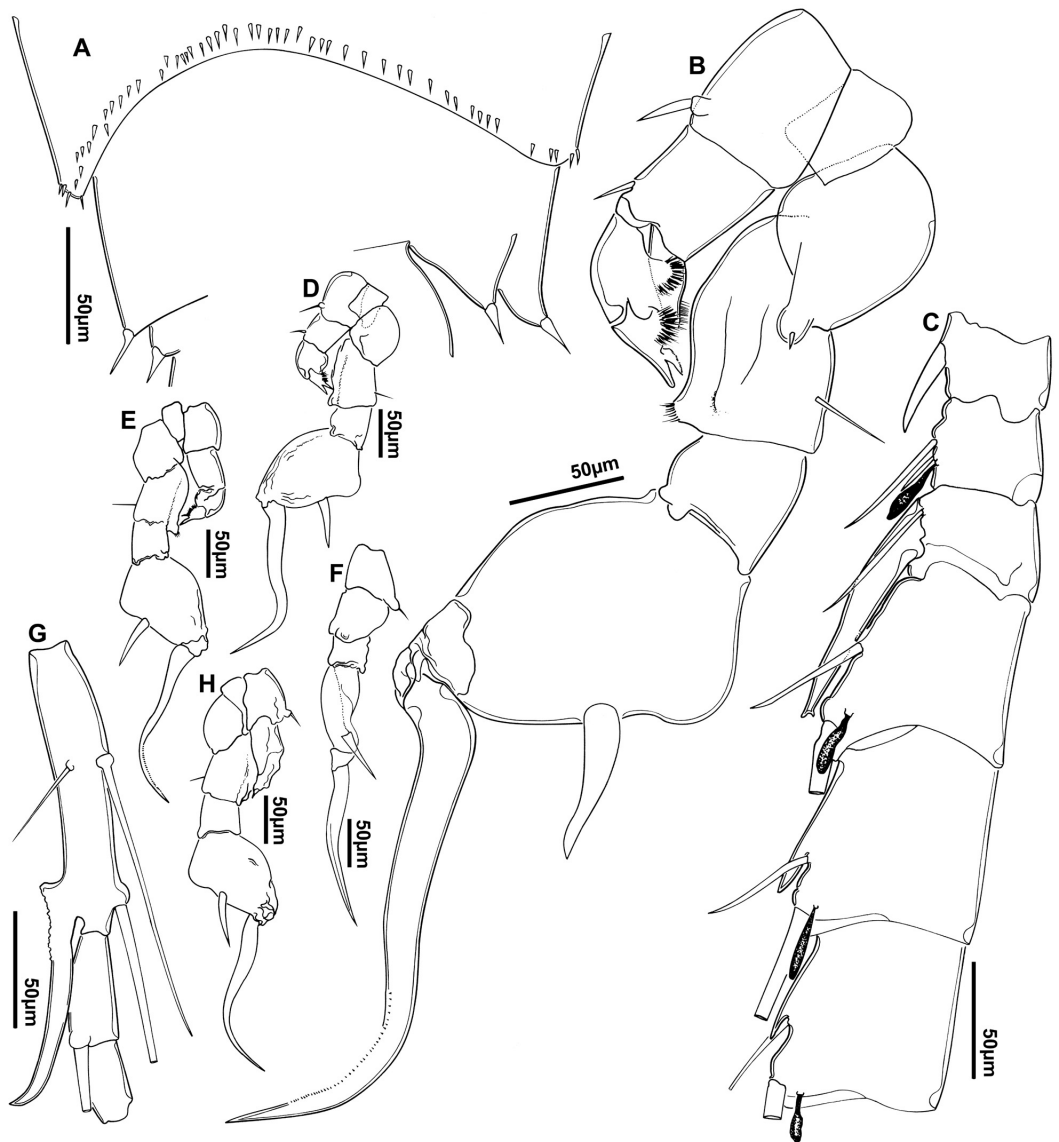
“Diaptomus” frutosae male. **A** Ped4 and Ped5, showing details of spinule rows **B** P5 **C** Segments 11–16 of A1R **D–F** Different views of P5 **G** Segment 20 of A1R, showing falciform process **H** P5.

**Figure 89.**
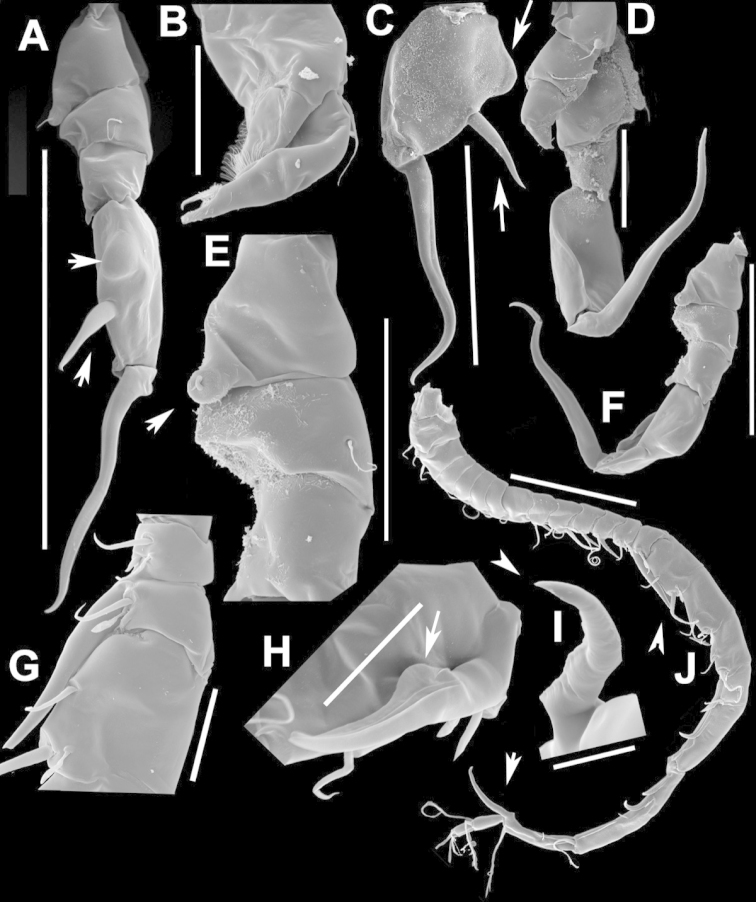
“Diaptomus” frutosae male, SEM photographs. **A** P5R, caudal view (300 µm) **B** P5L (50 µm) **C** Last segment of ExpP5L (200 µm) **D** P5 (100 µm) **E** Cx and Bsp of right P5 (100 µm) **F** Right P5, lateral view (200 µm) **G** Segments 12–14 of A1R (50 µm) **H** Segments 13 and 14, showing details of spinous process on segment 13 (50 µm) **I** Segment 20 of A1R (20 µm) **J** A1R (200 µm).

**Figure 90.**
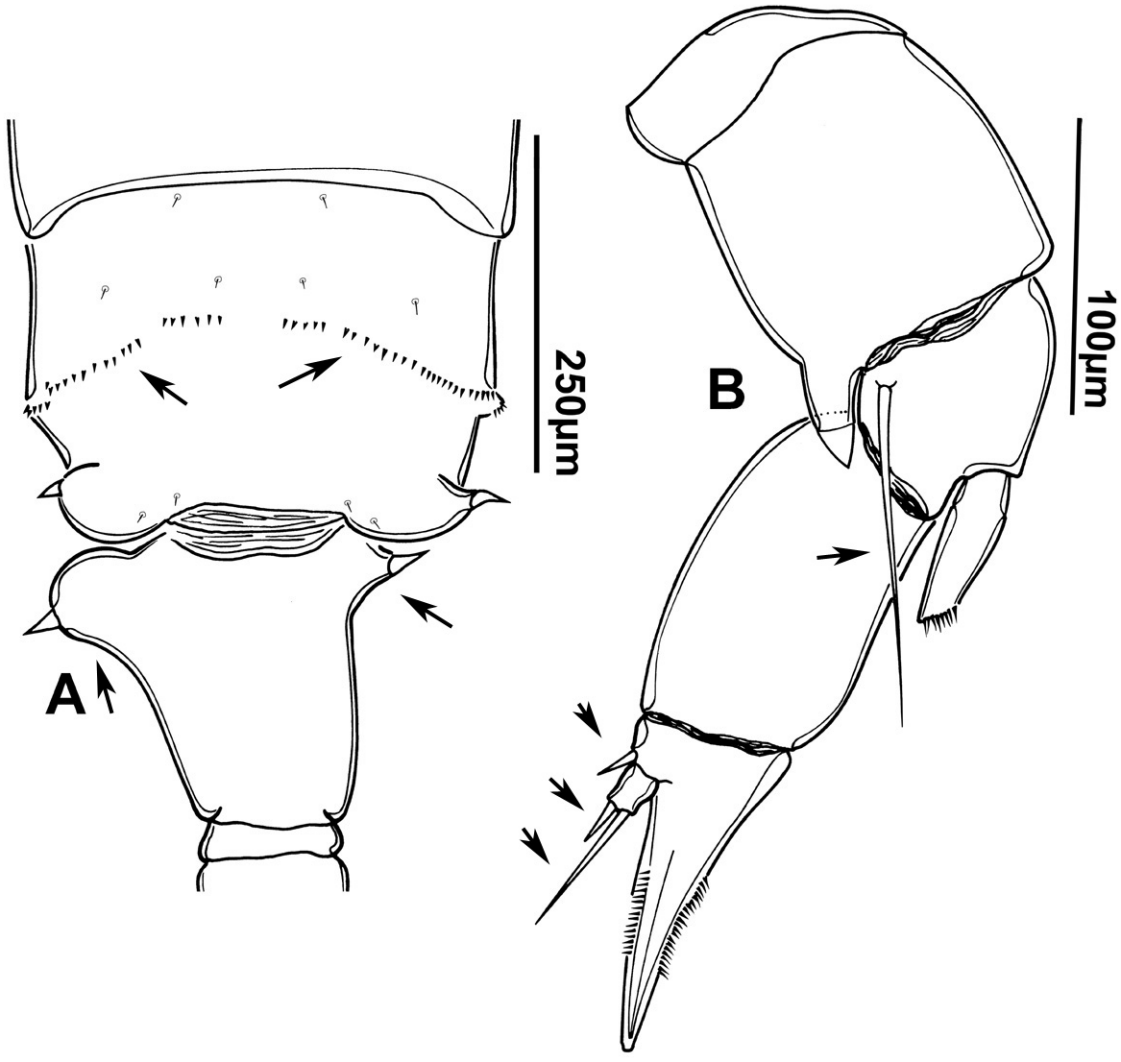
“Diaptomus” frutosae female. **A** Posterior pedigers and GS **B** P5.

**Figure 91.**
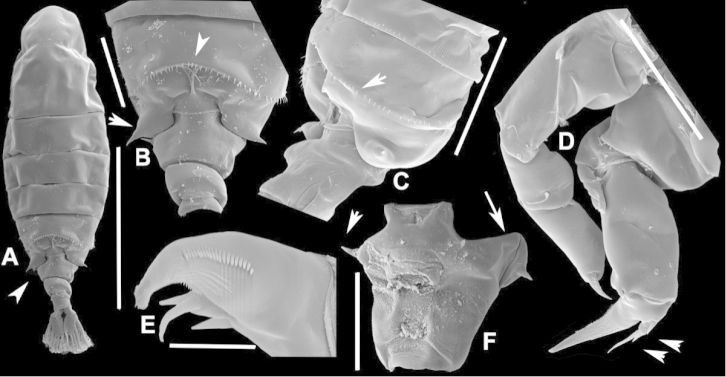
“Diaptomus” frutosae female, SEM photographs. **A** Dorsal view of female (500 µm) **B, C** Posterior pedigers, GS and urosome, in dorsal view, showing details of spinular ornamentation along posterior margin of Ped4 (**B** = 100 µm, **C** = 200 µm) **D** P5 (100 µm) **E** Exp3P5 and terminal claw (20 µm) **F** GS, ventral view (100 µm).

**Figure 92.**
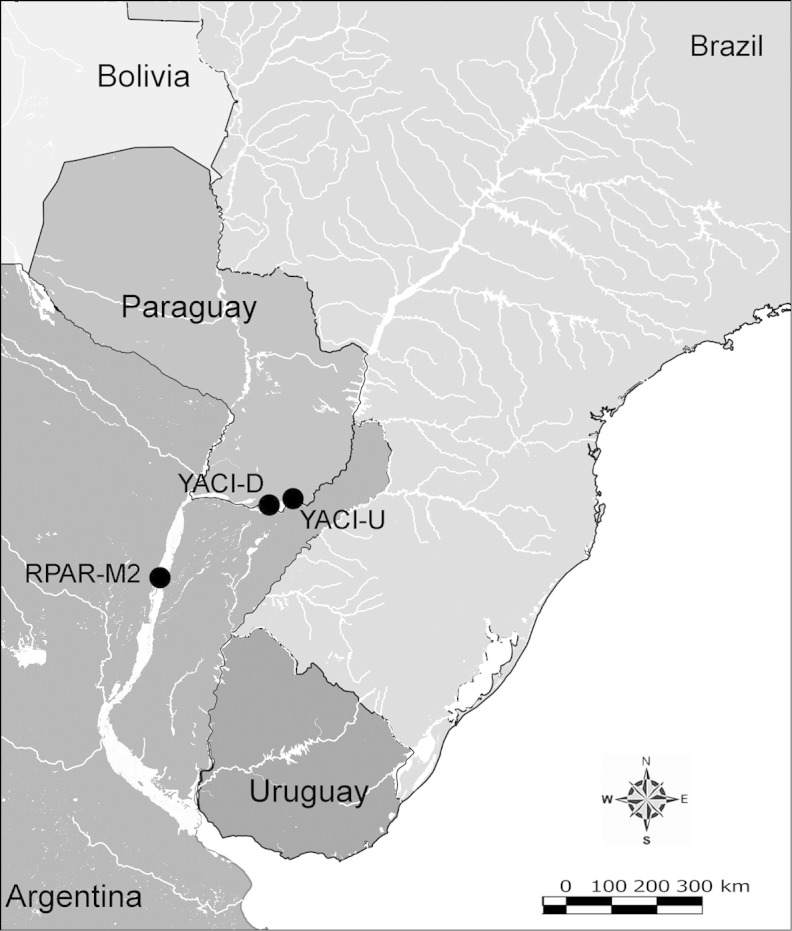
Geographical distribution of *Diaptomus frutosae* in de la Plata river basin.

Figs 88 , 89 , 90 , 91 , 92

“Diaptomus” frutosae Perbiche-Neves & Boxshall, 2013 in Perbiche-Neves, Boxshall, Paggi, Rocha, Previattelli & Nogueira, 2013

###### Diagnosis.

**Adult male, body length 1292 µm.** Irregular row of spinules present dorsally and laterally along posterior margin of Ped4 (Fig. 88A). Modified seta on segments 11 and 13 well developed, that on segment 13 longest, extending to end of segment 14 (Fig. 89G, H); spinous process well developed on segments 15 and 16 of A1R; spinous process on segment 15 longer than that on segment 16; falciform process on segment 20 of A1R in all specimens analysed (N=16), reaching beyond middle of apical segment, proximal surface of process rugose (Fig. 88G, 89I, J). Right CxP5 with well-developed distal process bearing short sensilla (Fig. 89E). Right Exp1P5 short, about 1.5 times wide than long; right Exp2P5 flattened, subtriangular in shape (Fig. 89C–D), wider than long, with swollen outer margin proximal to insertion of outer seta (Fig. 89A, C, 88B); outer seta slightly curved (Fig. 88B, 89C). Terminal claw strongly curved near tip (Figs 88D–F, H, 89F). Strong seta present on distal margin of CxP5L.

**Adult female, body length 1346 µm.** Incomplete suture present between Ped4 and Ped5; rows of strong spinules marking position of posterior margin of Ped4 (Figs 90A, 91B, C); lateral wings slightly asymmetrical, similar in size; both wings with sensilla at distal tip (Fig. 91A). GS symmetrical, approximately 1.8 times longer than wide; anterior part dilated, with swelling on left side larger than on right; sensilla on apex of each, about 1.8 times longer than wide; swelling on left side hemispherical, swelling on right more conical (Figs 90A, 91F). P5 symmetrical (Fig. 90B), with small conical process at outer distal corner of CxP5, bearing short, strong, triangular sensilla, about 1.5 times longer than wide. BspP5 with long outer seta, about twice length of EnpP5. EnpP5 one-segmented. Exp 3-segmented; lateral spine of Exp2 as long as external margin of Exp3 (Fig. 91D); external seta of Exp3 reaching one third length of internal seta; internal seta almost reaching mid-length of terminal claw; terminal claw with comb of robust spinules in mid-section (Fig. 91E).

###### Remarks.

The specimens were found in the middle part of the Paraná River (Fig. 92, RPAR-M2) and in the Yaciretá Reservoir (YACI-D). The species was relatively abundant in the samples and occurs in reservoirs and other water bodies near these locations, according to S. M. Frutos (pers. comm.). In a previous phylogenetic analysis, this species was placed close to the genus *Scolodiaptomus* Reid, 1987, which is monospecific [*Scolodiaptomus corderoi* (Wright, 1936)], because they share important features such as the subtriangular shape of Exp2P5R and the presence of modified setae or spinous processes on segments 13, 15 and 16 of the male A1R. However, it is very unlikely that “Diaptomus” curvatus belongs to the genus *Scolodiaptomus*, which is characterised by an unornamented hyaline lamella on segment 20 of A1R and by the presence of a dorsal cylindrical process on the Ped3. Again, the affinities of this species need to be tested as part of a comprehensive phylogenetic analysis of Neotropical diaptomids. It is likely that this species could be assignable to a new genus.

## Discussion

Nineteen species of diaptomid copepods were found during the present survey, but this figure probably is markedly lower than the estimated total for de la Plata river basin. The reason for this discrepancy may be related to sample representativeness due to limitations in terms of seasonality and kind of sampled freshwater habitats (only large rivers and reservoirs). Historical records suggest that there are at least another 15 species to be found in the basin. Boxshall and Defaye (2008) summarized published reports for the entire Neotropics and calculated a total of 82 species of Diaptomidae from the region. The 19 species found in this survey represent about 24% of that total. Despite this limitation, our sampling effort generated sufficient data to significantly improve our understanding of the spatial distribution of diaptomids throughout de la Plata river basin. Together with the existing literature, we can now begin to understand the large scale distribution patterns of most diaptomid species in the Neotropics. Andean diaptomids like the genus *Tumeodiaptomus* Dussart, 1979 were not found. Only three species, *Notodiaptomus coniferoides*, *Notodiaptomus conifer* and *Notodiaptomus isabelae* were distributed throughout the whole basin. However, detailed taxonomic studies are still required to investigate whether these morphological species represent complexes of cryptic species with limited distributional ranges, as is possibly the case at least for *Notodiaptomus coniferoides*. *Notodiaptomus coniferoides* occurred only in lotic stretches and it is likely that it does not occur frequently in reservoirs. *Notodiaptomus simmilimus* is another species that requires further detailed analysis.

There was no gradual decrease in the number of species towards the south of the continent, as might be inferred from Jablonski et al. (2006), for example, at least as far as the southern boundary of de la Plata basin. The central valley of the Paraná River serves as a route for the dispersal of several species of copepods. In its middle section it is possible to find species both from the northern group (e.g. from the *nordestinus* complex) and from the southern group (Argentinian species). There is evidence of the co-occurrence of members of the northern and southern groups together with other species which exhibit restricted distributions and a high degree of endemism. At sample site RPAR-L2 in the mid Paraná River and in Yaciretá Reservoir, for example, we observed the largest number of calanoids per sample anywhere in the basin. Some previous studies have mentioned the large number of species found in the Paraná River (Paggi and José de Paggi 1990, Dussart and Frutos 1985, 1986, Lansac-Toha et al. 2004, 2009).

With the exception of the most widespread species, *Notodiaptomus coniferoides*, *Notodiaptomus conifer* and *Notodiaptomus isabelae*, the other diaptomids could be roughly divided into three groups according to their geographical distribution patterns, and their relationship to limnological variables and climatic factors (Perbiche-Neves et al. 2014). Calanoids in the northern basin (especially the *nordestinus* complex, see Santos-Silva 2000) had a southern limit at the high/middle stretch of the central channel of the Paraná River, in the Iguaçu River, and in the upper Uruguay River. South of this region, there were species typical of the Argentinian fauna in the middle and lower basin, but in the middle section it is possible to find species from the northern and southern parts of the basin, as well as other species restricted to this particular range.

As emphasized in the diagnoses of species given above, the detailed analysis of segment 20 of the male A1R revealed substantial variation in the shape and state of development of the distal process of this segment: a falciform process is usually present but other shapes may occur, and many species lack a process on this segment. It is necessary to be cautious in interpreting the wide variability in this process exhibited by species of some diaptomid genera. Based on our observations, it should not be used alone or as a strict diagnostic character for the identity of a particular taxon. Paggi (1976) highlighted this morphological variation in diaptomids of the middle Paraná River region in Argentina. Similar patterns of variation have also been observed in species of *Argyrodiaptomus*, for example, *Argyrodiaptomus azevedoi* and *Argyrodiaptomus denticulatus*, as well as in the *Notodiaptomus* species, *Notodiaptomus spiniger*, *Notodiaptomus henseni*, and *Notodiaptomus isabelae*. Species of the genus *Argyrodiaptomus* typically have a falciform process.

Brehm (1933) created the genus *Argyrodiaptomus* and designated *Argyrodiaptomus granulosus* Brehm, 1933 as the type species. However, it is noteworthy that Wright (1927) had already identified what he called the “*bergi*” group, which would later be incorporated into the genus *Argyrodiaptomus*. Subsequently, in 1938, Wright reviewed the “*bergi*” group in South America, disagreeing with Brehm (1933) in certain respects, such as the inclusion of particular species and the use of the name of *Argyrodiaptomus*. However, the genus persists and is widely used today. The “*bergi*” group was centred on *Argyrodiaptomus bergi* (Richard, 1897). It is worth noting that both *Argyrodiaptomus bergi* and *Argyrodiaptomus granulosus* are relatively rare, and the latter has not been recorded since its original description (Brehm 1933).

*Notodiaptomus spiniger* has been considered by some researchers to belong to the genus *Argyrodiaptomus* (see Dussart and Defaye 2002), but it is clear that it does not fully conform to the diagnosis of either *Notodiaptomus* or *Argyrodiaptomus*. As currently constituted, it appears that *Notodiaptomus* is not a monophyletic taxon. It is possible that the generic concept should be restricted to fewer species, such as the *nordestinus* complex and perhaps a few other species distributed across the Brazilian shield and in the Amazon and de la Plata basins.

In the genus *Notodiaptomus* characters such as the presence or absence of spinular ornamentation on the dorsal surface of the pedigerous somites and its pattern and shape proved to be highly informative diagnostic features, thus facilitating the discrimination between certain pairs of species or closely related groups of species. The combination of this character with others (for example the state – complete or incomplete – of the suture between Ped4 and Ped5) can provide additional, robust criteria to achieve accurate identifications in this genus.

The fifth leg (P5) of adult diaptomids provides the main set of differential characteristics for most species. However, for some species of the *nordestinus* complex, for instance, the differences may be minimal, even with reference to the finest scale details.

Body length is useful for the identification of some species of *Notodiaptomus*. It can, for example, help to separate *Notodiaptomus conifer* from other species that share the possession of a small lateral spine on Exp2P5R, such as *Notodiaptomus iheringi* and *Notodiaptomus cearensis*. Some studies, such as Ringuelet and Martínez de Ferrato (1967), have highlighted the link between body size and the presence or absence of a falciform process on segment 20 of the maleA1R. According to their results, males with a well-developed process on the distal margin of segment 20 A1R, tend to be larger than those which lack this process. Males with well-developed process on segment 20, also tended to have a larger spinous process on segment 15. Laboratory experiments under various environmental conditions would be useful to clarify the significance of any possible linkage between the states of these two different characters. Such studies can also provide new insights into potential synonymies between species, as demonstrated by Ringuelet and Martínez de Ferrato (1967) who provided evidence to establish the synonymy between “Diaptomus” toldti and *Notodiaptomus spiniger* (as “Diaptomus” (Notodiaptomus) spiniger).

*Notodiaptomus incompositus* showed a distribution restricted to the middle and southern parts of the basin. This species shares several morphological characteristics with *Notodiaptomus deitersi* (Poppe, 1891), the type species of *Notodiaptomus*, which is included in the *nordestinus* complex. *Notodiaptomus spiniger* is not treated as part of this complex, even though it is also restricted to the south of the basin, as are *Notodiaptomus dentatus* and *Notodiaptomus carteri*, among others. Although *Notodiaptomus isabelae* occurred only in the middle and lower basin, there are several published records from the upper part of the Paraná River basin and from other smaller basins nearby within the Brazilian shield, for example, in the State of Minas Gerais, Brazil (Maia-Barbosa et al. 2008).

We did not find *Notodiaptomus deitersi* (Poppe, 1891) in this survey. The species was originally described from the region of Cuiabá (Mato Grosso State, Brazil) and it is the type species of *Notodiaptomus* (Santos-Silva et al. 1999). Among the adult diaptomids reported in this work, none showed the morphological characteristics of *Notodiaptomus dietersi* as presented by Santos-Silva et al. (1999) and a targeted search was carried out as we considered it highly likely that the species would occur in the basin. The morphotype of Matsumura-Tundisi (1986, 2008) is different from that redescribed by Santos-Silva et al. (1999), and was absent from our samples. There is the possibility that the sampling site visited during our survey may not have provided conditions conducive to the capture of this species.

**Figure 93.**
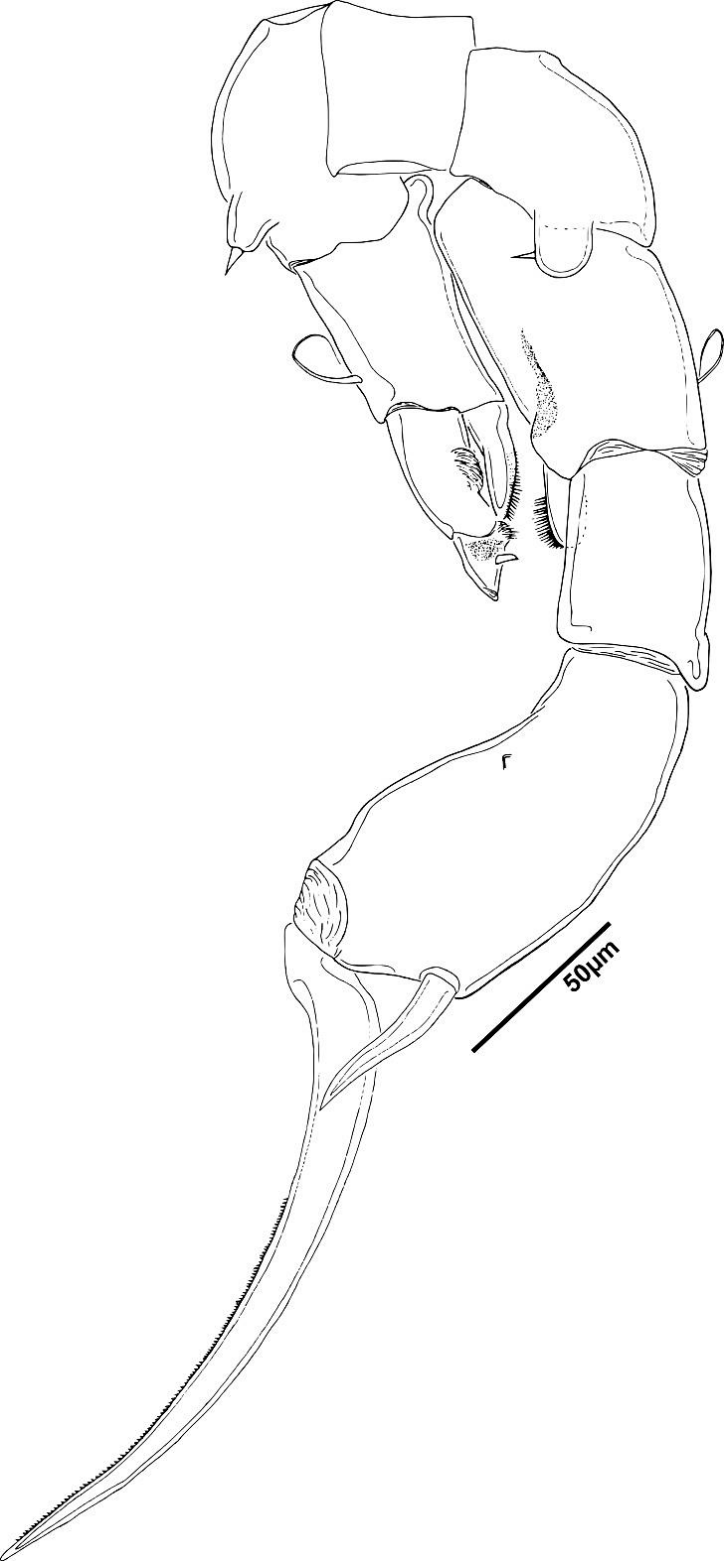
P5 of male Notodiaptomus cf. spinuliferus MZUSP (6971).

We decided to treat Notodiaptomus cf. spinuliferus as *incertae sedis* and not include it in this work because the vouchers deposited in MZUSP (6971) (Fig. 93), identified as Notodiaptomus cf. spinuliferus, are not sufficiently similar to the species descriptions provided by Dussart (1985), Dussart and Matsumura-Tundisi (1986), Paggi (2001) and Matsumura-Tundisi (2008). The specimens found in this study were more similar to those in Dussart (1985) and Paggi (2001). This species requires further taxonomic investigation in order to resolve its true identity.

As a result of previous faunistic surveys, some researchers have inferred that particular calanoid species have disappeared from the region (e.g. Matsumura-Tundisi and Tundisi 2003, Sartori et al. 2009). We consider that such inferences should be made with caution. In most cases there is little information on the population dynamics of the species and we found species such as *Odontodiaptomus thomseni* and *Argyrodiaptomus bergi* (Perbiche-Neves et al. 2011, 2012) which had not been seen for several decades. Our understanding of the relationships between diaptomids and the various limnological parameters and trophic status of water bodies is not very robust and is made less secure given that these species inhabit artificial reservoirs but were originally inhabitants of lakes, ponds, and rivers. Due to its large body size and susceptibility to being preyed upon by small fish, the abundance of species of *Argyrodiaptomus* is generally low in open water bodies when compared to that of other calanoids. During the counting of samples for ecological studies, if only low numbers of individuals (up to 200 per sample) are identified this probably decreases the chances of finding such rare taxa. In small, more closed habitats and in the absence of visual predators, the abundance of *Argyrodiaptomus* can be very high (Perbiche-Neves et al. 2011). *Argyrodiaptomus furcatus* and *Argyrodiaptomus azevedoi* occurred in reservoirs that were oligotrophic as well as in eutrophic habitats. They may be limited by turbidity rather than by trophic status due to their particular feeding habits. In addition to the transient negative effect on the water column of a turbidity event, after sedimentation of the suspended material, the surface of the substrate can be covered and may result in an adverse effect on the replacement rate of planktonic taxa by preventing hatching of deposited eggs. In such cases the recolonization after conditions have become favorable again may be by individuals whose origins are in associated lagoons or unaffected upstream river stretches.

*Notodiaptomus conifer* was reported as missing from Jurumirim Reservoir (Paranapanema River) by Matsumura-Tundisi and Tundisi (2003) and Sartori et al. (2009), but we found it in both the Jurumirim and Barra Bonita reservoirs. This species is distributed from northeast Brazil to Argentina, but may possibly occur at low densities, thus making the records of its presence unreliable for ecological studies, in which a standard volume of water is sampled from the surface or water column.

Taxonomically, the identification of *Notodiaptomus conifer* can be problematic. We recommend comparing the spinous process on segment 15 of the male A1R as well as considering body size. *Notodiaptomus conifer* has a greater body length than *Notodiaptomus iheringi* and other species, such as *Notodiaptomus henseni*, which could be confused with it. The presence of a row of spinules on the first segment of the A1R in *Notodiaptomus iheringi* allows this species to be distinguished from *Notodiaptomus conifer*. In *Notodiaptomus henseni*, the presence of sclerotized processes on the basipodites of both right and left P5 also avoids any confusion with *Notodiaptomus conifer*. In some *Notodiaptomus henseni* the presence of a well-developed spinous process on segment 15 of the A1R may cause confusion with *Notodiaptomus conifer* if this feature is considered alone, so it is always advisable to try to use more than one distinguishing feature, while also taking into account the known geographical distributions.

All these data indicate that care must be taken when reporting the disappearance of a particular species from a habitat; this conclusion should be drawn only after failure to find the species after continued sampling over extended periods of time. The probability of a species being found again seems high when routine sampling is used (e.g. Perbiche Neves et al. 2012). Another important issue is that adult diaptomids are scarce in lotic habitats, compared to lentic habitats. The macro-scale view obtained in this large scale survey supports such generalizations.

Studies carried out in Europe and Asia over long time periods have demonstrated that particular species may be dominant at rare intervals over a 20 year time series. Polli and Simona (1992) noted the complete disappearance of calanoids for nearly 30 years in a small lake, and then charted their re-appearance as the system returned to an oligotrophic state. If we analyze the historical data from some dam sites and other habitats in Brazil, for example, such as flood plains, we can observe that over 40 years, many species occur and then are absent for a period of time. During these intervals, the physical and chemical characteristics of the water and weather conditions may have changed and exerted a strong influence on the fauna. Straile and Geller (1998) studied the changes in the composition of microcrustaceans in Lake Constance between 1920 and 1995. During this period the lake was initially oligotrophic, became eutrophic, and then returned to oligotrophic status. Although the species richness found in a lake in temperate latitudes is lower than that found in a tropical lake or reservoir, this study illustrated important changes in species composition. Straile and Geller (1998) reported a major change after only a decade, in which the cyclopoid species *Cyclops vicinus* Uljanin, 1875, hitherto non-existent in the habitat, emerged as a common species. They attributed its success to the competitive advantages of this cyclopoid over calanoid copepodites (of the genus *Eudiaptomus* Kiefer, 1932), especially at high food concentrations. Predation by *Cyclops* on young stages of a predatory calanoid of the genus *Heterocope* G. O. Sars, 1863, led to the local extinction of *Heterocope* in the lake. Straile and Geller (1998) pointed out that *Heterocope* had also disappeared from other alpine lakes since 1950, but in many cases its disappearance was not associated with the presence of *Cyclops vicinus*. Finally, Straile and Geller (1998) compared the different sampling strategies in different years, concluding that the precise positioning of sampling locations (onshore versus offshore, for example) may have been an important factor influencing the analysis of species composition in the samples. The interactions within the zooplankton communities are complex and this is compounded by the greater species richness present in tropical regions.

Melo et al. (2006) indicated that the number of known species in Brazil can be mapped by their proximity to research centers. Our survey supports this conclusion, since many studies have been undertaken in southeastern and southern Brazil, primarily focusing on the more accessible reservoirs of the upper Paraná River and its floodplain. In Argentina, the same phenomenon was observed with studies being concentrated in the middle section of the river, mostly in marginal lakes and lotic stretches. So available data are still patchy as yet with little or no information on the copepods of particular sub-regions.

Within de la Plata river basin, the results obtained in this study combined with our review of published data suggest the following recommendations for future research: 1. Continue studying sites where historical collections have been made; 2. Study large and productive reservoirs for which little information is available and where new species records are likely; 3. Conduct studies in places that are unexplored and represent gaps in information, including reservoirs, ponds and wetlands adjacent to tributaries of the Upper Paraguay River, Upper Parnaíba River, Upper Uruguay River and west of the state of Rio Grande do Sul, the region of “Esteros de Iberá” (in Argentina); and 4. Study the transition areas between the northern and southern faunas, in the rivers Iguaçu, Paraná (Yaciretá Reservoir) and middle/upper Uruguay, focusing on flood periods and correlations with bird migration routes.

There are several taxonomic problems still to be resolved within the Neotropical diaptomids and it would be useful to test whether *Argyrodiaptomus* and *Odontodiaptomus* are monophyletic. Many species currently placed in *Notodiaptomus* and “*Diaptomus*” must eventually be relocated. *Diaptomus*, for example, continues to serve as a temporary repository for species whose affinities are as yet unresolved, such as the unrelated species “Diaptomus” frutosae and “Diaptomus” curvatus. We also consider that *Notodiaptomus spiniger*, *Notodiaptomus anisitsi* and *Notodiaptomus coniferoides* do not belong in *Notodiaptomus*. One solution might be the creation of additional new genera to accommodate some of these distinctive Neotropical diaptomids, however, new genera should only be erected after thorough phylogenetic analysis. Phylogenetic analyses based on morphological and molecular data are currently under way by the present authors and will be published elsewhere when completed.

The following genera and species should be reviewed as their taxonomic status is currently equivocal: *Notodiaptomus spiniger* (synonymy needs verification and its placement in *Notodiaptomus* requires testing); *Notodiaptomus spinuliferus* (the material deposited in MZUSP should be redescribed and compared with material found elsewhere and identified as *Notodiaptomus spinuliferus*); *Notodiaptomus amazonicus* from Amazonia should be compared with *Notodiaptomus amazonicus* from de la Plata river basin, including molecular analysis; the morphology of *Notodiaptomus coniferoides* should be checked by comparison with the holotype from the Amazon; this will provide a more robust basis for determining the true distribution of this species across the continent.

The rarity and seasonallity of some species in the basin, such as *Notodiaptomus anisitsi* and *Argyrodiaptomus falcifer*, and the absence of *Notodiaptomus deitersi*, *Odontodiaptomus paulistanus*, *Odontodiaptomus michaelseni*, and *Scolodiaptomus corderoi*, suggests that carrying out only two sampling programmes over a year may be inadequate in terms of recording sound data on the seasonal variability of these species. It is necessary to be very careful when using terms like “disappearing species”, since their absence might reflect an inadequate sampling regime.

## Supplementary Material

XML Treatment for Argyrodiaptomus azevedoi

XML Treatment for Argyrodiaptomus denticulatus

XML Treatment for Argyrodiaptomus falcifer

XML Treatment for Argyrodiaptomus furcatus

XML Treatment for Notodiaptomus anisitsi

XML Treatment for Notodiaptomus carteri

XML Treatment for Notodiaptomus cearensis

XML Treatment for Notodiaptomus conifer

XML Treatment for Notodiaptomus coniferoides

XML Treatment for Notodiaptomus dentatus

XML Treatment for Notodiaptomus henseni

XML Treatment for Notodiaptomus iheringi

XML Treatment for Notodiaptomus incompositus

XML Treatment for Notodiaptomus isabelae

XML Treatment for Notodiaptomus santafesinus

XML Treatment for Notodiaptomus spiniger

XML Treatment for Odontodiaptomus thomseni

XML Treatment for “Diaptomus” curvatus

XML Treatment for “Diaptomus” frutosae
